# Aggregation-induced emission luminogen in ternary organic bulk-heterojunction for efficient perovskite-organic tandem solar cells

**DOI:** 10.1038/s41467-026-73743-4

**Published:** 2026-05-30

**Authors:** Xiangyu Li, Mengzhen Du, Zongtao Wang, Qiang Guo, Tangyue Xue, Helin Wang, Jinglin Sun, Tingting Dai, Chenkai Sun, Zhi Zheng, Yanming Sun, Xing Feng, Hui Huang, Zhibin Yang, Erjun Zhou

**Affiliations:** 1 School of Chemistry and Chemical Engineering, Shanghai Jiao Tong University, Shanghai, China; 2 College of Biological and Chemical Engineering, Jiaxing University, Jiaxing, Zhejiang China; 3 Henan Institute of Advanced Technology, Zhengzhou University, Zhengzhou, China; 4 National Center for Nanoscience and Technology, Beijing, China; 5 College of Chemistry, and Green Catalysis Center, Zhengzhou University, Zhengzhou, China; 6 College of Chemical and Materials Engineering, Xuchang University, Xuchang, Henan China; 7 School of Chemistry, Beihang University, Beijing, China; 8 School of Material and Energy, Guangdong University of Technology, Guangzhou, China; 9 College of Materials Science and Opto-Electronic Technology, University of Chinese Academy of Sciences, Beijing, China

**Keywords:** Solar cells, Solar cells

## Abstract

Perovskite-organic tandem solar cells (TSCs) have recently garnered significant attention due to their potential for high power conversion efficiency (PCE) and excellent stability. However, their development has been significantly hindered by the large open-circuit voltage (*V*_OC_) deficit in organic sub-cells, primarily caused by severe non-radiative recombination, which is closely related to the electroluminescence quantum efficiency (EQE_EL_) and the photoluminescence quantum yield (PLQY). However, mainstream non-fullerene molecules exhibit low PLQY due to the aggregation-caused quenching (ACQ) effect. In this study, an aggregation-induced emission (AIE)-active molecule (TPE-BTA3) featuring a three-dimensional rotor-stereo configuration is rationally designed with an exceptional PLQY of 68%. When TPE-BTA3 is introduced into binary Organic solar cells (OSCs), it not only dramatically enhances the PLQY of alloy-acceptor but also strengthens the utilization of near-infrared photons, leading to a significant increase in *V*_OC_ and short-circuit current (*J*_SC_). By integrated above optimized ternary organic bulk-heterojunction with a wide-bandgap (1.85 eV) perovskite, the constructed perovskite-organic TSCs achieve a surprising PCE of 26.5% (certified as 25.8%). This work establishes a conceptual bridge between high-efficiency photovoltaics and AIE molecular design paradigms.

## Abstract (web summary)

Perovskite–organic tandem solar cells promise very high efficiency, but progress is limited by voltage loss from non-radiative recombination in organic layers. Li et el. design an emission-active molecule that suppresses quenching, improves light use, and enables high efficiency tandem devices.

## Introduction

Organic solar cells (OSCs), as a third-generation photovoltaic technology, boast advantages such as lightweight, flexible, semi-transparent, and capable of low-temperature solution processing and large-area “roll-to-roll” printing^1–4^. The advent of Y-series nonfullerene acceptors, represented by Y6^5^, has led to a dramatic improvement in the power conversion efficiency (PCE) of OSCs over 20%^6,7^. Y-series organic semiconductors with a band gap of 1.3–1.4 eV enable them to be particularly suitable for constructing perovskite-organic tandem solar cells (TSCs), which have unique advantages among various perovskite-based TSCs. The perovskite-silicon TSCs offer ideal bandgap matching, high power conversion efficiency, and accessible rapid commercialization via the mature silicon photovoltaic industrial chain, but their inherent rigidity limits applications in flexible and lightweight scenarios. All-perovskite TSCs enable low-temperature solution processing and flexible device fabrication, yet the easy oxidation of tin-based narrow-bandgap perovskites severely impedes their commercial feasibility. By contrast, perovskite-organic TSCs inherit the core advantages of all-perovskite TSCs, including solution processability and excellent mechanical flexibility. Notably, the use of mutually orthogonal solvents in fabrication circumvents the need for complex techniques like atomic layer deposition, simplifying manufacturing^8,9^. Admittedly, the current efficiency of such devices is limited by the performance of narrow-bandgap organic materials, but with the design and synthesis of new high-performance organic photovoltaic materials, perovskite-organic TSCs hold extremely promising development prospects.

Recent advancements in perovskite-organic TSCs have achieved remarkable progress, with the highest certified PCE reaching 26.4%^8–17^. Nevertheless, a notable performance gap persists when compared to all-perovskite TSCs^18^, primarily attributed to two fundamental limitations in the organic sub-cell: the substantial energy losses and inadequate photon harvesting efficiency at near-infrared regions. Addressing these challenges is a critical pathway for the efficiency improvement of perovskite-organic TSCs.

According to the detailed balance theory, the energy loss in OSCs is typically categorized into three components, Δ*E*_1_, Δ*E*_2,_ and Δ*E*_3_. The Δ*E*_1_ usually means the loss stemming from radiative recombination above the bandgap; The Δ*E*_2_ can be seen as the additional radiative recombination loss below the optical gap; and the Δ*E*_3_ is often regarded as the loss attributed to non-radiative recombination. The reported OSCs results indicated that Δ*E*_3_, typically in the range of 0.2–0.4 eV, accounts for the bulk of energy loss for OSCs^19,20^. According to the reciprocity relation between photovoltaic quantum efficiency and electroluminescent emission of solar cells, Δ*E*_3_ = −*kT*ln (EQE_EL_), where EQE_EL_ is the electroluminescence quantum efficiency of solar cells under darkness by injecting charge carriers into the device^21^. Furthermore, EQE_EL_ = *γχ*Φ_PL_*η*_out_^22^, where *γ* is the charge balance factor, *χ* is the fraction of recombination events due to radiative decay, Φ_PL_ is the photoluminescence quantum efficiency, and *η*_out_ is the photon out-coupling efficiency^23^. In OSCs, the photoluminescence quantum yield (PLQY) of narrow bandgap materials in the active layer sets the upper limit of the Φ_PL_ for devices. Therefore, it is theoretically feasible that enhancing the PLQY of narrow bandgap materials (usually nonfullerene acceptors) to increase the EQE_EL_ of the device and obtain a smaller Δ*E*_3_^24^. However, current nonfullerene acceptors always exhibit a serious aggregation-caused quenching (ACQ) phenomenon in the film state, resulting in a low PLQY^25^. For instance, the PLQY of ITIC series molecules is only about 1%^26^, and the new-generation Y-series molecules are slightly higher at around 5%^27^. Thus, efforts to alleviate the ACQ phenomenon and improve their PLQY of nonfullerene acceptors are promising and feasible strategies^28,29^.

Based on the above issues, aggregation-induced emission luminogen (AIEgen) characterized with rotor stereo structures and strong emissions in the aggregated or solid-state has popped into our head, which exhibits ultra-high PLQY once the intramolecular rotations or vibrations are restricted^30,31^. In early studies, there have been several reports trying to directly design host electron acceptor materials with some classic AIE construction units, such as tetraphenylethylene (TPE) and triphenylamine (TPA)^32–37^. However, the final molecules produce relatively low PCEs. For example, in 2015, Yan et al. reported a small molecule based on TPE and perylene diimide (PDI), and the final TPE-PDI4 realized a PCE of 5.5% when blended with PTB7-Th. Thereafter, they changed the middle AIE unit from TPE to tetraphenyl pyrazine (TPPz) and improved the PCE to 7.1%^37^. The ternary blend method is a universal morphology control method to improve the OSCs’ efficiency by introducing a guest component, and AIEgen has also been preliminarily tried as a guest molecule for ternary OSCs. In 2020, Wei et al. directly utilized the simple TPE molecule to modulate the film morphology of the J71:ITIC blends, achieving an improved PCE from 10.03% to 12.16%^38^. These initial attempts suggest that AIEgen has a large potential to decrease energy loss and improve efficiency, but delicate molecular design and related device mechanisms need to be studied in depth.

We have long been committed to developing non-fullerene small molecules based on benzotriazole (BTA) units. In our previous work, BTA3 has demonstrated a significant effect in reducing voltage loss^39,40^. Building on this foundation, we designed TPE-BTA3 by integrating the classic BTA3 unit with TPE, in order to achieve better photovoltaic performance. We incorporated TPE-BTA3 into the classic PM6:BTP-eC9 bulk-heterojunction^41^, and observed a suppression in nonradiative recombination energy loss and a promotion in the utilization of near-infrared photons, which leads to a significant photovoltaic performance improvement. Through research on alloy-acceptor, energy transfer, photoluminescence quantum yield, energy loss, and aggregation improvement, we have identified the mechanism for improving ternary cells’ performance. Finally, by combining this ternary organic bulk-heterojunction with a WBG perovskite, we further fabricated perovskite-organic TSCs and achieved a high PCE of 26.5% (certified as 25.83%).

## Results

**Fig. 1:**
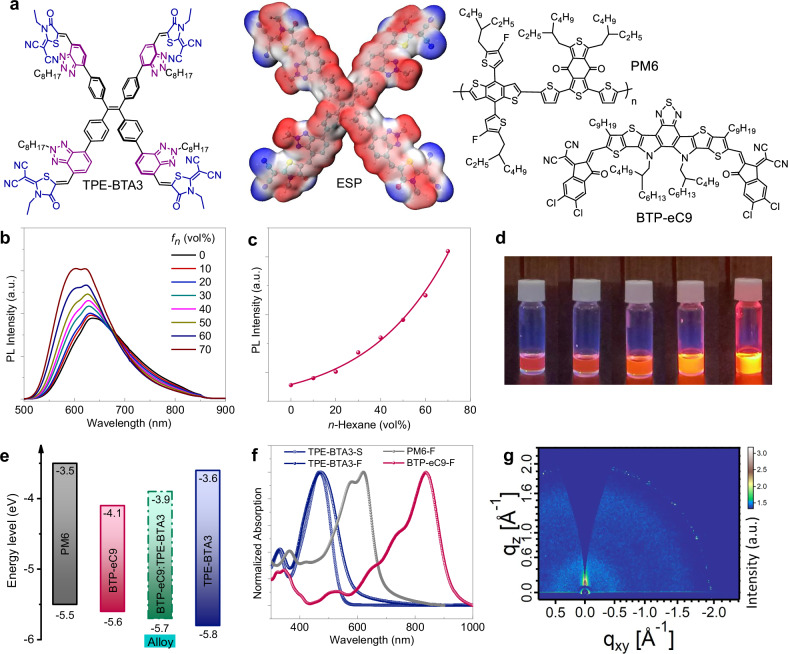
Basic information of TPE-BTA3, PM6, and BTP-eC9. **a** Chemical structure of TPE-BTA3, PM6 and BTP-eC9, ESP structure of TPE-BTA3. **b** PL spectra at varying solvent volume fractions measured with an OD 2.0 filter. **c** Dependence of PL peak intensity on solvent volume fraction. **d** Photo of TPE-BTA3 solutions with varying solvent composition under a UV lamp, the concentration was increased tenfold for a more distinct visual contrast. **e** Energy level diagram of TPE-BTA3, PM6, and BTP-eC9. **f** Normalized absorption spectra of TPE-BTA3 in solution (S) and film (F) states, and the absorption spectra of PM6, BTP-eC9 in film state. **g** 2D GIWAXS image of TPE-BTA3, the color bar represents intensity in arbitrary units.

The synthesis of TPE-BTA3 and its structural characterizations (^1^HNMR and ^13^CNMR) are detailed in the Supporting Information (Supplementary Fig. 1, 2, 3, Supplementary Note 1). TPE-BTA3 exhibits good solubility in common processing solvents such as chloroform, chlorobenzene, tetrahydrofuran, and o-xylene. The chemical structure and the distribution of the surface electrostatic potential (ESP)^42^ of TPE-BTA3 are depicted in Fig. 1a, while the optimal geometries, calculated using density functional theory (DFT) (the long alkyl chains simplified to methyl for ease of calculation), are presented in Supplementary Fig. 4. The central core of TPE-BTA3 is TPE, with each benzene ring of TPE having an “arm” (RCN-modified BTA unit) connected to its para-position. The central core can rotate due to the single bond, and the nucleus and the arms are also connected through single bonds, thus permitting intramolecular rotation. The ESP diagram illustrates the push-pull electron effect clearly and also visualizes the existence of sufficient rotatable space within the molecule when the molecule is in the lowest energy configuration. This suggests that when the molecules are far away from each other, they can easily consume energy by intramolecular rotation after being excited, which is initially consistent with the theory of AIE.

To verify whether TPE-BTA3 possesses AIE properties, we dissolved TPE-BTA3 in a chloroform (good solvent)/n-hexane (poor solvent) mixed solvent with varying volume ratios, and regulated the aggregation of TPE-BTA3 by changing the volume fraction of the poor solvent. As shown in Fig. 1b, c, the photoluminescence (PL) intensity of TPE-BTA3 increased rapidly as the volume fraction of the poor solvent (*f*_*n*_) increased from 0% to 70% (with a constant concentration of 0.001 mol/L), which was accompanied by a gradual blueshift of the PL spectrum. From Fig. 1d, it can be observed that TPE-BTA3 gradually emits strong fluorescence with the increase of aggregation degree under ultraviolet light irradiation. We also tested the particle size change of TPE-BTA3 under different *f*_*n*_ (Supplementary Fig. 5). The average particle size of nanoaggregates was 63.9 nm at 20% *f*_*n*_, while it increased to 305 nm at 70% *f*_*n*_, which confirms the aggregation behavior of TPE-BTA3. Lastly, we tested the PLQY change of TPE-BTA3 at different *f*_*n*_. The PLQY of TPE-BTA3 is 37.3% when *f*_*n*_ is 0%, and it grows to 55.7% when *f*_*n*_ rises to 70%.

To further verify the AIE effect, we performed additional photoluminescence measurements under enhanced excitation light intensity. As illustrated in Supplementary Fig. 6, when the excitation light intensity was increased tenfold with an OD 1.0 filter, the fluorescence emission intensity of TPE-BTA3 still exhibited a clear enhancement upon increased aggregation. The PLQY value of TPE-BTA3 increased from 35.7% to 54.5% as the water fraction *f*_n_ rose from 0% to 70%. It should be noted that under high-intensity excitation, AIE-active molecules may undergo excited-state absorption, which can introduce extra non-radiative decay channels and thus negatively affect the PLQY. Despite this effect, the above results clearly confirm that TPE-BTA3 exhibits distinct AIE behavior, which is not dependent on the excitation intensity.

In a dilute solution, the TPE-BTA3 molecules are dispersed, allowing for easy energy dissipation through single-bond rotation post-excitation. This process significantly suppresses the energy-consuming way of fluorescence emission, resulting in weak or even non-existent luminescence. Conversely, when the degree of molecular aggregation is enhanced by increasing *f*_*n*_, the molecules aggregate, severely limiting intramolecular rotation. This restriction inhibits the non-radiative energy-consuming mode, causing most of the energy to be released via fluorescence emission. Consequently, the intensity of the PL spectra increases, accompanied by a blueshift.

To explore the potential of TPE-BTA3 as a third component in ternary OSCs, we first investigated the physicochemical properties of TPE-BTA3. The energy levels of TPE-BTA3 were determined by cyclic voltammetry (CV), revealing the lowest unoccupied molecular orbital (LUMO) and highest occupied molecular orbital (HOMO) energy levels of −3.6 eV and −5.8 eV, respectively (Fig. 1e and Supplementary Fig. 7). It is noteworthy that the LUMO energy level of TPE-BTA3 is positioned between those of PM6 and BTP-eC9, thereby establishing a cascade energy level alignment. This configuration is beneficial for promoting charge transfer in ternary cells. Upon measuring the energy levels of the TPE-BTA3:BTP-eC9 mixture, we observed an intermediate result between those of the individual components, indicating the potential formation of an alloy-acceptor^43^. We proceeded to investigate the miscibility between the three materials, and the water contact angle and n-hexadecane contact angle are shown in Supplementary Fig. 8 and Supplementary Table 1. The surface tension (γ) values were calculated to be 20.7, 23.6, and 25.5 mN m^−1^ for PM6, BTP-eC9, and TPE-BTA3, respectively. According to the Flory–Huggins interaction parameter formula, $\chi={\left(\sqrt{{\gamma }_{A}}-\sqrt{{\gamma }_{B}}\right)}^{2}$, the *χ* values of TPE-BTA3 with PM6 or BTP-eC9 are 0.25 and 0.04 mN m^−1^. The relatively small *χ* value between TPE-BTA3 and BTP-eC9 suggests good miscibility, and they are more inclined to form an alloy-acceptor.

To investigate the intermixing behavior of TPE-BTA3 and BTP-eC9, we first performed thermogravimetric analysis (TGA) on pure BTP-eC9, pure TPE-BTA3, and the BTP-eC9:TPE-BTA3 blend (10:1 weight ratio, consistent with the ternary device composition). As shown in Supplementary Fig. 9, the thermal decomposition temperatures (T_d_, corresponding to 5% weight loss) were measured to be 332, 401, and 337 ^o^C for BTP-eC9, TPE-BTA3, and their blend, respectively. Notably, no additional decomposition peaks were observed in the thermogram of the blend, suggesting homogeneous molecular-scale mixing between the two components, which is conducive to the formation of an alloy-acceptor.

We systematically investigated the absorption spectra of TPE-BTA3 in both solution and thin-film states (Fig. 1f), revealing distinct absorption peaks at 468 nm and 472 nm, respectively, with a corresponding optical bandgap of 2.15 eV. The spectral response of TPE-BTA3 exhibits excellent complementarity with the PM6:BTP-eC9 blend, enabling efficient utilization of the solar spectrum. Notably, TPE-BTA3 did not exhibit a significant redshift of the absorption peaks in the film compared to the solution, which may be attributed to its highly distorted structure with weak intermolecular interactions and aggregation behaviors. Grazing incidence wide-angle X-ray scattering (GIWAXS) tests (Fig. 1g) confirmed that the stacking of TPE-BTA3 in the film is highly disordered, with weak packing strength and no obvious orientation. This unusual packing behavior of TPE-BTA3 allows it to effectively influence the stacking of BTP-eC9 after forming an alloy-acceptor, and further affect the utilization of photons in the near-infrared region.

**Fig. 2:**
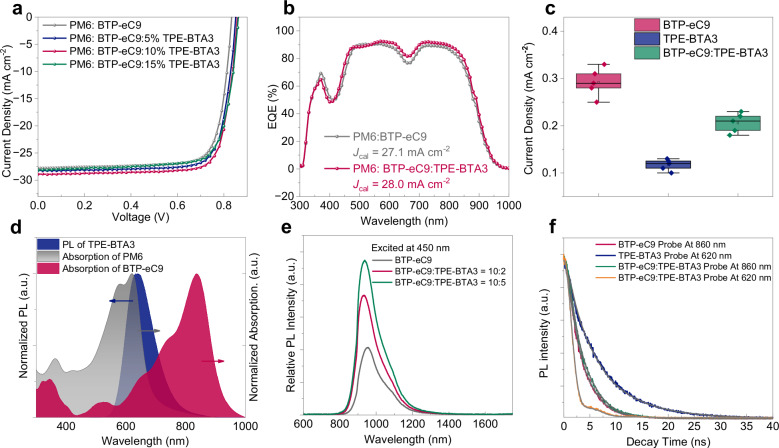
Photovoltaic performance and basic interaction mechanism of PM6:BTP-eC9:TPE-BTA3 bulk-heterojunction. **a** Current density–voltage curves (*J*–*V)* of binary and ternary devices with varying ratios of TPE-BTA3. **b** EQE curves of control binary and optimized ternary devices. **c** *J*_SC_ values and variance (error bars) of different film-based devices. **d** PL spectra of TPE-BTA3, absorption spectra of PM6, and BTP-eC9. **e** PL spectra of pure films and blend films that were excited at 450 nm. **f** TRPL decay and fitted curves (gray) of BTP-eC9, TPE-BTA3, and their blend film that were probed at 620 nm and 860 nm.

Following the above initial investigation, we found that TPE-BTA3 is compatible with the PM6:BTP-eC9 binary bulk-heterojunction in terms of both energy level and absorption spectra. The disordered stacking of TPE-BTA3 may effectively modulate the aggregation behavior of BTP-eC9 after forming an alloy-acceptor, and TPE-BTA3, with its high PLQY, may enhance the luminescence performance of the alloy-acceptor, thus reducing non-radiative energy loss. Therefore, introducing TPE-BTA3 as a third component into PM6:BTP-eC9 bulk-heterojunction holds significant promise for enhancing device performance. Subsequently, we fabricated binary and ternary OSCs with the device structure of ITO/2-PACz/active layer/PNDIT-F3N/Ag. As shown in Fig. 2a and Supplementary Table 2, the control binary OSC exhibited a PCE of 18.3% with a *V*_OC_ of 0.83 V, a *J*_SC_ of 27.8 mA cm^−2^, and a FF of 78.7%. The incorporation of TPE-BTA3 resulted in a significant enhancement in photovoltaic performance. The optimal TPE-BTA3 concentration was determined to be 10%, with the corresponding device achieving a remarkable PCE of 19.7%, accompanied by a *V*_OC_ of 0.860 V, a *J*_SC_ of 28.9 mA cm^−2^, and a FF of 79.4%. Figure 2b presents the EQE curves of the control binary and optimized ternary devices. Obviously, the ternary device exhibits higher response values after 550 nm, unlikely to be an additional absorption directly brought by TPE-BTA3, which is presumably caused by the improvement of the stacking behavior of PM6:BTP-eC9 from TPE-BTA3. The slight reduction of optical response within 350–400 nm in the ternary device may be attributed to the relatively weak absorption of TPE-BTA3 in this wavelength range. To more precisely determine the photoelectronic conversion efficiency, the internal quantum efficiency (IQE) was calculated by accounting for reflection and parasitic absorption losses. As shown in Supplementary Fig. 10, the incorporation of TPE-BTA3 obviously enhances IQE within the wavelength ranges of 400–500 nm and 600–900 nm. Further investigation is required to elucidate these details, which will be discussed in a later section.

We first investigated the basic interaction mechanisms of TPE-BTA3 in the ternary device. To determine whether charge transfer occurs between TPE-BTA3 and BTP-eC9, OSCs were fabricated using BTP-eC9, TPE-BTA3, and their blend as active layers. The measured *J*_SC_ values were 0.29, 0.12, and 0.20 mA cm⁻², respectively (Supplementary Fig. 11, the statistical average data are shown in Fig. 2c). The *J*_SC_ of the blend-based device is intermediate between those of the devices based on the pure BTP-eC9 and TPE-BTA3 films, indicating no obvious charge transfer occurs between TPE-BTA3 and BTP-eC9. Subsequently, we noted a large overlap between the PL spectra of TPE-BTA3 and the absorption spectra of PM6 and BTP-eC9 (Fig. 2d), implying that there may be an energy transfer from TPE-BTA3 to PM6 or BTP-eC9. To probe this mechanism, we conducted steady-state and time-resolved PL (TRPL) spectra analyses. We prepared pure films of TPE-BTA3, PM6, and BTP-eC9, and blended films of PM6:TPE-BTA3 and BTP-eC9:TPE-BTA3 in two ratios (10:2 or 10:5) with a 450 nm laser excitation to ensure that all the molecules were in the excited state (Fig. 2e, Supplementary Fig. 12). The results revealed that the PL signals of either PM6 or BTP-eC9 in the blended films were significantly stronger than those of the pure films, and the PL intensity increased with the doping ratio of TPE-BTA3. Moreover, the PL signal of TPE-BTA3 within the mixed films almost disappeared compared to the pure film (Supplementary Fig. 13, the PL intensity is exceptionally high and thus presented separately), suggesting that almost all fluorescence emitted by TPE-BTA3 can be absorbed by PM6 or BTP-eC9. But in the PM6:TPE-BTA3 blend film, due to the proximity of the fluorescence peak of PM6 (670 nm) and TPE-BTA3 (620 nm), a coupling peak may be formed and interfere with the judgment. Therefore, we measured the fluorescence lifetimes of the corresponding molecules within the pure and blend films (10:5 ratio) by TRPL (Fig. 2f, Supplementary Fig. 14, and Supplementary Table 3), and discovered that the fluorescence lifetimes of TPE-BTA3 in the blend films were drastically shortened compared with the pure films (2805 ps *vs*. 305 ps or 150 ps), while the fluorescence lifetimes of PM6 and BTP-eC9 were prolonged (275 ps *vs*. 550 ps and 1099 ps *vs*. 1173 ps). At this point, we have established that there is an energy transfer from TPE-BTA3 to PM6 or BTP-eC9, which provides an additional charge generation pathway within the ternary device and subsequently will contribute to the enhancement of the alloy-acceptor’s PLQY.

Energy transfer is categorized into Förster resonance energy transfer (FRET) and Dexter energy transfer (DET). FRET is a non-contact, long-range process mediated by dipole–dipole interactions, typically effective over distances of 1–10 nm. It requires substantial spectral overlap between the donor’s emission and the acceptor’s absorption, as well as a high PLQY of the donor. In contrast, DET is a short-range, contact-dependent process involving electron exchange, with an effective distance of less than 1 nm. It necessitates direct orbital overlap between donor and acceptor and matching spin states. In the PM6:BTP-eC9:TPE-BTA3 system studied here, TPE-BTA3 serves as the energy donor and exhibits a high PLQY. Its fluorescence emission spectrum shows significant overlap with the absorption spectra of both PM6 and BTP-eC9, as illustrated in Fig. 2d. Based on these characteristics, we propose that FRET should be the dominant mechanism in this system.

The influence of TPE-BTA3 on energy loss, particularly non-radiative energy loss, needs to be studied in depth, which can provide important guidelines for the selection of AIEgens that can be used in OSCs. In OSCs, the PLQY of low bandgap materials sets the upper limit for the Φ_PL_ of devices^24^. Therefore, we first examined the impact of TPE-BTA3 on PLQY. We analyzed the signal of BTP-eC9 within both pure BTP-eC9 film and a blend film of BTP-eC9:TPE-BTA3 (10:1 ratio) to investigate the changes in PLQY of BTP-eC9 itself. For this, we used a 750 nm laser to ensure that TPE-BTA3 was in the ground state and to prevent energy transfer. BTP-eC9, with its extensive π–π conjugation and planarization, exhibits strong intermolecular π–π interactions, making it prone to non-radiative excitation electron transfer, leading to ACQ^25^. Consequently, the PLQY of BTP-eC9 is a mere 6.43%. However, the highly distorted structure of TPE-BTA3 that is dispersed in BTP-eC9 can inhibit its over-aggregation (proved by later morphology analysis), thereby reducing the ACQ effect^28,29^. This led to an increase in the PLQY of the BTP-eC9:TPE-BTA3 alloy–acceptor to 10.35%. The PLQY of the TPE-BTA3 pure film was remarkably high at 68.44%, and we were intrigued to see if this high PLQY could be maintained after dispersing it into other materials at a 10% ratio. Due to the energy transfer from TPE-BTA3 to BTP-eC9, we replaced BTP-eC9 with polystyrene (PS), which is non-absorbing in the visible region, to prepare a PS:TPE-BTA3 (10:1 ratio) blend film. The PLQY of PS:TPE-BTA3 was found to be 55.35%, indicating that although TPE-BTA3 was dispersed, mechanisms such as intramolecular rotation or vibration restriction are still operational, allowing TPE-BTA3 to maintain a substantial PLQY. Finally, TPE-BTA3 can both alleviate the ACQ effect of BTP-eC9 and transfer energy to it, the PLQY of the BTP-eC9:TPE-BTA3 alloy-acceptor (10:1 ratio, excited at 500 nm) was measured to be 14.75% (Supplementary Fig. 15). It should be noted that, although TPE-BTA3 has a very high PLQY, most of the photons and the fluorescence emitted by TPE-BTA3 (through energy transfer) will be absorbed by BTP-eC9, and cannot continue to be effectively converted into fluorescence emission. Obviously, the alloy acceptor in the ternary device has a significant improvement in PLQY compared to BTP-eC9 alone (6.43%), and theoretically, the EQE_EL_ of the ternary device will be significantly improved.

**Fig. 3:**
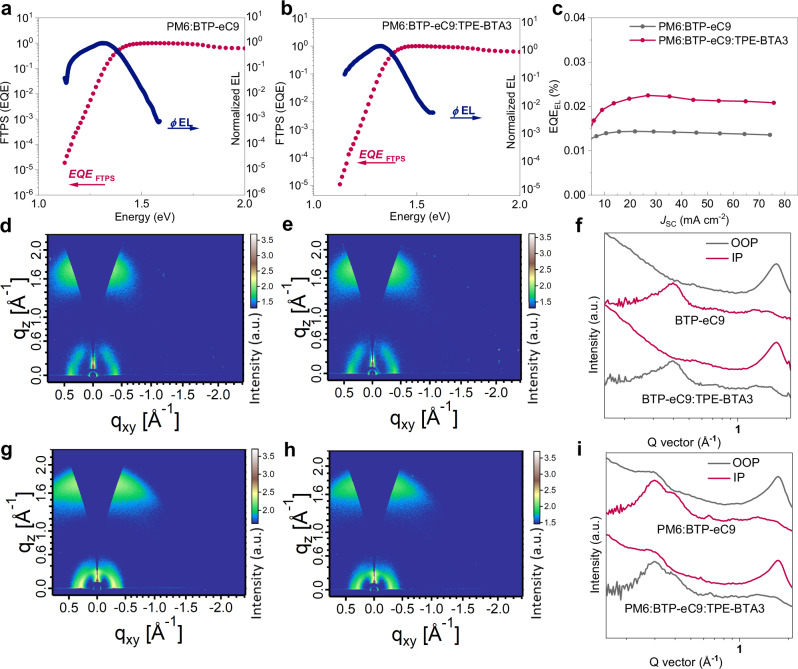
Energy loss and GIWAXS data of PM6:BTP-eC9, and PM6:BTP-eC9:TPE-BTA3. **a**, **b** Normalized FTPS-EQE spectra, EL spectra of PM6:BTP-eC9 (**a**), and PM6:BTP-eC9:TPE-BTA3 (**b**). **c** EQE_EL_ curves. **d**, **e**, **g**, **h** 2D GIWAXS images of BTP-eC9; the color bar represents intensity in arbitrary units (**d**), BTP-eC9:TPE-BTA3 (**e**), PM6:BTP-eC9 (**g**), and PM6:BTP-eC9:TPE-BTA3 (**h**). **f**, **i** 1D X-ray profiles in the OOP and IP directions of the above films.

We then formally performed an energy loss analysis according to Supplementary Note 2. The highly sensitive EQE (s-EQE) and electroluminescence (EL) spectra of PM6:BTP-eC9 and PM6:BTP-eC9:TPE-BTA3 are depicted in Fig. 3a, b, with detailed voltage loss data calculated according to the SQ limit presented in Supplementary Table 4. The band gaps are identical in both binary and ternary devices. Similarly, the value of ΔE₁ remains the same (0.26 eV) in both systems. In contrast, the Δ*E*₂ value for the ternary device (0.052 eV) is slightly lower than that of the binary device (0.057 eV). As illustrated in Fig. 3c, the EQE_EL_ of the ternary device is markedly higher than that of the binary device (2.24 ×;10^−4^ *vs*. 1.43 × 10^−4^, which is an increase of more than 50% and consistent with the trends in PLQY), leading to a reduction in Δ*E*_3_ from 0.229 eV to 0.217 eV. To accurately assess the energy losses in the devices, we statistically analyzed the Δ*E*_3_ and Δ*E*_2_ based on five groups of devices, as summarized in Supplementary Table 5. The average Δ*E*_3_ values for the ternary and binary devices are 0.229 ± 0.001 and 0.218 ± 0.001 eV, respectively, while the corresponding Δ*E*_2_ values are 0.057 ± 0.001 eV and 0.052 ± 0.001 eV. So far, we have successfully demonstrated from theory to practice that TPE-BTA3 can reduce the non-radiative recombination energy loss.

We were also interested to know the energy loss of the binary device that uses TPE-BTA3 as the only acceptor to understand the development dilemma of AIEgen-based binary OSCs, so the optimized PM6:TPE-BTA3 device was tested (Supplementary Fig. 16). Owing to the substantial increase in the bandgap, Δ*E*_1_ escalated to 0.291 eV. However, Δ*E*_2_ and Δ*E*_3_ also exhibited unusually high values of 0.126 and 0.245 eV, details see Supplementary Table 6. As previously confirmed, the structure of TPE-BTA3 is highly distorted, with weak and disordered stacking in the film, which is not favorable for the formation of continuous and efficient charge transport channels. Therefore, it is highly probable that the charge transport in the TPE-BTA3 phase region is severely hindered, leading to intense charge recombination and abnormally large Δ*E*_2_ and Δ*E*_3_. By analyzing the *V*_OC_ and *J*_SC_ of PM6:TPE-BTA3 at varying light intensities, we calculated the parameters *n* and *α*, which reflect the trap-assisted recombination and bimolecular recombination, respectively (*n* = 1.22, *α* = 0.88, markedly inferior compared to the subsequent normal devices). This further confirms the presence of severe charge recombination within the TPE-BTA3-based binary device. To summarize the above energy loss, following the formation of the alloy–acceptor, BTP-eC9 not only achieved a higher PLQY due to the mitigation of the ACQ effect, but TPE-BTA3 also managed to maintain a very high PLQY under the dispersed state. Furthermore, TPE-BTA3 transferred energy to BTP-eC9, significantly enhancing the PLQY of the alloy–acceptor, which ultimately reduced the Δ*E*_3_ of ternary devices.

The application of AIE molecules in OSCs requires careful tailoring to device configurations and functional demands. In ternary blends with a small AIE component, the emphasis is on achieving high luminescence efficiency. However, in binary systems where the AIE molecule acts as the sole donor or acceptor, stricter structural control is essential; the AIE characteristic should not originate from the central core, and the appropriate side-chain modifications are required to preserve the intrinsic ordered π–π stacking necessary for efficient charge transport.

The influence of TPE-BTA3 on film morphology was further explored by GIWAXS. Initial experiments were performed on BTP-eC9 pure film and BTP-eC9:TPE-BTA3 blend film (10:1 ratio). As depicted in Fig. 3d, e, both the pure and blend films exhibited a clear (010) diffraction peak in the out-of-plane (OOP) direction and a (100) diffraction peak in the in-plane (IP) direction. These peaks signify a face-on stacking orientation, suggesting that the stacking orientation of BTP-eC9 remained unaltered by TPE-BTA3. 1D X-ray diffraction analysis (Fig. 3f and Supplementary Table 7) reveals a slightly greater π–π stacking distance (*d*_π–π_) in the blend film compared to the pure BTP-eC9 film. Simultaneously, the crystal coherence length (CCL) of the (010) peak decreased from 14.70 Å to 14.32 Å. And the same phenomenon can be found for the (100) peaks. These modifications strongly suggest that the incorporation of TPE-BTA3 dispersed the π–π stacking of BTP-eC9 and suppressed its over-aggregation^44^, aligning with the previous analysis of the energy loss part. Additionally, the BTP-eC9:TPE-BTA3 blend film maintained a single peak shape while exhibiting peak position shifts, further corroborating the formation of an alloy phase. A distinct blue shift in the 650–800 nm range was also observed after adding TPE-BTA3 into the BTP-eC9 film (Supplementary Fig. 17). The difference between the binary/ternary films became faint, we proceeded to calculate the stacking and crystallization data within the binary and ternary films by GIWAXS data (Fig. 3g–i), the results indicated that the ternary films also possessed a larger d_π__–π_ and a smaller CCL. Thus, TPE-BTA3 can still effectively improve the molecular packing equilibrium and inhibit the over-aggregation of BTP-eC9 in the ternary devices. The modified microstructure can reduce charge recombination and increase charge extraction, which is conducive to enhancing the utilization of photons in the near-infrared range and obtaining a higher *J*_SC_.

The surface morphology of the films was also examined using atomic force microscopy (AFM) (Supplementary Fig. 18). The test results showed that the root-mean-square (RMS) roughness of the binary films was 3.74 ± 0.04 nm, and that of the ternary films was 3.82 ± 0.03 nm. The RMS increase might be attributed to the disordered structure of the TPE-BTA3 that dispersed on the film surface, a phenomenon similarly reported in some studies of “multi-arm small molecule” as the third component^43,45^. In addition, the phase image revealed a clear fiber-like structure in the ternary film, which is beneficial for charge transport.

Device physics was also a subject of our analysis. Initially, we examined the hole transfer from BTP-eC9 to PM6 by PL (Supplementary Fig. 19). Upon excitation at 750 nm, the fluorescence signals of BTP-eC9 within both binary and ternary blend films were significantly quenched (by over 95%), indicating an effective hole transfer in both. We performed a more precise study by using transient absorption spectroscopy (TAS) tests. As shown in Supplementary Figs. 20 and 21, the ground-state bleach (GSB) signal of PM6 was observed at 630 nm in both binary and ternary films (excited at 750 nm), which also suggests effective hole transfer.

We also procured the *J–V* curves of the devices in the dark state (Supplementary Fig. 22), the series resistance (*R*_s_), shunt resistance (*R*_sh_), and ideality factor (*n*_0_) were extracted from regions I, II, and III. The ternary device has a smaller leakage current in Region I, a larger slope in Region II, and a higher rectification ratio, advantageous for achieving higher *J*_SC_ and FF^46^. Then we measured the charge mobility of binary and ternary films by the space charge limited current (SCLC) methods, and the relationship between current and voltage in the dark state and the corresponding fitted curves are shown in Supplementary Figs. 23 and 24. The hole and electron mobility of the ternary devices are both enhanced, and the charge transport is more balanced (*μ*_h_/*μ*_e_ of 1.44 and 1.29, detailed data refer to Supplementary Table 8), which is favorable for obtaining better photoelectric parameters. We then measured the *J–V* characteristics of binary and ternary devices at varying light intensities (*P*_light_) to further corroborate the charge recombination. As shown in Supplementary Fig. 25, after fitting, the n values of the binary and ternary devices at room temperature are 1.10 and 1.03. The closer the n value is to 1, the weaker the charge recombination due to the trap. The values of α for binary and ternary devices are calculated to be 0.992 and 0.995, and the larger value of *α* indicates the weaker bimolecular recombination. Thus, ternary devices exhibit weaker trap-assisted recombination and bimolecular recombination compared to binary devices.

**Fig. 4:**
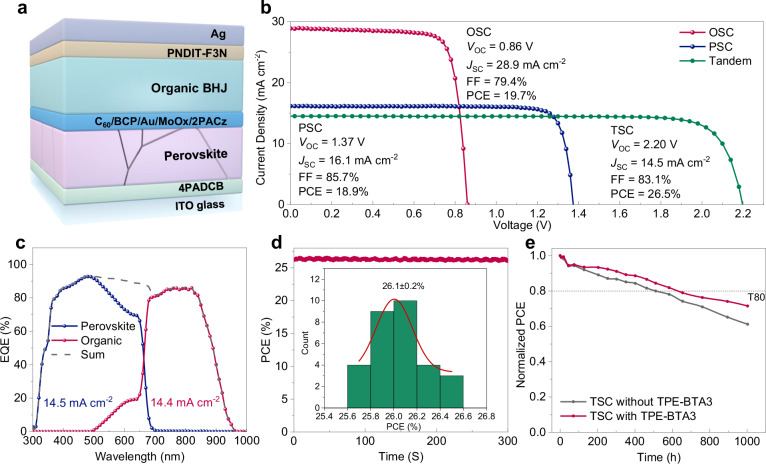
Photovoltaic performance of the perovskite-organic TSCs. **a** Schematic illustration of a perovskite-organic TSC. **b** *J*–*V* curves of OSCs, PSCs, and perovskite-organic TSCs measured under AM1.5 G illumination. **c** EQE curves of the front and rear sub-cells in the perovskite-organic TSCs. **d** MPP tracking, and PCE distribution of perovskite-organic TSCs based on 30 devices. **e** Long-term photostability of encapsulated tandem devices under continuous one-sun illumination in ambient. The initial efficiency of TSCs with and without TPE-BTA3 are 26.3% and 25.7%, respectively.

Leveraging the optimized PM6:BTP-eC9:TPE-BTA3 bulk-heterojunction with suppressed non-radiative losses and enhanced near-infrared harvesting, we constructed monolithic perovskite-organic TSCs through spectral-complementary integration with a wide bandgap (WBG) perovskite (FA_0.8_Cs_0.2_PbI_1.5_Br_1.5_, 1.85 eV). Figure 4a and Supplementary Fig. 26 demonstrate the tandem structure of ITO/4PADCB/perovskite/C_60_/BCP/Au/MoO_3_/2-PACz/organic layer::/PNDIT-F3N/Ag. Prior to investigating the photovoltaic performance of the tandem device, we systematically optimized the wide-bandgap (WBG) perovskite solar cells as individual sub-cells, achieving a PCE of 18.1% with an *V*_OC_ of 1.37 V, a *J*_SC_ of 16.3 mA cm^−2^, and a FF of 81.2% (Fig. 4b and Table 1). Building upon the optimized WBG PSCs and organic sub-cells, our integrated perovskite-organic TSCs achieved an exceptional PCE of 26.5%, along with an impressive *V*_OC_ of 2.20 V, *J*_SC_ of 14.5 mA cm^−2^, and a FF of 83.1%. The independent certification further confirmed their high photovoltaic performance, with PCEs of 25.83% and 25.14% under reverse and forward scan, respectively (Supplementary Fig. 27). This PCE represents one of the highest certified efficiencies reported to date for perovskite-organic TSCs (Supplementary Fig. 28 and Supplementary Table 9). In addition, Minimal hysteresis is observed between *J*–*V* curves measured under forward and reverse scans (Supplementary Fig. 29). Figure 4c further demonstrates the individual EQE curves of the front and rear sub-cells in the tandem device. The integral *J*_SC_ for the perovskite and organic sub-cells is calculated to be 14.5 and 14.4 mA cm^−2^, respectively, indicating an excellent current matching. The reproducibility of these tandem devices was evaluated by statistical analysis of 30 independent cells. As demonstrated in Fig. 4d, the champion device maintains a stable PCE of 26.3% during 300 s of maximum power point (MPP) tracking under continuous AM1.5 G illumination, with no observed degradation. In addition, these tandem cells present an average PCE of 26.1 ± 0.2% with excellent reproducibility.

**Table 1:** Photovoltaic parameters of individual single-junction WBG PSC, OPV, and their integrated tandem devices measured under AM1.5 G illumination, the average value and standard deviation are calculated from 30 independent devices

Cell type	*V*_OC_ (V)	*J*_SC_ (mA cm^−2^)	FF (%)	PCE_max_/PCE_ave_ (%)
PSC	1.37	16.1	85.7	18.9/18.6 ± 0.1
OSC	0.86	28.9	79.4	19.7/19.4 ± 0.2
Tandem	2.20	14.5	83.1	26.5/26.1 ± 0.2

Benefiting from the excellent photovoltaic performance, we further investigated the properties of the interconnecting recombination layer Au/ MoO_3_/2-PACz. As shown in Supplementary Fig. 30, the average series resistances of OSCs, PSCs, and TSCs are 37.6 ± 4.6, 51.8 ± 4.8, and 91.4 ± 4.6 Ω, respectively. Notably, the series resistance of the tandem device is approximately equal to the sum of those of the two sub-cells, demonstrating the excellent electrical conductivity of the interconnecting layer for efficient carrier recombination. The energy level diagram of the TSC (Supplementary Fig. 31) further reveals a favorable energy level alignment for carrier extraction toward the external electrodes, as well as effective electron–hole recombination at the internal recombination layer. In addition, the phosphate group in 2-PACz tends to anchor onto the MoO_3_ surface, forming a robust Mo–O–P coordinate bond that enhances structural stability. Density functional theory calculations also confirm a strong binding energy of 3.44 eV between MoO_3_ and 2-PACz, as illustrated in Supplementary Fig. 32.

We further investigated the impact of TPE-BTA3 on device stability. As shown in Supplementary Fig. 33, incorporating TPE-BTA3 significantly improves the long-term storage stability of single-junction OSCs. The long-term photostability of encapsulated tandem devices was also monitored under continuous one-sun illumination. As shown in Fig. 4e, the TSCs exhibit a T80 lifetime exceeding 600 h, demonstrating excellent stability. Additionally, thermal stability testing revealed that these devices retained 80% of their initial PCE after 187 hat 85 °C (Supplementary Fig. 34). Notably, in both stability tests, devices based on TPE-BTA3 exhibited slightly better stability than the control devices.

To further verify that the improvement in PCE originates from the AIE effect, we introduced BTA3, a structurally analogous molecule without AIE activity (Supplementary Fig. 35), as a third component into the PM6:BTP-eC9 system for control experiments. As illustrated in Supplementary Fig. 36, the addition of BTA3 did not enhance the photovoltaic performance of either single-junction or tandem devices; instead, it led to a clear decrease in device efficiency. These results strongly confirm that the AIE effect of TPE-BTA3 is responsible for the effectively improved photovoltaic performance. The corresponding photovoltaic parameters of single-junction and tandem devices are summarized in Supplementary Table 10.

## Discussion

In conclusion, we demonstrate a small molecule TPE-BTA3 with AIE properties through rational design, which promotes synergistic photovoltaic performance enhancement in ternary OSCs and perovskite-organic TSCs. This study revealed that TPE-BTA3 can form an alloy acceptor with BTP-eC9 and serve as an energy donor for energy transfer to either PM6 or BTP-eC9. Interestingly, TPE-BTA3 not only preserves a high PLQY after being dispersed into other materials but also mitigates the ACQ effect of BTP-eC9. This leads to a significant boost in the PLQY of the BTP-eC9:TPE-BTA3 alloy-acceptor, accompanied by an increase in the EQE_EL_ and a decrease in the non-radiative recombination energy loss. The GIWAXS test confirmed that TPE-BTA3 could disperse the stacking of BTP-eC9 and inhibit its over-aggregation, benefiting from the improved microstructure, the ternary device exhibits a higher utilization rate of near-infrared photons. As a result, the ternary devices exhibit both improvements in *V*_OC_ and *J*_SC_. Given the excellent photovoltaic performance of the ternary OSCs, we successfully fabricated perovskite-organic TSCs that achieved a surprising PCE of 26.5%, representing one of the highest values reported to date for this type of tandem architecture. We have thus identified a possible approach to apply AIEgen in OSCs and TSCs, explored the logic between the peculiar properties of AIEgen and the reduction of energy loss, and provided insights for the further cross-development of AIEgen with photovoltaics.

## Methods

### Materials

TPE-BTA3 was designed by *Erjun Zhou*’s group, and the synthesis routes are shown in the Supporting Information. PM6, BTP-eC9, and PNDIT-F3N were purchased from HYPER. 2-PACz and 4PADCB (>98%) were purchased from TCI. FAI (99.999%) was purchased from GreatCell Solar Materials. CsI (99.9%) was purchased from Thermo Scientific. PbBr_2_ (99.999%, trace metals basis) was purchased from Energy Chemical. PbI_2_ (99.99%) was purchased from Sigma-Aldrich.

### Fabrication of single-junction OSCs

The single-junction OSCs were fabricated with a conventional structure of ITO/2-PACz/active layer/ PNDIT-F3N/Ag. The ITO-coated glass substrates were sequentially cleaned in detergent-deionized water and ethanol for at least 30 min each at room temperature. 0.3 mg/mL 2-PACz was first spin-casted on top of the ITO substrates at 3000 rpm and then annealed at 100 °C for 5 min. The donor: acceptor: third component (D:A) ratio of 1:1.2:*n* (w/w) for PM6:BTP-eC9:TPE-BTA3 was dissolved in chloroform with PM6 = 7.5 mg/mL, and 10 mg/mL 1,4-Diiodobenzene was added as a solid additive. The active layers were spin-coated at a speed of 2500 rpm, and then with a thermal annealing of 100 °С/10 min. A PNDIT-F3N layer was spin-coated on the top of active layers at 3000 rpm, which was dissolved in methanol at a concentration of 1 mg/mL. Finally, an Ag (120 nm) metal top electrode was thermally evaporated under 4 × 10^−4^ Pa. The device area was 0.08 cm^2^. The active layer thickness is about 120 nm.

### Fabrication of single-junction PSCs

FAI, CsI, PbI_2_, and PbBr_2_ with molar ratios of 0.8:0.2:0.25:0.75, were dissolved in a mixed solvent of DMF and DMSO (4:1 vol%) with a concentration of 1.0 mol/L to form the WBG perovskite precursor. 4PADCB solution (0.5 mg/mL in ethanol) was spin-coated on pre-cleaned ITO and annealed at 100 °C for 10 min. Perovskite precursor was spin-coated on the 4PADCB film at 3000 rpm for 30 s, CB (200 μl) was dropped onto the film during the last 15 s of the spin-coating process. Then the resulting film was annealed at 100 °C for 10 min to form the perovskite film. Subsequently, 20 nm of C_60_, 5 nm of BCP, and 120 nm of Cu were deposited on top via thermal evaporation.

### Fabrication of TSCs

After fabricating the WBG PSCs without a Cu electrode, 0.5 nm-thick Au and 10 nm MoO_3_ film were sequentially deposited via thermal evaporation as recombination layers. Finally, the tandem devices are completed by following the same procedure for fabricating a single junction OSC.

### Photovoltaic characterizations

The *J*–*V* curves of all devices were tested under the illumination of AM 1.5 G (100 mW cm^−2^) by a Keithley 2400 source meter together with a 3 A solar simulator (CME-Sol 8040-3 A, Microenerg Beijing Technology Co., Ltd.). The areas of the solar cells were determined by a shade mask with an aperture area of 6.11 mm^2^. The light intensity was calibrated using a 2 × 2 cm^2^ reference monosilicon cell (Oriel PN 91150 V, Newport, USA), which was itself calibrated by the National Renewable Energy Laboratory. The long-term photostability of the devices was measured under a full-spectrum LED illumination with an intensity equivalent to 1 sun in ambient conditions with humidity of 30–50%. The devices were encapsulated with UV-curable adhesive and slide glass before measurement. Highly sensitive EQE was measured using an integrated system (PECT-600, Enlitech), where the photocurrent was amplified and modulated by a lock-in instrument. EQE_EL_ measurements were performed by applying external voltage/current sources through the devices (ELCT-3010, Enlitech). The particle size was tested by Zetasizer Nano ZS-90 Laser particle sizer. For the EQE measurement of the perovskite and organic sub-cells, bias illumination from high-brightness LEDs with emission peaks at 550 nm and 780 nm was used to saturate the other junctions.

### Other characterizations

UV-vis spectra were tested by UV-3600i PLUS (Shimadzu Corporation). The film specimens were spin-coated on a quartz substrate. The concentration of solution specimens was 0.01 mg mL^−1^. Cyclic voltammetry (CV) measurements were carried out using an electrochemical workstation, equipped with a standard three-electrode configuration. Typically, a three-electrode cell equipped with a Pt plate coated with a thin film as a working electrode, an Ag/AgCl (0.01 M in anhydrous acetonitrile) reference electrode, and a Pt wire counter electrode was employed. The measurements were done in anhydrous acetonitrile with tetrabutylammonium hexafluorophosphate (0.1 M) as the supporting electrolyte under an argon atmosphere at a scan rate of 100 mV/s. The potential of the Ag/AgCl reference electrode was internally calibrated by using the ferrocene/ferrocenium redox couple (Fc/Fc^+^). PL, TRPL, and PLQY were tested by the Edinburgh Fluorescence Spectrometer (FLS 1000), and the film specimens were spin-coated on a quartz substrate. The contact angle was tested by JY-82C (Chengde Dingsheng). Atomic force microscopy (AFM) was tested by Bruker Dimension Icon in tapping mode. The active layers were spin-coated onto the ITO substrate. GIWAXS was tested by the XEUSS SAXS/WAXS equipment. The data were obtained with an area Pilatus 100k detector with a resolution of 195 × 487 pixels (0.172 mm × 0.172 mm). The X-ray wavelength was 1.54 Å, and the incidence angle was 0.2°. TA spectroscopy was tested by a regenerative amplified Ti: sapphire laser system (Coherent) as the laser source and EOS spectrometer (Ultrafast Systems LLC) as the spectrometer, with a pump beam excitation intensity at 10 μJ cm^−2^. The active layers were spin-coated onto the Si substrate. All the samples were prepared with the optimized device fabrication conditions.

### Reporting summary

Further information on research design is available in the Nature Portfolio Reporting Summary linked to this article.

## Supplementary information

Supplementary information

Reporting Summary

Transparent Peer Review file

## Data Availability

The data that support this study are available in the Supplementary Information/Source Data file (Figshare 10.6084/m9.figshare.31932261).

## References

[1] Zhang, G. et al. Nonfullerene acceptor molecules for bulk heterojunction organic solar cells. *Chem. Rev.* **118**, 3447–3507 (2018). 29557657 10.1021/acs.chemrev.7b00535

[2] Wang, D. et al. High-performance semitransparent organic solar cells with excellent infrared reflection and see-through functions. *Adv. Mater.* **32**, 2001621 (2020). 10.1002/adma.202001621 32613625

[3] Zeng, R. et al. All-polymer organic solar cells with nano-to-micron hierarchical morphology and large light receiving angle. *Nat. Commun.* **14**, 4148 (2023). 37438377 10.1038/s41467-023-39832-4 PMC10338490

[4] Ren, J. et al. Manipulating aggregation kinetics toward efficient all-printed organic solar cells. *Adv. Mater.* **37**, 2418353 (2025). 10.1002/adma.202418353 39906018

[5] Yuan, J. et al. Single-junction organic solar cell with over 15% efficiency using fused-ring acceptor with electron-deficient core. *Joule* **3**, 1140–1151 (2019).

[6] Zhang, M. et al. Efficient and stable high-entropy organic photovoltaics. *Joule*10.1016/j.joule.2025.101851 (2025).

[7] Li, C. et al. Non-fullerene acceptors with high crystallinity and photoluminescence quantum yield enable >20% efficiency organic solar cells. *Nat. Mater.* **24**, 433–443 (2025). 39880932 10.1038/s41563-024-02087-5

[8] Wu, S. et al. Redox mediator-stabilized wide-bandgap perovskites for monolithic perovskite-organic tandem solar cells. *Nat. Energy* **9**, 411–421 (2024).

[9] Zhang, Z. et al. Suppression of phase segregation in wide-bandgap perovskites with thiocyanate ions for perovskite/organic tandems with 25.06% efficiency. *Nat. Energy* **9**, 592–601 (2024).

[10] Brinkmann, K. O. et al. Perovskite–organic tandem solar cells with indium oxide interconnect. *Nature* **604**, 280–286 (2022). 35418631 10.1038/s41586-022-04455-0

[11] Yao, Q. et al. Dual sub-cells modification enables high-efficiency n–i–p type monolithic perovskite/organic tandem solar cells. *Adv. Funct. Mater.* **33**, 2212599 (2023).

[12] Guo, X. et al. Stabilizing efficient wide-bandgap perovskite in perovskite-organic tandem solar cells. *Joule* **8**, 2554–2569 (2024).

[13] Han, Y. et al. Inorganic perovskite/organic tandem solar cells with 25.1% certified efficiency via bottom contact modulation. *Nat. Energy*10.1038/s41560-025-01742-8 (2025).

[14] Jiang, X. et al. Isomeric diammonium passivation for perovskite–organic tandem solar cells. *Nature* **635**, 860–866 (2024). 39401516 10.1038/s41586-024-08160-y

[15] Chen, X. et al. Efficient perovskite/organic tandem photovoltaic devices and large-area modules featuring thick-film organic solar cells. *Adv. Mater.*10.1002/adma.202500190 (2025). 10.1002/adma.202500190 40207610

[16] Xie, G. et al. Crystallization thermodynamics regulation of 1.85 eV wide-bandgap perovskite for efficient and stable perovskite-organic tandem photovoltaics. *Angew. Chem. Int. Ed*. 10.1002/anie.202501764 (2025). 10.1002/anie.202501764 39927523

[17] Jia, Z. et al. Efficient near-infrared harvesting in perovskite–organic tandem solar cells. *Nature* **643**, 104–110 (2025). 40562931 10.1038/s41586-025-09181-x

[18] Liu, Z. et al. All-perovskite tandem solar cells achieving >29% efficiency with improved (100) orientation in wide-bandgap perovskites. *Nat. Mater.* **24**, 252–259 (2025). 39794635 10.1038/s41563-024-02073-x

[19] Shockley, W. & Queisser, H. J. Detailed Balance Limit of Efficiency of p-n junction solar cells. *J. Appl. Phys.* **32**, 510–519 (1961).

[20] Rühle, S. Tabulated values of the Shockley–Queisser limit for single junction solar cells. *Sol. Energy* **130**, 139–147 (2016).

[21] Rau, U. Reciprocity relation between photovoltaic quantum efficiency and electroluminescent emission of solar cells. *Phys. Rev. B* **76**, 085303 (2007).

[22] Geffroy, B., le Roy, P. & Prat, C. Organic light-emitting diode (OLED) technology: materials, devices and display technologies. *Polym. Int.* **55**, 572–582 (2006).

[23] He, D., Zhao, F., Wang, C. & Lin, Y. Non-radiative recombination energy losses in non-fullerene organic solar cells. *Adv. Funct. Mater.* **32**, 2111855 (2022).

[24] Qian, D. et al. Design rules for minimizing voltage losses in high-efficiency organic solar cells. *Nat. Mater.* **17**, 703–709 (2018). 30013057 10.1038/s41563-018-0128-z

[25] Gan, Z. et al. Distance makes a difference in crystalline photoluminescence. *Nat. Commun.* **11**, 5572 (2020). 33149132 10.1038/s41467-020-19377-6 PMC7643180

[26] Chen, X. et al. A unified description of non-radiative voltage losses in organic solar cells. *Nat. Energy* **6**, 799–806 (2021).

[27] Zhang, G. et al. Delocalization of exciton and electron wavefunction in non-fullerene acceptor molecules enables efficient organic solar cells. *Nat. Commun.* **11**, 3943 (2020). 32770068 10.1038/s41467-020-17867-1 PMC7414148

[28] Qin, Y. et al. Reduced nonradiative energy loss caused by aggregation of nonfullerene acceptor in organic solar cells. *Adv. Energy Mater.* **9**, 1901823 (2019).

[29] Liu, Q. et al. Synergistically minimized nonradiative energy loss and optimized morphology achieved via the incorporation of small molecule donor in 17.7% efficiency ternary polymer solar cells. *Nano Energy* **85**, 105963 (2021).

[30] Mei, J., Leung, N. L. C., Kwok, R. T. K., Lam, J. W. Y. & Tang, B. Z. Aggregation-induced emission: together we shine, united we soar!. *Chem. Rev.* **115**, 11718–11940 (2015). 26492387 10.1021/acs.chemrev.5b00263

[31] Xie, H. et al. Mechanochemical fabrication of full-color luminescent materials from aggregation-induced emission prefluorophores for information storage and encryption. *J. Am. Chem. Soc.* **146**, 18350–18359 (2024). 38937461 10.1021/jacs.4c02954 PMC11240258

[32] Liu, S. et al. Diketopyrrolopyrrole-based oligomers accessed via sequential CH activated coupling for fullerene-free organic photovoltaics. *Dyes Pigments* **134**, 139–147 (2016).

[33] Raynor, A. M. et al. Significant improvement of optoelectronic and photovoltaic properties by incorporating thiophene in a solution-processable D–A–D modular chromophore. *Molecules* **20**, 21787–21801 (2015). 26690103 10.3390/molecules201219798 PMC6332373

[34] Rananaware, A. et al. A four-directional non-fullerene acceptor based on tetraphenylethylene and diketopyrrolopyrrole functionalities for efficient photovoltaic devices with a high open-circuit voltage of 1.18 V. *Chem. Commun.* **52**, 8522–8525 (2016). 10.1039/c6cc03730e 27263442

[35] Liu, Y. et al. A tetraphenylethylene core-based 3D structure small molecular acceptor enabling efficient non-fullerene organic solar cells. *Adv. Mater.* **27**, 1015–1020 (2015). 25429918 10.1002/adma.201404152

[36] Liu, Y. et al. Efficient non-fullerene polymer solar cells enabled by tetrahedron-shaped core based 3D-structure small-molecular electron acceptors. *J. Mater. Chem. A* **3**, 13632–13636 (2015).

[37] Lin, H. et al. Reduced intramolecular twisting improves the performance of 3D molecular acceptors in non-fullerene organic solar cells. *Adv. Mater.* **28**, 8546–8551 (2016). 27501996 10.1002/adma.201600997

[38] Adil, M. A. et al. Regulating the phase separation of ternary organic solar cells via 3D architectured AIE molecules. *Nano Energy* **68**, 104271 (2020).

[39] An, N. et al. Solution-processed organic solar cells with high open-circuit voltage of 1.3 V and low non-radiative voltage loss of 0.16 V. *Adv. Mater.* **32**, 2002122 (2020). 10.1002/adma.202002122 32844465

[40] Xu, Y. et al. A new conjugated polymer that enables the integration of photovoltaic and light-emitting functions in one device. *Adv. Mater.* **33**, 2101090 (2021). 10.1002/adma.202101090 33899285

[41] Cui, Y. et al. Single-junction organic photovoltaic cells with approaching 18% efficiency. *Adv. Mater.* **32**, 1908205 (2020). 10.1002/adma.201908205 32227399

[42] Lu, T. & Chen, F. Multiwfn: a multifunctional wavefunction analyzer. *J. Comput. Chem.* **33**, 580–592 (2012). 22162017 10.1002/jcc.22885

[43] Liao, X. et al. Inhibiting excessive molecular aggregation to achieve highly efficient and stabilized organic solar cells by introducing a star-shaped nitrogen heterocyclic-ring acceptor. *Energy Environ. Sci.* **15**, 384–394 (2022).

[44] Yu, R. et al. Improved charge transport and reduced nonradiative energy loss enable over 16% efficiency in ternary polymer solar cells. *Adv. Mater.* **31**, 1902302 (2019). 10.1002/adma.201902302 31294900

[45] Li, X. et al. Benzotriazole-based 3D four-arm small molecules enable 19.1% efficiency for PM6: Y6-based ternary organic solar cells. *Angew. Chem. Int. Ed.* **62**, e202306847 (2023). 10.1002/anie.202306847 37565778

[46] Zeng, Y. et al. Exploring the charge dynamics and energy loss in ternary organic solar cells with a fill factor exceeding 80%. *Adv. Energy Mater.* **11**, 2101338 (2021).

