# Supplementary material for: Aggregation-induced emission luminogen in ternary organic bulk-heterojunction for efficient perovskite-organic tandem solar cells
Source: Nat Commun. 2026 May 30;17:7019. doi: 10.1038/s41467-026-73743-4 (PMC13392458; doi:10.1038/s41467-026-73743-4)
Supplement: Supplementary file 1 — Supplementary information [file 41467_2026_73743_MOESM1_ESM.pdf]

Supplementary information for

Aggregation-Induced Emission Luminogen in Ternary Organic Bulk-heterojunction for efficient Perovskite-Organic Tandem Solar Cells

Xiangyu Li[1,2,3], Mengzhen Du[2], Zongtao Wang[3], Qiang Guo[3], Tangyue Xue[3], Helin Wang[4], Jinglin Sun[1], Tingting Dai[2], Chenkai Sun[5], Zhi Zheng[6], Yanming Sun[7], Xing Feng[8], Hui Huang[9], Zhibin Yang[1,*], Erjun Zhou[2,*]

[1] School of Chemistry and Chemical Engineering, Shanghai Jiao Tong University, Shanghai 200240, China

[2] College of Biological and Chemical Engineering, Jiaxing University, Jiaxing, Zhejiang 314001, China

[3] Henan Institute of Advanced Technology, Zhengzhou University, Zhengzhou 450003, China.

[4] National Center for Nanoscience and Technology, Beijing 100190, China.

[5] College of Chemistry, and Green Catalysis Center, Zhengzhou University, Zhengzhou 450001, China.

[6] College of Chemical and Materials Engineering, Xuchang University, Xuchang, Henan 461000, China

[7] School of Chemistry, Beihang University, Beijing 100191, China.

[8] School of Material and Energy, Guangdong University of Technology, Guangzhou, 510006, China.

[9] College of Materials Science and Opto-Electronic Technology, University of Chinese Academy of Sciences, Beijing 100049, China.

** Corresponding author. E-mail: zhibinyang@sjtu.edu.cn (Z.Y.); zhouej@nanoctr.cn (E.Z.)*

Supplementary Notes 1-3

Supplementary Figures 1-36

Supplementary Tables 1-10

Supplementary Note 1:

The synthesis of TPE-BTA: A mixture of 7-bromo-2-octyl-2H-Benzotriazole-4-carboxaldehyde (0.40 g, 1.2 mmol), 1,1,2,2-tetrakis(4-(4,4,5,5-tetramethyl-1,3,2-dioxaborolan-2-yl)phenyl)ethene (0.2 g, 0.24 mmol), and Tetrakis(triphenylphosphine)palladium (70 mg, 0.06 mmol) was added a degassed mixture of THF (20 mL) and 2M potassium carbonate aqueous solution (4 mL). Then, the mixture was stirred at 70 °C for 48 h under an argon atmosphere. After being cooled to room temperature, the water was added and the mixture was extracted with chloroform. The organic layers were washed with brine and dried over magnesium sulfate. The solvent was removed under reduced pressure, and the residue was purified by silica gel column chromatography with dichloromethane:ethyl acetate (100:1, v/v) as eluent to afford a yellow solid (75%, 0.25 g, 0.18 mmol).

The synthesis of TPE-BTA3:The TPE-BTA (0.2 g, 0.15 mmol), 2-(3-ethyl-4-oxo-thiazolidin-2-ylidene)- malononitrile (0.17 g, 0.88 mmol), chloroform (20 mL), and Triethylamine (0.12 mL) were added to a two-necked round-bottom flask. Then, the mixture was stirred at 70 °C for 24 h under an argon atmosphere. After the reaction was completed and cooled to room temperature, the methanol (30 mL) was stirred for 10 minutes and filtered. The residue was purified by silica gel column chromatography with dichloromethane:ethyl acetate (200:1, v/v) as eluent to yield a red solid (61%, 0.18 g, 0.090 mmol).^{1}H NMR (400 MHz, $CDCl_3$) δ 8.35 (d, J = 3.9 Hz, 4H), 8.03 (d, J = 8.2 Hz, 8H), 7.69 (dd, J = 7.8 Hz, 4H), 7.59 (dd, J = 8.0 Hz, 4H), 7.37 (d, J = 8.1 Hz, 8H), 4.77 (t, J = 7.3 Hz, 8H), 4.34 (dd, J = 7.2 Hz, 8H), 2.18 (t, J = 7.1 Hz, 8H), 1.44-1.39 (m, 12H), 1.24 (d, J = 11.4 Hz, 40H), 0.84 (s, 12H). ^{13}C NMR (101 MHz, $CDCl_3$) δ 165.69, 165.52, 143.37, 142.17, 141.63, 140.36, 133.51, 131.14, 129.93, 127.32, 123.00, 120.90, 117.34, 112.11, 111.30, 56.20, 54.47, 39.53, 30.65, 28.78, 28.03, 27.83, 25.50, 21.56, 13.15, 13.04.

Supplementary Note 2:

Detailed energy loss of OSCs.

$$\begin{aligned} E_{\text{loss}} &= E_{\text{g}} - qV_{\text{OC}} \\ &= \left(E_{\text{g}} - qV_{\text{OC}}^{\text{SQ}}\right) + \left(qV_{\text{OC}}^{\text{SQ}} - qV_{\text{OC}}^{\text{rad}}\right) + \left(qV_{\text{OC}}^{\text{rad}} - qV_{\text{OC}}\right) \\ &= \left(E_{\text{g}} - qV_{\text{OC}}^{\text{SQ}}\right) + q\Delta V_{\text{OC}}^{\text{rad,belowgap}} + q\Delta V_{\text{OC}}^{\text{non-rad}} \\ &= \Delta E_1 + \Delta E_2 + \Delta E_3 \end{aligned}$$

Supplementary Note 3:

The determination of ΔE_3 relies on the measurement of the EQE_{EL}, which requires precise calibration of the optical system before measurement. Specifically, the calibration procedure involves placing a standard halogen lamp with a known spectral irradiance at the same position within the integrating sphere as the device under test. The emission spectrum of the standard lamp is recorded by the spectrometer, enabling the derivation of the system's spectral responsivity function. Subsequently, the OSC is driven by a Keithley source measure unit at a constant injected current. The electroluminescence spectrum is collected using the integrating sphere and recorded as a raw signal. This raw signal is then converted to absolute spectral radiance based on the pre-determined responsivity function. The total emitted photon flux is obtained by numerically integrating the spectral radiance across the entire emission spectrum. Finally, EQE_{EL} is calculated as the ratio of the emitted photon flux to the injected electron flux.

Regarding the selection of the injection current range, all measurements were performed at low current densities (typically between 0.1 and several tens of mA/cm^2) to minimize artifacts such as Joule heating, series resistance effects, and device degradation. It is necessary to ensure that the EQE_{EL} values measured at different currents remain constant (plateau region), indicating that measurements are performed within the stable region of the recombination mechanism.

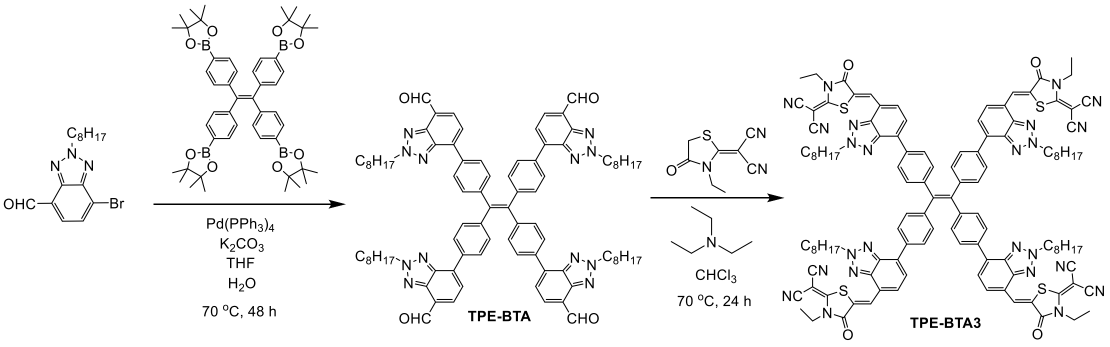

Supplementary Figure 1. The synthetic route of TPE-BTA3.

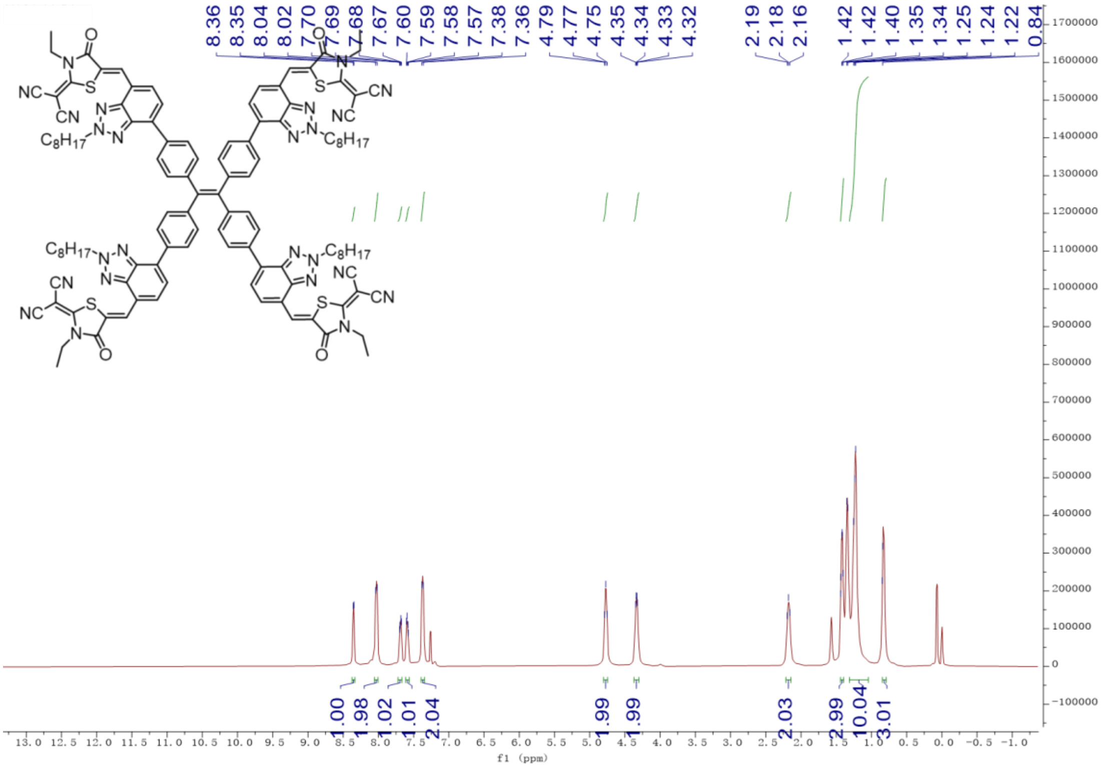

Supplementary Figure 2. ^1H NMR spectrum of TPE-BTA3 in $CDCl_3$.

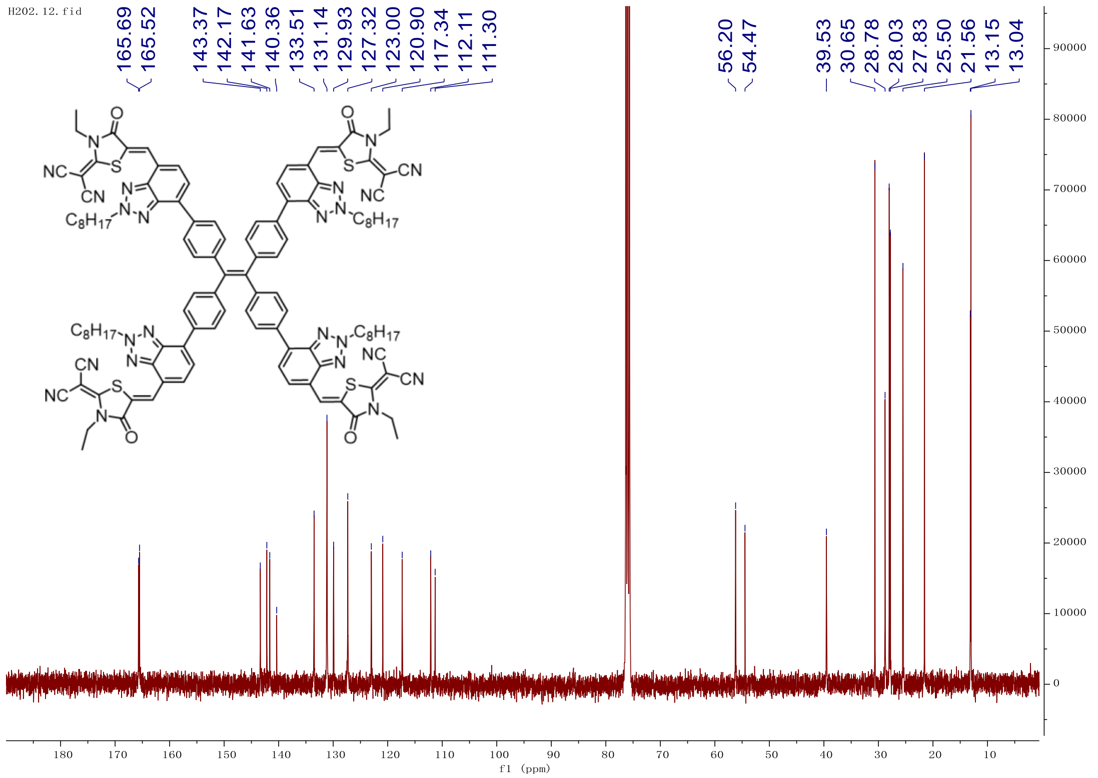

Supplementary Figure 3. ^{13}C NMR spectrum of TPE-BTA3 in $CDCl_3$.

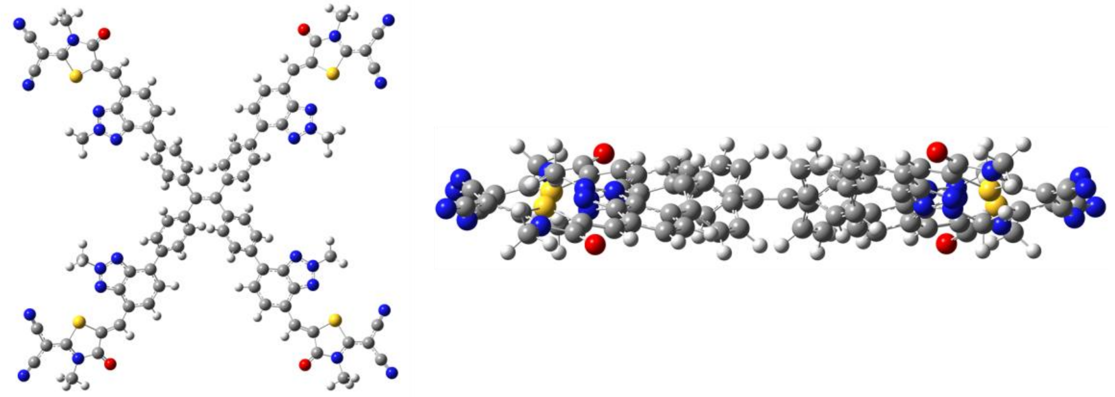

Supplementary Figure 4. Top view and side view of optimized geometries for TPE-BTA3.

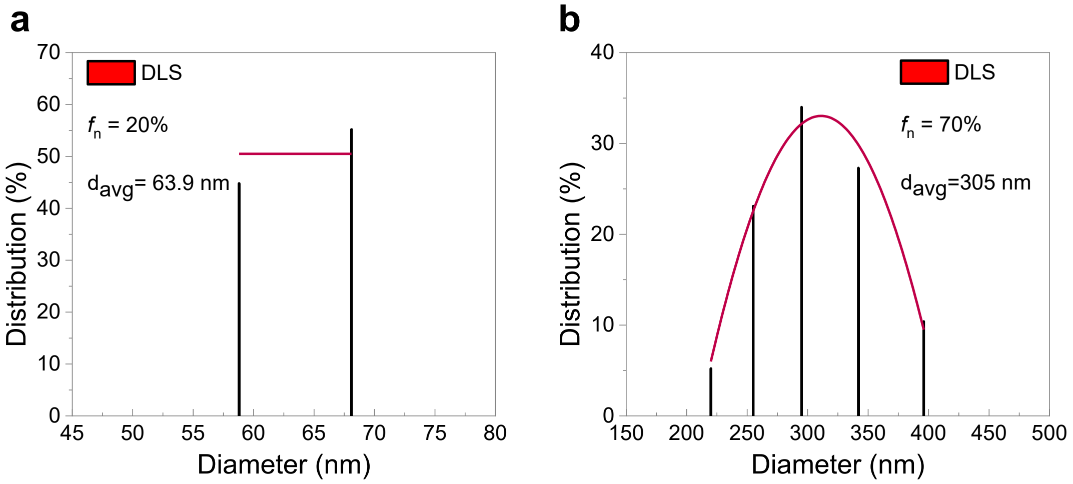

Supplementary Figure 5. The particle size of TPE-BTA3 under different f_n, f_n = 20% (a), f_n = 70% (b) .

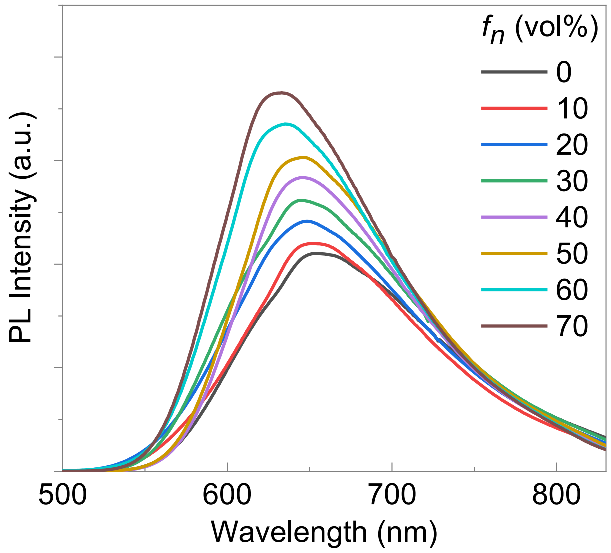

Supplementary Figure 6. PL spectra at varying solvent volume fractions measured with an OD 1.0 filter.

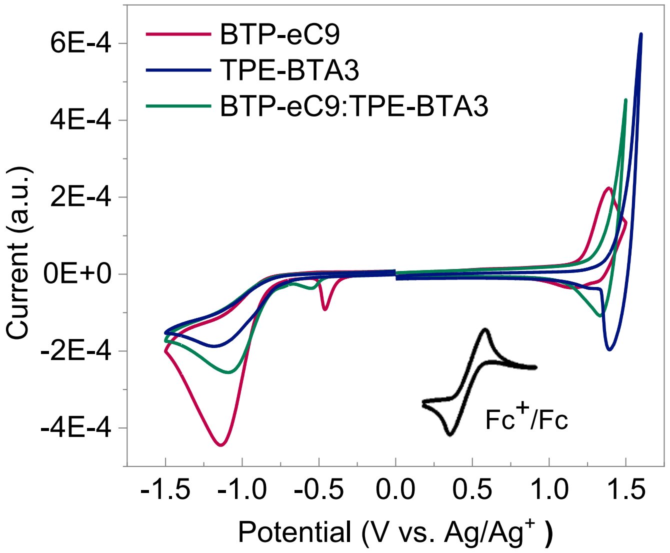

Supplementary Figure 7. Cyclic voltammograms of BTP-eC9, TPE-BTA3, and BTP-eC9:TPE-BTA3 with Ag/AgCl as a reference electrode.

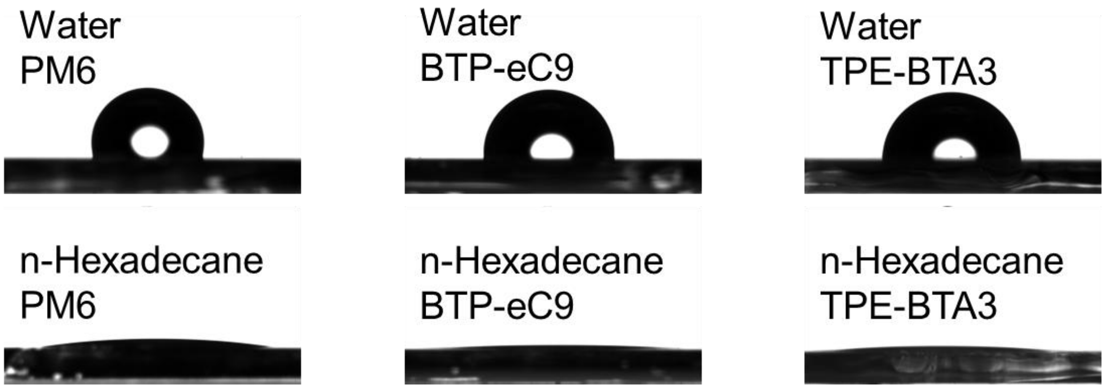

Supplementary Figure 8. Water and n-hexadecane contact angle for PM6, BTP-eC9, and TPE-BTA3.

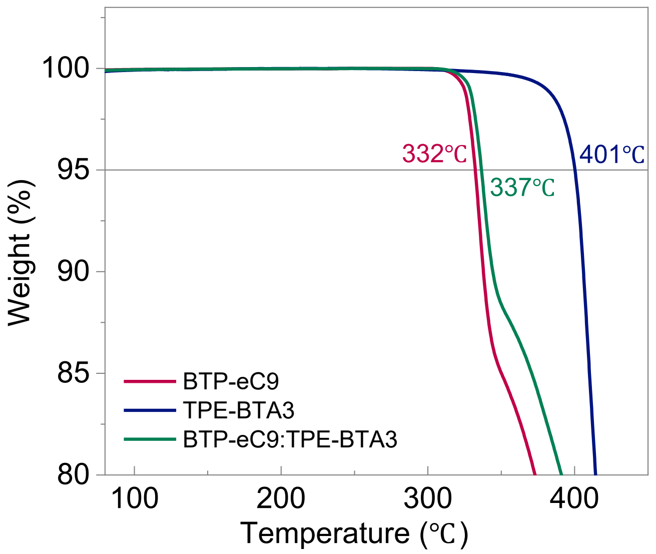

Supplementary Figure 9. Thermogravimetric analysis of pure BTP-eC9, pure TPE-BTA3, and BTP-eC9:TPE-BTA3 blend (10:1 weight ratio).

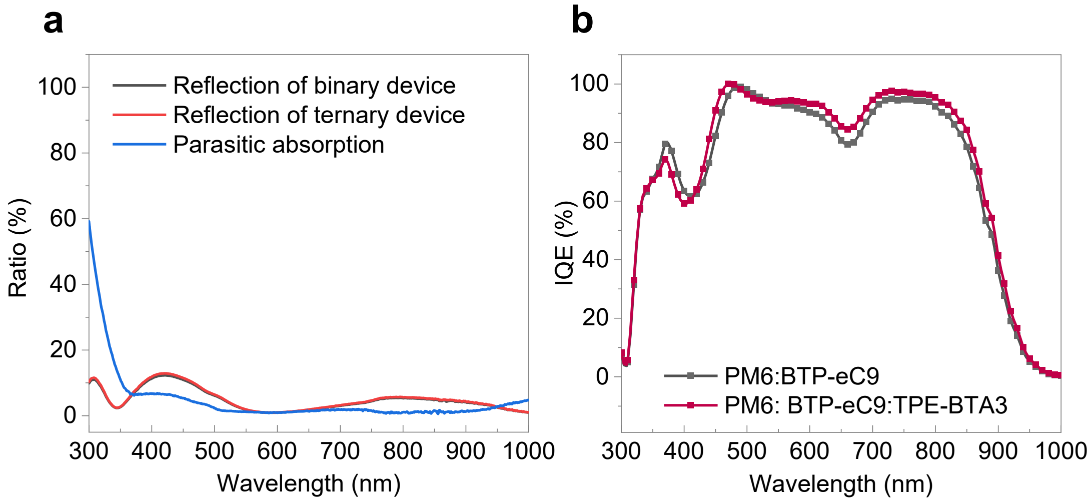

Supplementary Figure 10 a, Reflection of binary and ternary OPV devices, parasitic absorption of the 2-PACz/ITO substrate. b, IQE curves of the binary and ternary OPV devices.

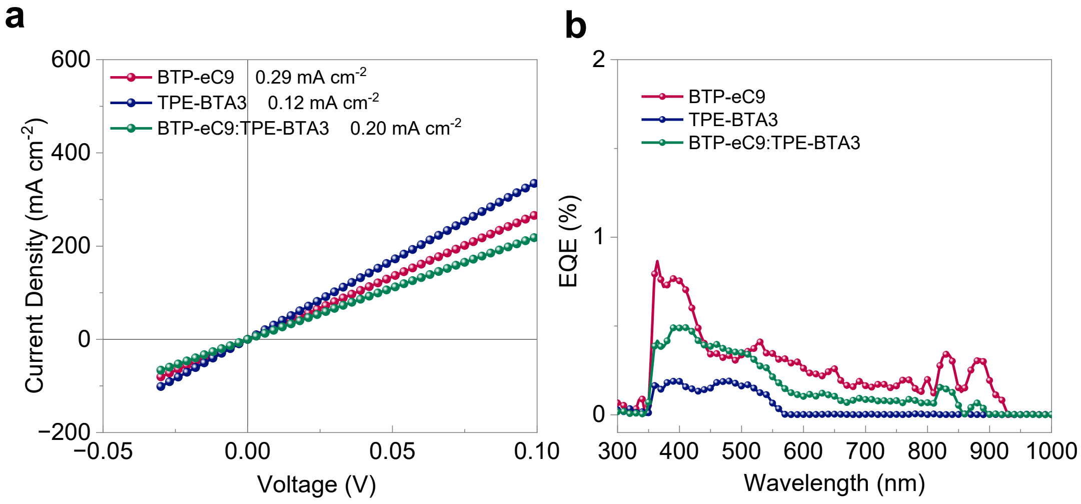

Supplementary Figure 11. a, J-V curves of devices with BTP-eC9, TPE-BTA3, and BTP-eC9:TPE-BTA3 blend films as the active layers. b, EQE curves of devices with BTP-eC9, TPE-BTA3, and BTP-eC9:TPE-BTA3 blend films as the active layers.

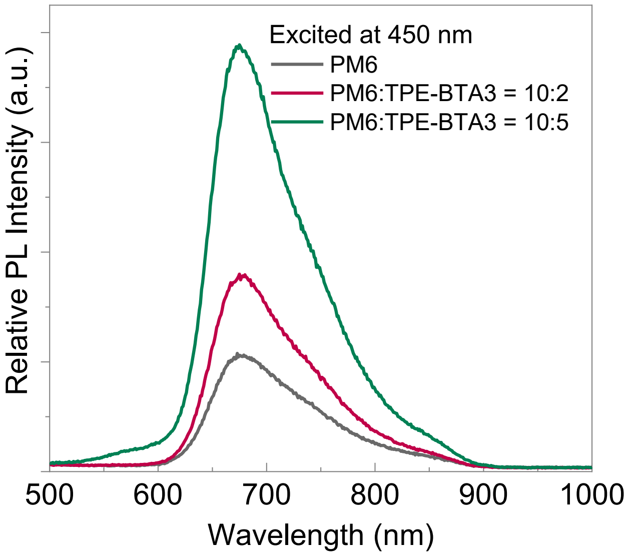

Supplementary Figure 12. PL spectra of pure films and blend films excited at 450 nm.

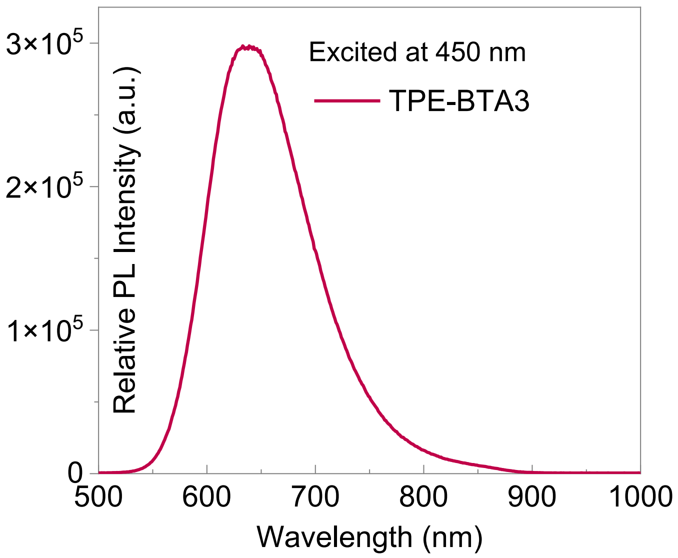

Supplementary Figure 13. PL spectra of TPE-BTA3.

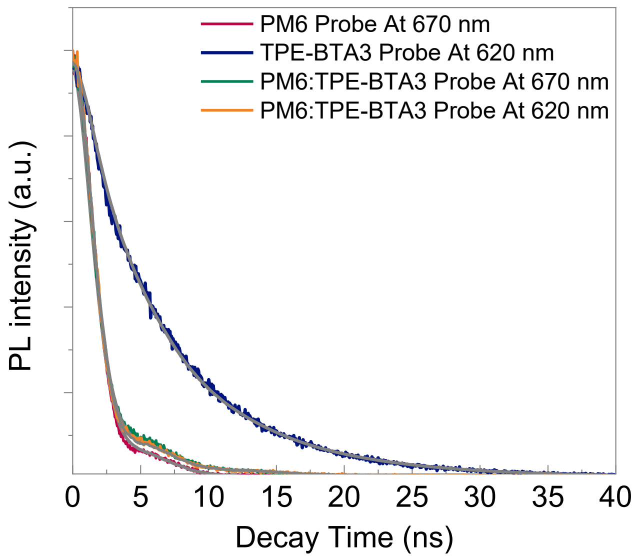

Supplementary Figure 14. TRPL decay curves and fitted curves (gray) of pure films and blend films probed at different wavelengths.

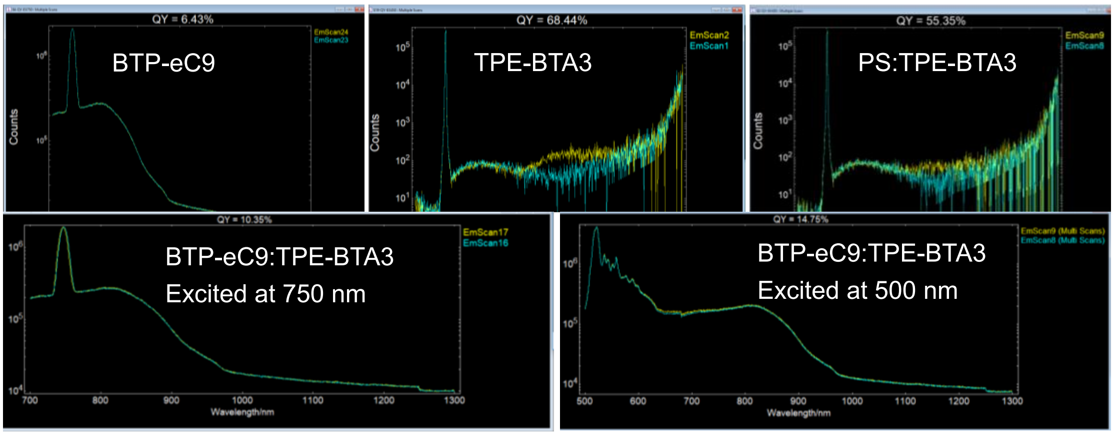

Supplementary Figure 15. PLQY test results of BTP-eC9, TPE-BTA3 pure films, and PS:TPE-BTA3, BTP-eC9:TPE-BTA(excited at 750nm), BTP-eC9:TPE-BTA3(excited at 500 nm) blend films.

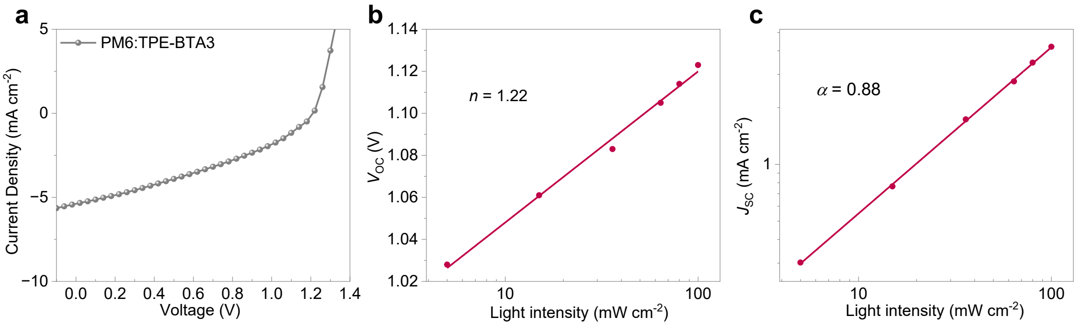

Supplementary Figure 16. J-V curves of PM6:TPE-BTA3 based OSC (a), light intensity dependence of V_{OC} for PM6:TPE-BTA3 device (b), and light intensity dependence of J_{SC} for PM6:TPE-BTA3 device (c).

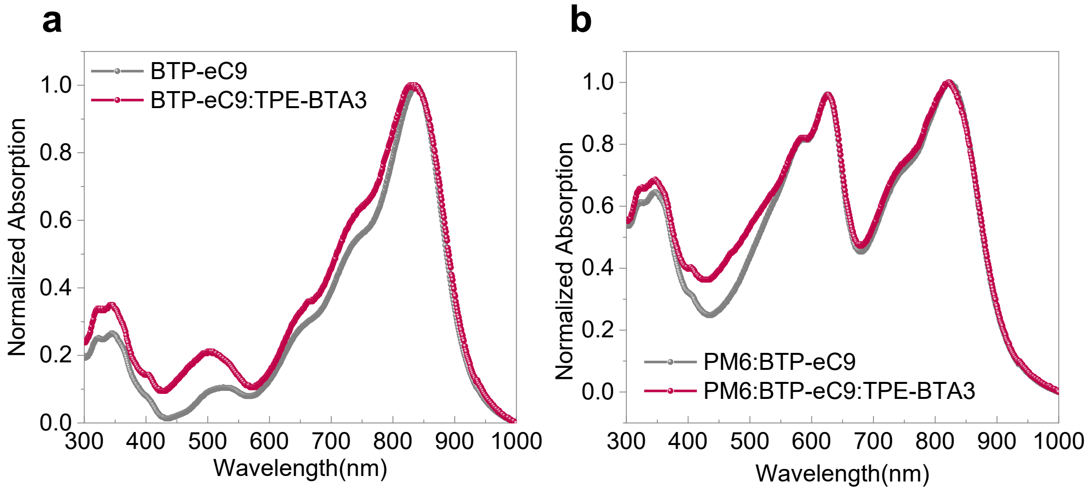

Supplementary Figure 17. The absorption curves of BTP-eC9, BTP-eC9:TPE-BTA3

(a), and PM6:BTP-eC9, PM6:BTP-eC9:TPE-BTA3 (b) films.

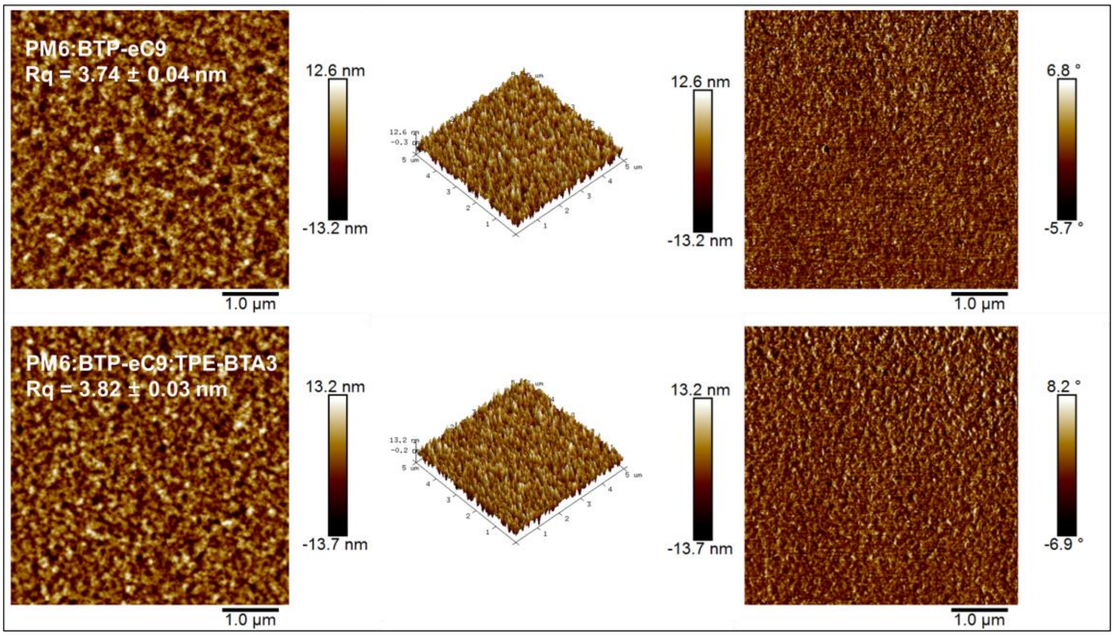

Supplementary Figure 18. AFM images of binary and ternary films include height images, 3D height images, and phase images (the Rq was calculated from 3 independent samples).

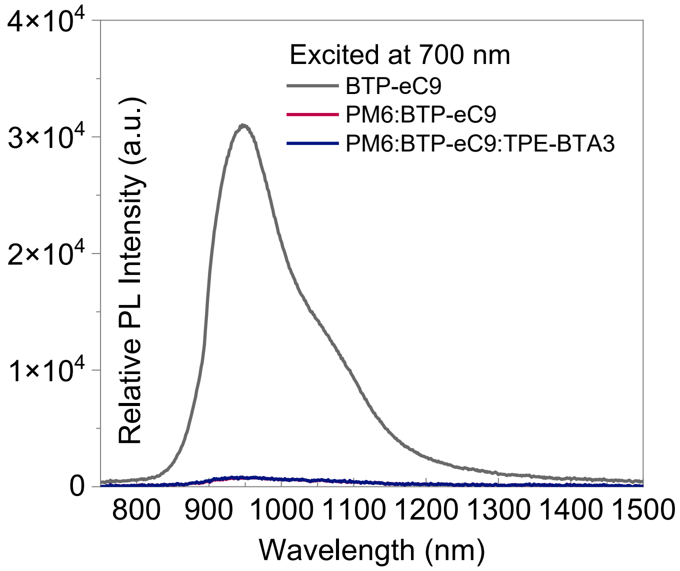

Supplementary Figure 19. PL spectra of pure film and blend films.

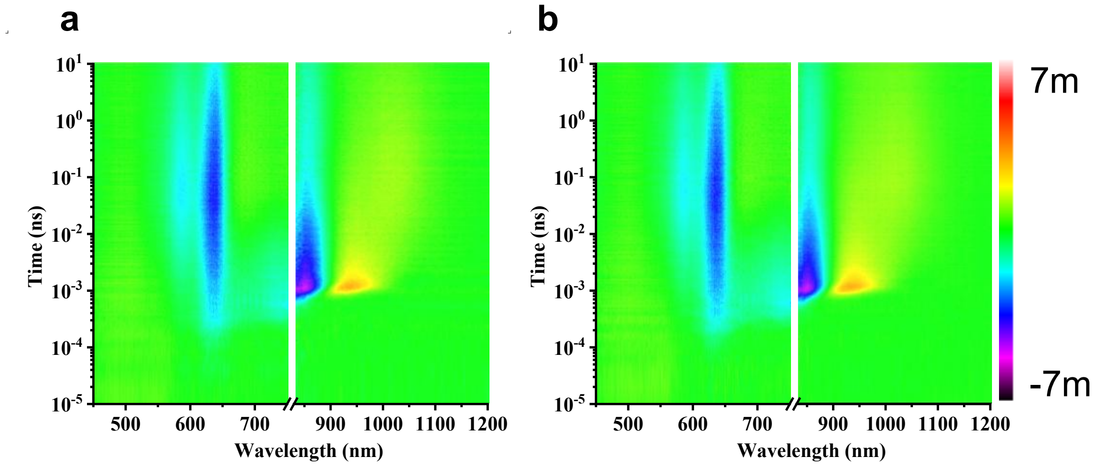

Supplementary Figure 20. Color plot of TA spectra of binary (a) and ternary (b) films under 750 nm excitation.

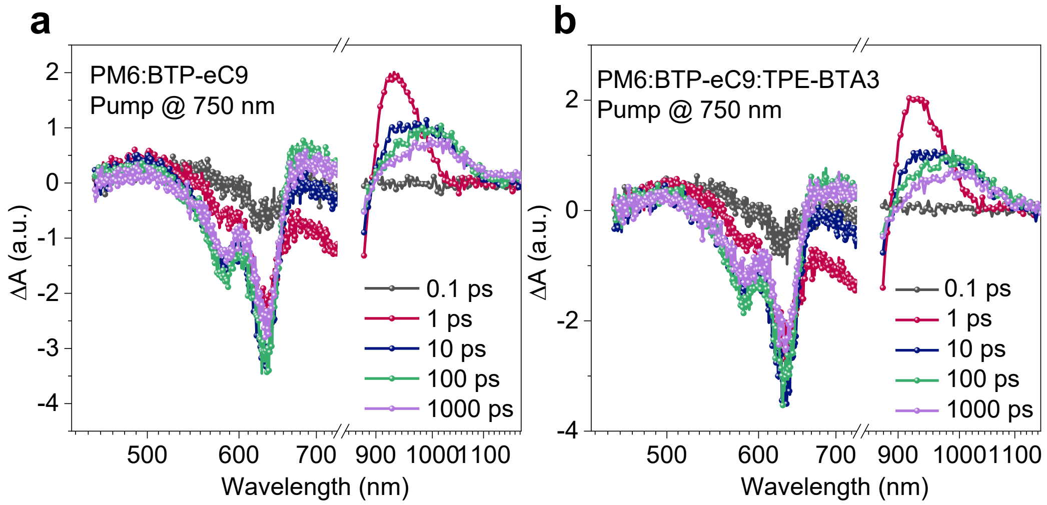

Supplementary Figure 21. TA spectra recorded at different time delays of PM6:BTP-eC9 (a) and PM6:BTP-eC9:BTP-eC9 (b).

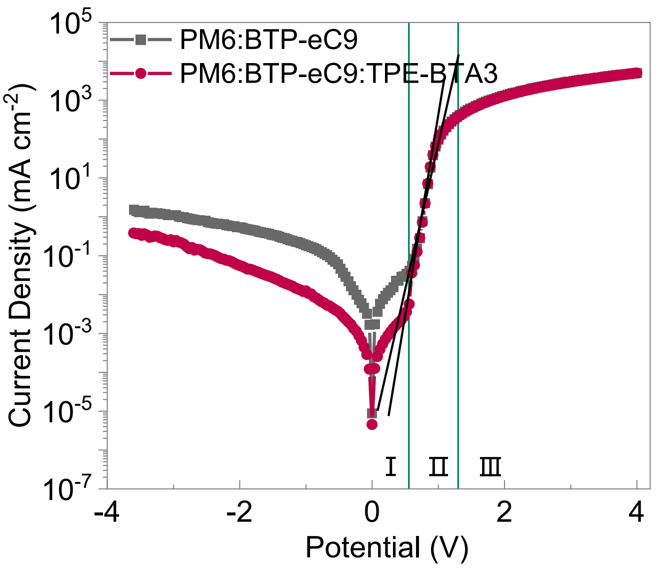

Supplementary Figure 22. *J-V* curves under the dark state.

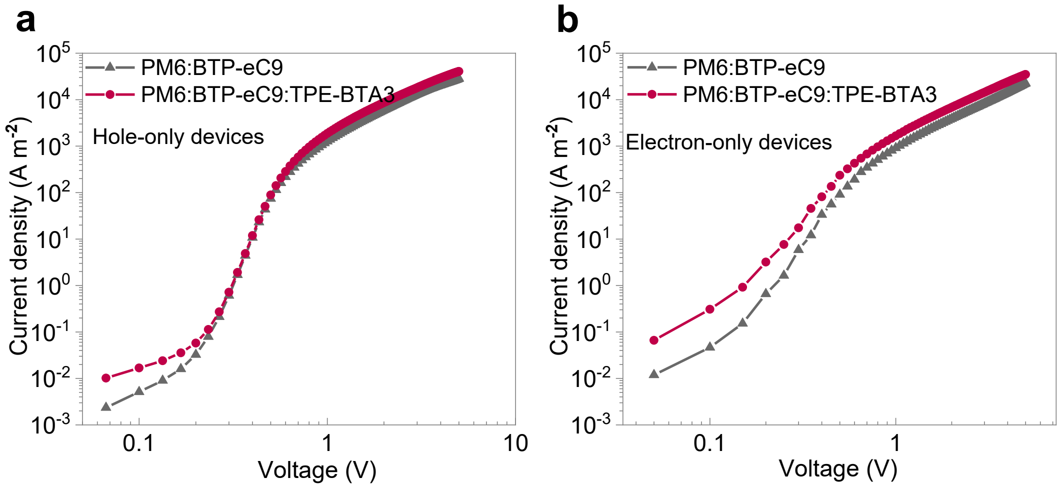

Supplementary Figure 23. *J−V* curves of the hole-only (a) and electron-only (b) devices.

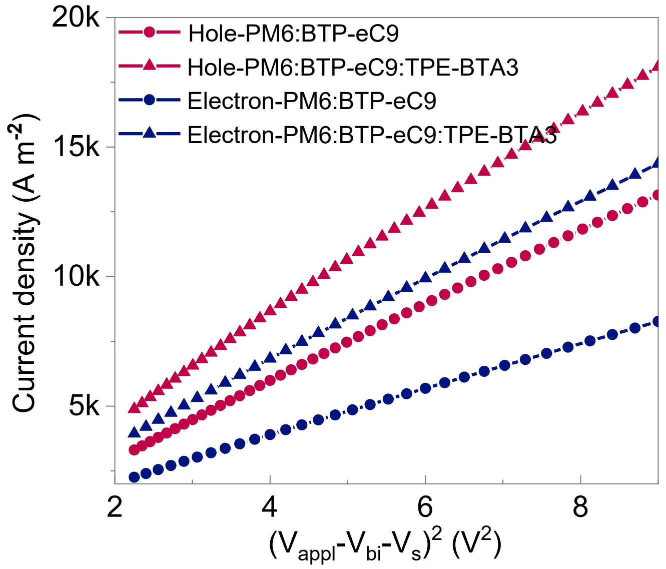

Supplementary Figure 24. Corresponding fitting curves of SCLC.

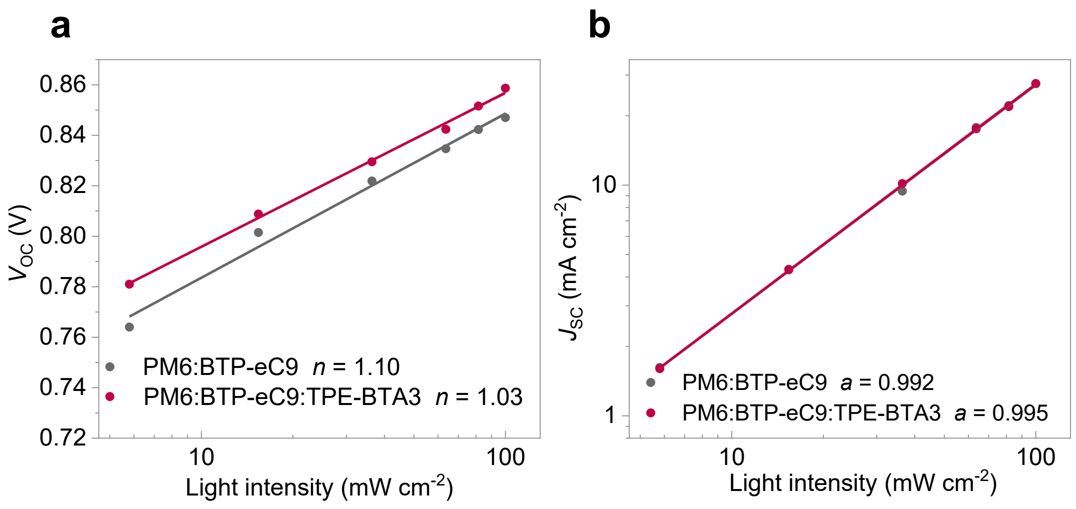

Supplementary Figure 25. Light intensity dependence of V_{OC} (a) and J_{SC} (b).

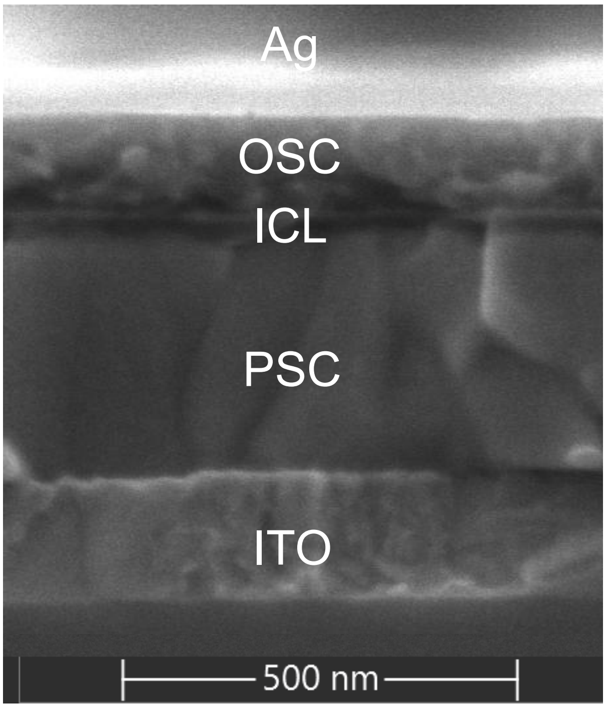

Supplementary Figure 26. SEM image of perovskite/organic TSC.

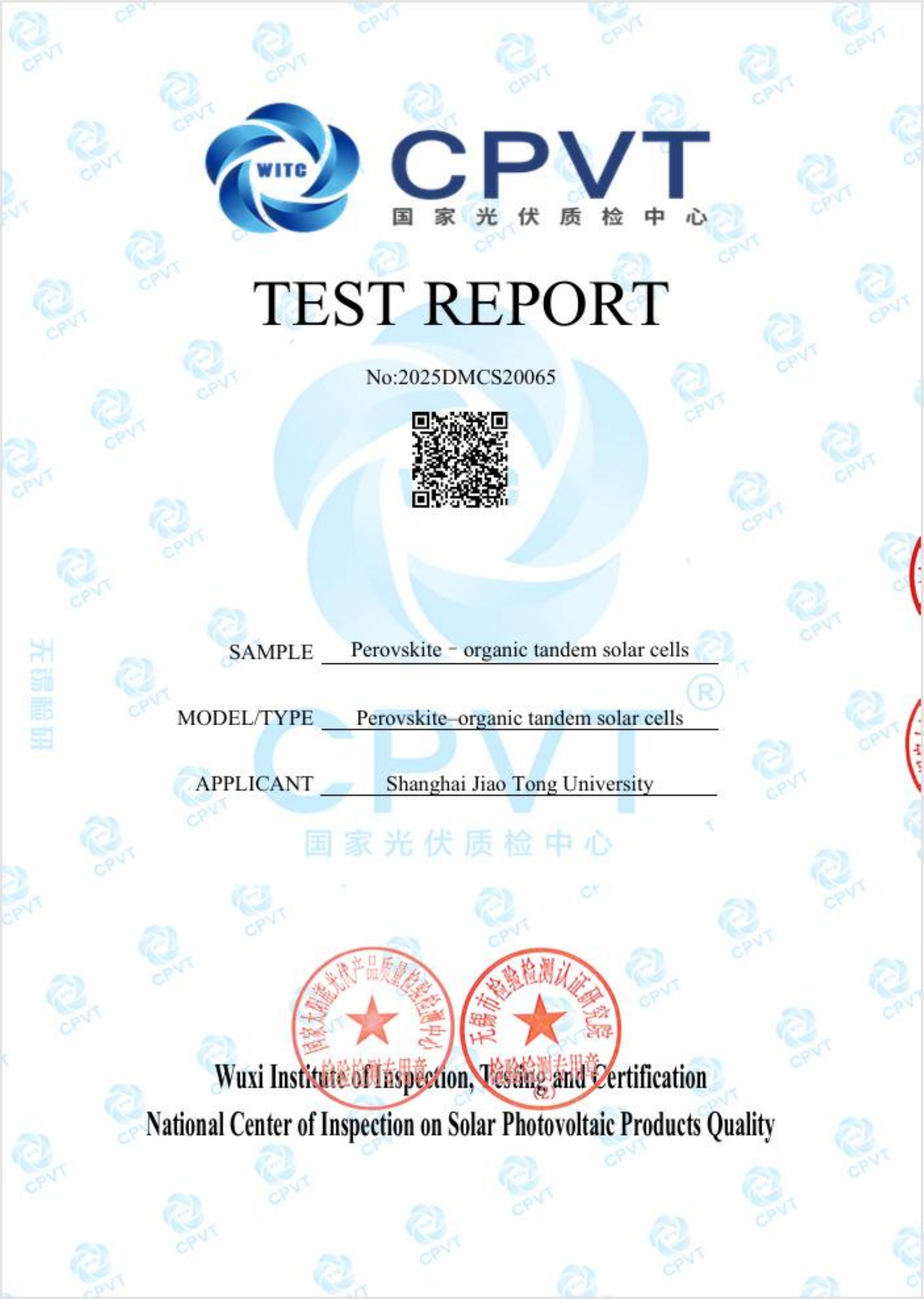
CPVT
国家光伏质检中心

TEST REPORT

No:2025DMCS20065

SAMPLE Perovskite－organic tandem solar cells

MODEL/TYPE Perovskite–organic tandem solar cells

APPLICANT Shanghai Jiao Tong University

国家太阳能光伏产品质量检验检测中心 检验检测专用章

无锡市检验检测认证研究院 检验检测专用章

Wuxi Institute of Inspection, Testing and Certification

National Center of Inspection on Solar Photovoltaic Products Quality

Supplementary Figure 27. Certification report of the perovskite-organic TSC by National Center of Inspection on Solar Photovoltaic Products Quality (Wuxi Institute of Inspection, Testing and Certification).

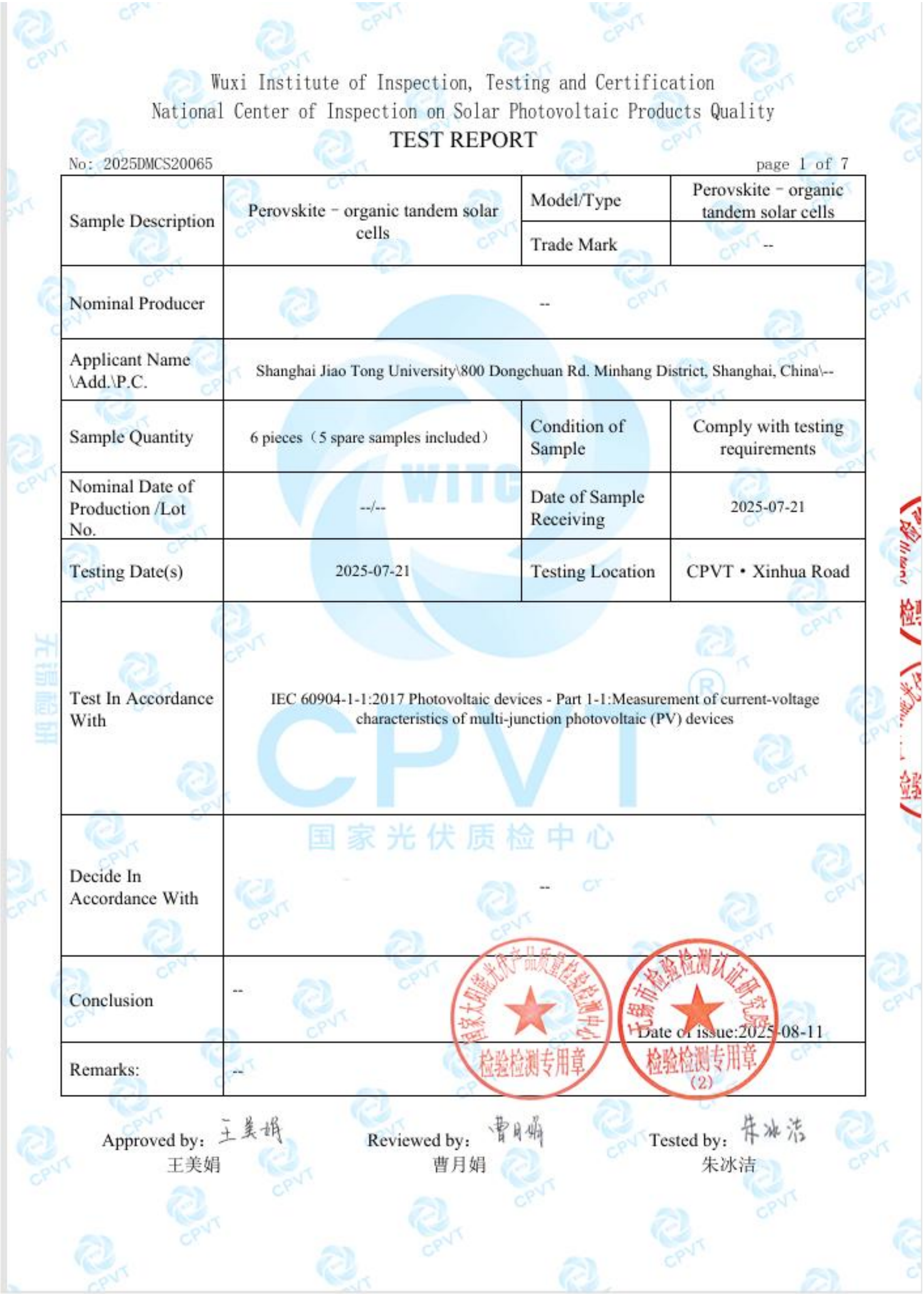

Wuxi Institute of Inspection, Testing and Certification
National Center of Inspection on Solar Photovoltaic Products Quality
TEST REPORT

No: 2025DMCS20065 page 1 of 7

Sample Description	Perovskite - organic tandem solar cells	Model/Type	Perovskite - organic tandem solar cells
		Trade Mark	--
Nominal Producer	--		
Applicant Name \Add.\P.C.	Shanghai Jiao Tong University\800 Dongchuan Rd. Minhang District, Shanghai, China\--		
Sample Quantity	6 pieces（5 spare samples included）	Condition of Sample	Comply with testing requirements
Nominal Date of Production /Lot No.	--/--	Date of Sample Receiving	2025-07-21
Testing Date(s)	2025-07-21	Testing Location	CPVT • Xinhua Road
Test In Accordance With	IEC 60904-1-1:2017 Photovoltaic devices - Part 1-1:Measurement of current-voltage characteristics of multi-junction photovoltaic (PV) devices		
Decide In Accordance With	--		
Conclusion	-- Date of issue:2025-08-11		
Remarks:	--		

Approved by: 王美娟 Reviewed by: 曹月娟 Tested by: 朱冰洁

Supplementary Figure 27. Certification report of the perovskite-organic TSC by National Center of Inspection on Solar Photovoltaic Products Quality (Wuxi Institute of Inspection, Testing and Certification).

No:2025DMCS20065

Test Results

Clause	Test item(s)	Unit	Technical requirements	Results	Verdict Pass/Fail
1	Current-voltage characteristic measurement (Forward scan)	—	At irradiance 1000W/m² (standard solar spectral irradiance distribution corresponds to IEC60904-3), measure the current-voltage characteristics of the sample with the variation of load.	—	—
1.1	Open-circuit voltage, Voc	V	—	2.199	—
1.2	Short-circuit current, Isc	mA	—	0.9009	—
1.3	Maximum-power, Pmax	mW	—	1.559	—
1.4	Maximum-power voltage, Vmp	V	—	1.900	—
1.5	Maximum-power current, Imp	mA	—	0.8205	—
1.6	Fill factor FF, %	—	—	78.69	—
1.7	Efficiency η, %	—	$\eta = \frac{P_{max}}{1000W/m^2 \times S} \times 100\%$ S denotes the area of the aperture mask.	25.14	—

Remark: Forward sweep direction: -0.1V~2.3V, step: 0.02V, delay time: 0s. $J_{sc} = \frac{I_{sc}}{S} = 14.53mA/cm^2$.
The area used to calculate J_{sc} and efficiency is determined by mask, S = 6.202 mm².

Supplementary Figure 27. Certification report of the perovskite-organic TSC by National Center of Inspection on Solar Photovoltaic Products Quality (Wuxi Institute of Inspection, Testing and Certification).

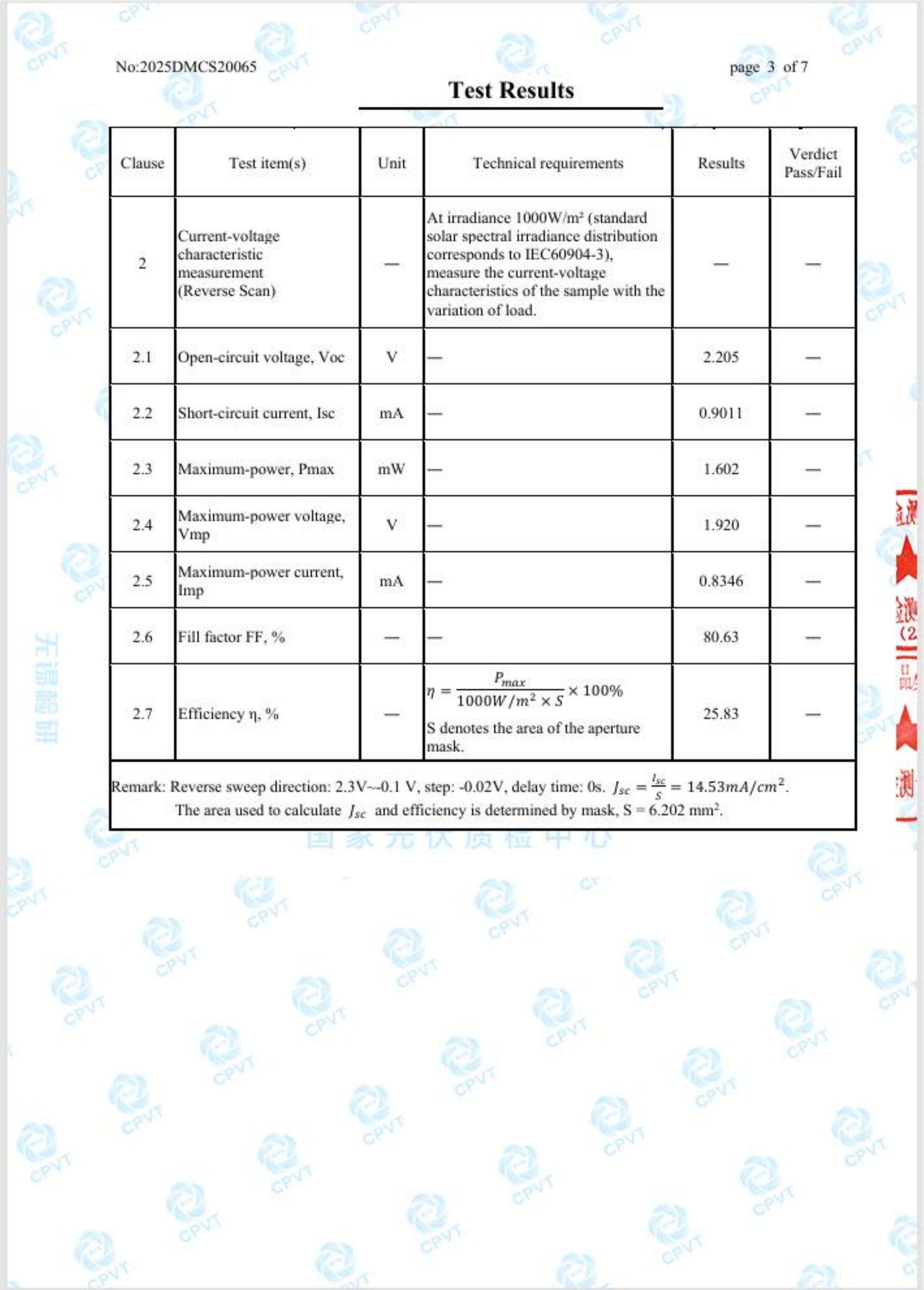

No:2025DMCS20065 page 3 of 7

Test Results

Clause	Test item(s)	Unit	Technical requirements	Results	Verdict Pass/Fail
2	Current-voltage characteristic measurement (Reverse Scan)	—	At irradiance 1000W/m² (standard solar spectral irradiance distribution corresponds to IEC60904-3), measure the current-voltage characteristics of the sample with the variation of load.	—	—
2.1	Open-circuit voltage, Voc	V	—	2.205	—
2.2	Short-circuit current, Isc	mA	—	0.9011	—
2.3	Maximum-power, Pmax	mW	—	1.602	—
2.4	Maximum-power voltage, Vmp	V	—	1.920	—
2.5	Maximum-power current, Imp	mA	—	0.8346	—
2.6	Fill factor FF, %	—	—	80.63	—
2.7	Efficiency η, %	—	$\eta = \frac{P_{max}}{1000W/m^2 \times S} \times 100\%$ S denotes the area of the aperture mask.	25.83	—

Remark: Reverse sweep direction: 2.3V~-0.1 V, step: -0.02V, delay time: 0s. $J_{sc} = \frac{I_{sc}}{S} = 14.53mA/cm^2$.
The area used to calculate J_{sc} and efficiency is determined by mask, S = 6.202 mm².

Supplementary Figure 27. Certification report of the perovskite-organic TSC by National Center of Inspection on Solar Photovoltaic Products Quality (Wuxi Institute of Inspection, Testing and Certification).

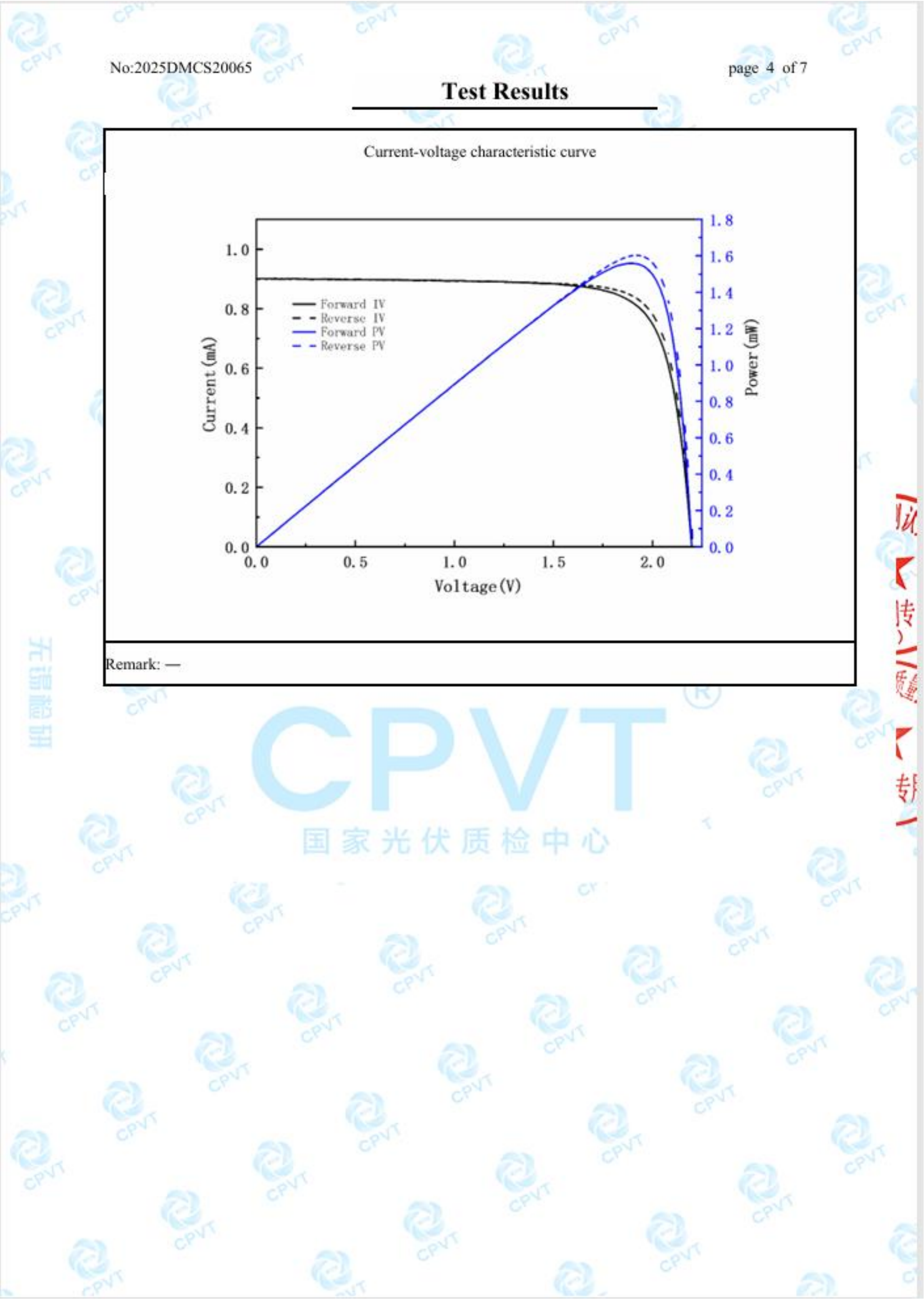

No:2025DMCS20065 page 4 of 7

Test Results

Current-voltage characteristic curve

Remark: —

CPVT
国家光伏质检中心

Supplementary Figure 27. Certification report of the perovskite-organic TSC by National Center of Inspection on Solar Photovoltaic Products Quality (Wuxi Institute of Inspection, Testing and Certification).

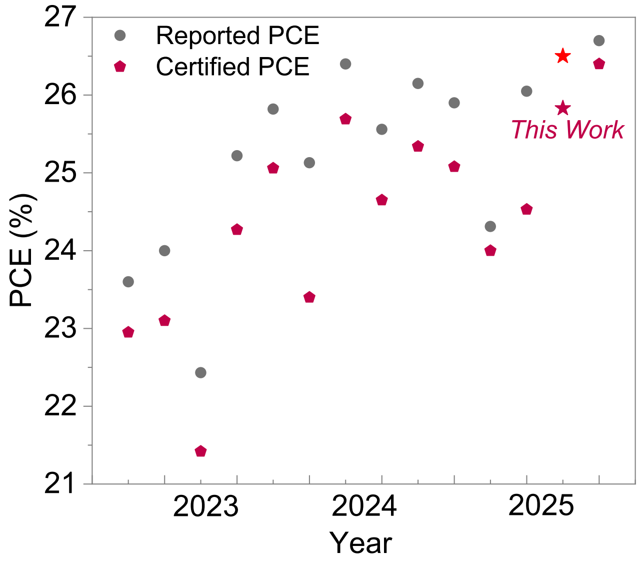

Supplementary Figure 28. PCE Summary of the reported perovskite-organic TSCs.

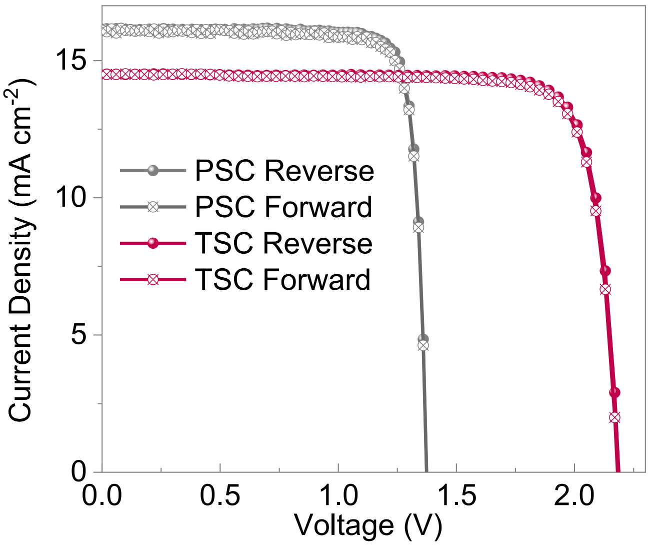

Supplementary Figure 29. *J-V* curves of PSC and TSC measured under AM1.5G illumination in reverse and forward scan.

Supplementary Note 4:

The resistance values were derived using the IVS-KA6000 software (Enlitech) based on the single-diode equivalent circuit model, which follows the equation:

$$I = I_L - I_0\left[\exp\left(\frac{q(V+IR_s)}{nkT}\right) - 1\right] - \frac{V+IR_s}{R_{sh}}$$

Where I, V, R_s, R_{sh}, I_0, n, q, **k** and T are the output current, output voltage, series resistance, shunt resistance, reverse saturation current of the diode, ideal factor,

elementary charge, Boltzmann constant and absolute temperature.

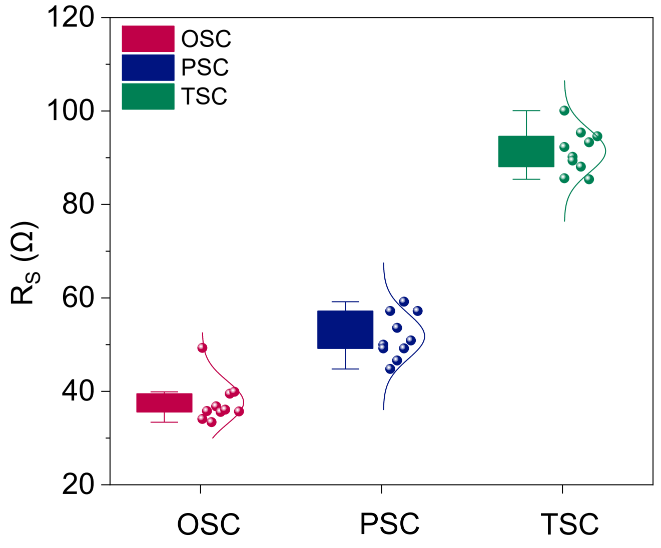

Supplementary Figure 30. The statistical series resistance values of OSCs, PSCs, and TSCs, error bars mean variance.

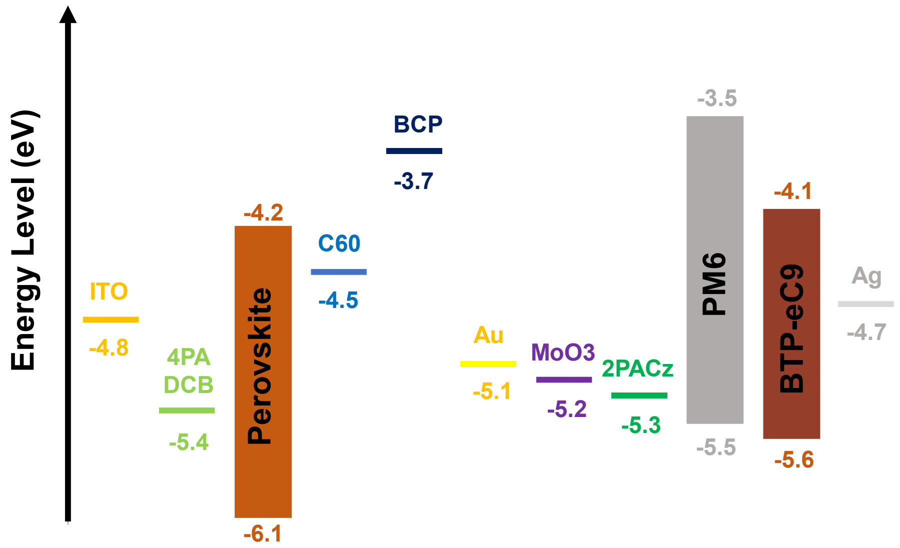

Supplementary Figure 31. The energy level diagram of the complete tandem device.

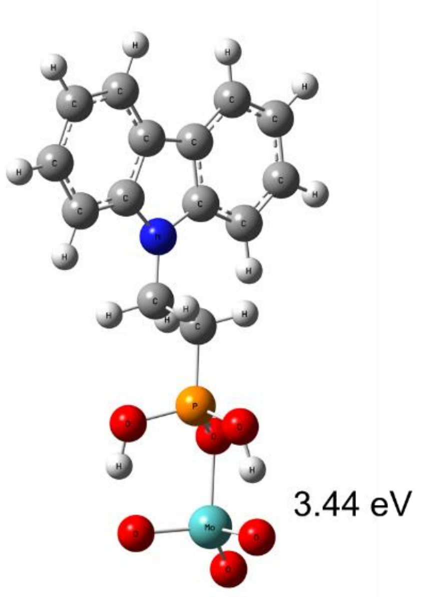

Supplementary Figure 32. The Mo–O–P bond connects 2PACz with MoO_3, as obtained through first-principles density functional theory.

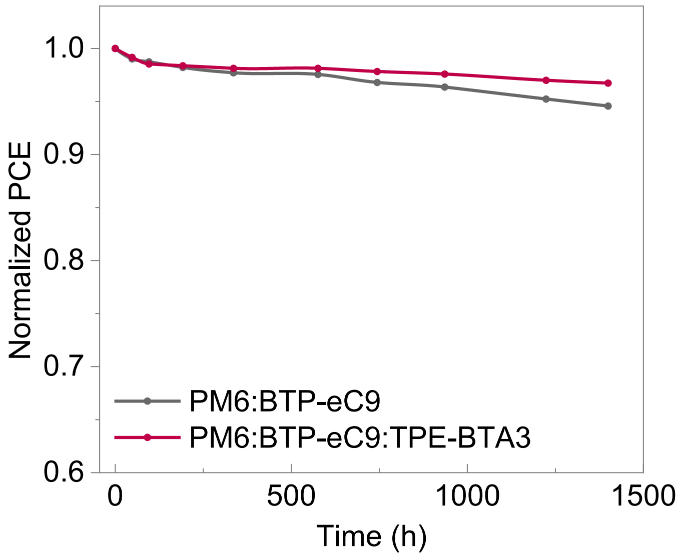

Supplementary Figure 33. Long-term storage stability of OSCs in nitrogen. The initial efficiency of OSCs with and without TPE-BTA3 are 19.4% and 18.0%, respectively.

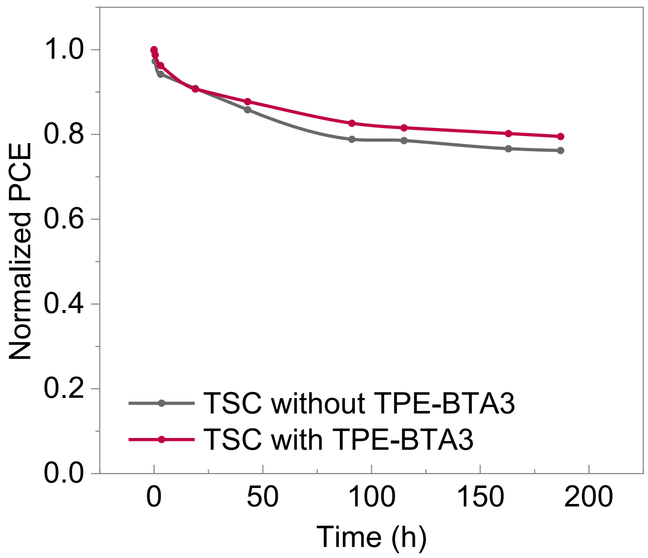

Supplementary Figure 34. Long-term photostability of the unencapsulated tandem devices at 85°C. The initial efficiency of TSCs with and without TPE-BTA3 are 26.3% and 25.6%, respectively.

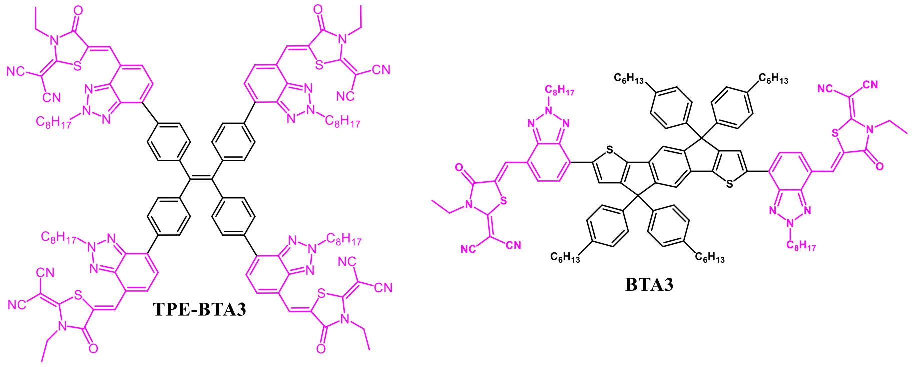

Supplementary Figure 35. The molecular structures of TPE-BTA3 and BTA3.

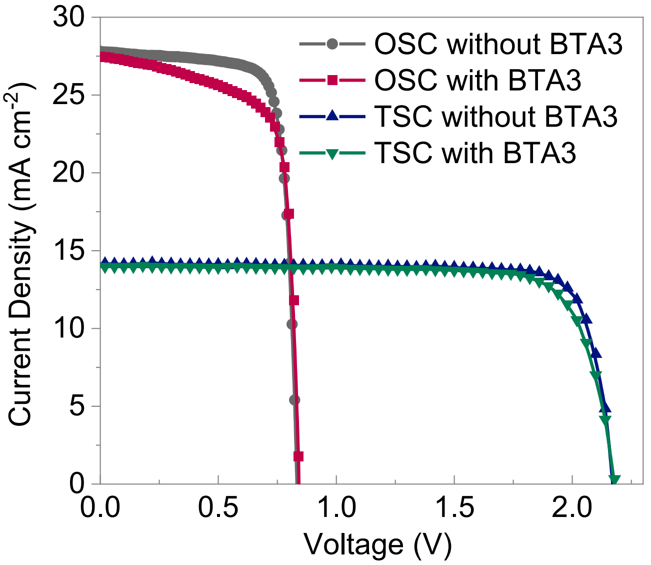

Supplementary Figure 36. *J-V* curves of PSC and TSC without/with BTA3.

Supplementary Table 1. Water and n-Hexadecane contact angle for PM6, BTP-eC9, and TPE-BTA3.

Surface	PM6	BTP-eC9	TPE-BTA3
θ_{Water} (°)	107.2	94.1	88.9
$\theta_{n\text{-}Hexadecane}$ (°)	9.2	3.4	4.5
γ (mN m^{-1})	20.7	23.6	25.5

Supplementary Table 2. Photovoltaic performance of PM6:BTP-eC9:TPE-BTA3 at different ratios.

Compound	V_{OC} (V)	J_{SC} (mA cm^{-2})	FF (%)	PCE (%)
PM6:BTP-eC9 1:1.2	0.834	27.8	78.7	18.3
PM6:BTP-eC9:TPE-BTA3 1:1.2:0.06	0.852	28.2	78.9	18.9
PM6:BTP-eC9:TPE-BTA3 1:1.2:0.12	0.860	28.9	79.4	19.7
PM6:BTP-eC9:TPE-BTA3 1:1.2:0.18	0.864	28.1	77.0	18.7
PM6:BTP-eC9:TPE-BTA3 1:0:1.2	1.223	6.3	33.2	2.6

Supplementary Table 3. The lifetime of TRPL that probe at 620 nm (TPE-BTA3), 670 nm (PM6), and 860 nm (BTP-eC9) in different films.

Probe at	Lifetime	Film	A1	t1	A2	t2
620 nm	2805 ps	Pure TPE-BTA3	40.7%	1354 ps	59.3%	3802 ps
620 nm	305 ps	PM6:TPE-BTA3	6.3%	3636 ps	93.7%	80.6 ps
620 nm	150 ps	BTP-eC9:TPE-BTA3	96.6%	47.3 ps	3.4%	3076 ps
670 nm	275 ps	Pure PM6	98.1%	280 ps	1.9%	3 ps
670 nm	550 ps	PM6:TPE-BTA3	91.4%	196 ps	8.6%	4301 ps
860 nm	1099 ps	Pure-BTP-eC9	99.2%	1051 ps	0.8%	5838 ps
860 nm	1173 ps	BTP-eC9:TPE-BTA3	100%	1173 ps	/	/

Supplementary Table 4. Detailed energy loss of PM6:BTP-eC9 and PM6:BTP-eC9:TPE-BTA3.

Active layer	E_{gap} (eV)	V_{OC} (V)	E_{loss} (eV)	V_{OC}^{SQ} (V)	ΔE_1 (eV)	$V_{\mathrm{OC}}^{\mathrm{rad}}$ (V)	ΔE_2 (eV)	ΔE_3 (eV)
PM6:BTP-eC9	1.382	0.836	0.546	1.122	0.26	1.065	0.057	0.229
PM6:BTP-eC9:TPE-BTA3	1.382	0.853	0.529	1.122	0.26	1.071	0.052	0.217

Supplementary Table 5. Statistical energy loss of PM6:BTP-eC9 and PM6:BTP-eC9:TPE-BTA3.

Compound	ΔE_3 (eV)	ΔE_2 (eV)
PM6:BTP-eC9	0.229±0.001	0.057±0.001
PM6:BTP-eC9: TPE-BTA3	0.218±0.001	0.052±0.001

Supplementary Table 6. Detailed energy loss of PM6:TPE-BTA3.

Active layer	E_{gap} (eV)	V_{OC} (V)	E_{loss} (eV)	V_{OC}^{SQ} (V)	ΔE_1 (eV)	$V_{\mathrm{OC}}^{\mathrm{rad}}$ (V)	ΔE_2 (eV)	ΔE_3 (eV)
PM6:TPE-BTA3	1.886	1.222	0.664	1.594	0.292	1.468	0.126	0.245

Supplementary Table 7. Detailed GIWAXS data of Pure-BTP-eC9, BTP-eC9:TPE-BTA3, PM6:BTP-eC9:TPE-BTA3, and PM6:BTP-eC9:TPE-BTA3.

Film	OOP				IP			
	(010)				(100)			
	q (Å^{-1})	d-spacing (Å)	FWHM (Å^{-1})	CCL (Å)	q (Å^{-1})	d-spacing (Å)	FWHM (Å^{-1})	CCL (Å)
BTP-eC9	1.709	3.677	0.287	14.70	0.390	16.13	0.089	47.15
BTP-eC9:TPE-BTA3	1.708	3.680	0.294	14.32	0.389	16.15	0.095	44.28
PM6:BTP-eC9	1.706	3.683	0.269	15.66	0.306	20.51	0.081	52.04
PM6:BTP-eC9:TPE-BTA3	1.703	3.690	0.271	15.58	0.305	20.58	0.082	51.34

Supplementary Table 8. Charge mobility of PM6:BTP-eC9 and PM6:BTP-eC9:TPE-BTA3 calculated by SCLC (the average data obtained from 5 independent devices).

Films	Hole mobility μ_h (10^{-4} cm^2 V^{-1} s^{-1})	Electron mobility μ_e (10^{-4} cm^2 V^{-1} s^{-1})	μ_h/μ_e
PM6:BTP-eC9	5.9 ± 0.8	4.1 ± 0.9	1.44
PM6:BTP-eC9:TPE-BTA3	8.1 ± 0.7	6.3 ± 1.0	1.29

Supplementary Table 9. Summary of photovoltaic performance of the reported perovskite-organic TSCs from literatures.

Number	Reported PCE (%)	Certified PCE (%)	Reference
1	23.6	22.95	*Nat. Energy 7, 229-237 (2022)*
2	24.0	23.1	*Nature 604, 280-286 (2022)*
3	22.43	21.42	*Adv. Funct. Mater. 33, 2212599 (2023)*
4	25.22	24.27	*Nat Energy 9, 411-421 (2024).*
5	25.82	25.06	*Nat Energy 9, 592-601 (2024)*
6	25.13	23.40	*Joule 8, 2554-2569, (2024)*
7	26.4	25.69	*Nature **635**, 860–866 (2024)*
8	25.56	24.65	*Adv. Mater. 2024, 36, 2410692*
9	26.15	25.34	*Angew. Chem. Int. Ed. 2025, e202502391*
10	25.90	25.08	*Nat Energy **10**, 513–525 (2025)*
11	24.31	24.00	*Adv. Mater. 2025, 2500190*
12	26.05	24.53	*Nat Commun **16**, 2759 (2025)*
13	27.5	26.4	*Nature 643, 104–110 (2025)*
14	**26.5**	**25.83**	***This Work***

Supplementary Table 10. Photovoltaic parameters of OSCs and TSCs that without/with BTA3.

Compound	V_{OC} (V)	J_{SC} (mA cm^{-2})	FF (%)	PCE (%)
PM6:BTP-eC9 1:1.2	0.834	27.8	78.7	18.3
PM6:BTP-eC9: BTA3 1:1.2:0.12	0.842	27.5	73.4	17.0
TSC Without BTA3	2.177	14.2	82.4	25.4
TSC With BTA3	2.182	14.0	79.4	24.3

Supplementary References

1 Chen, W. *et al.* Monolithic perovskite/organic tandem solar cells with 23.6% efficiency enabled by reduced voltage losses and optimized interconnecting layer. *Nat. Energy* **7**, 229-237 (2022). https://doi.org:10.1038/s41560-021-00966-8

2 Brinkmann, K. O. *et al.* Perovskite–organic tandem solar cells with indium oxide interconnect. *Nature* **604**, 280-286 (2022). https://doi.org:10.1038/s41586-022-04455-0

3 Yao, Q. *et al.* Dual Sub-Cells Modification Enables High-Efficiency n–i–p Type Monolithic Perovskite/Organic Tandem Solar Cells. *Adv. Funct. Mater.* **33**, 2212599 (2023). https://doi.org:https://doi.org/10.1002/adfm.202212599

4 Wu, S. *et al.* Redox mediator-stabilized wide-bandgap perovskites for monolithic perovskite-organic tandem solar cells. *Nat. Energy* **9**, 411-421 (2024). https://doi.org:10.1038/s41560-024-01451-8

5 Zhang, Z. *et al.* Suppression of phase segregation in wide-bandgap perovskites with thiocyanate ions for perovskite/organic tandems with 25.06% efficiency. *Nat. Energy* **9**, 592-601 (2024). https://doi.org:10.1038/s41560-024-01491-0

6 Guo, X. *et al.* Stabilizing efficient wide-bandgap perovskite in perovskite-organic tandem solar cells. *Joule.* **8**, 2554-2569 (2024). https://doi.org:https://doi.org/10.1016/j.joule.2024.06.009

7 Jiang, X. *et al.* Isomeric diammonium passivation for perovskite–organic tandem solar cells. *Nature* **635**, 860-866 (2024). https://doi.org:10.1038/s41586-024-08160-y

8 Wu, X. *et al.* Optimization of Charge Extraction and Interconnecting Layers for Highly Efficient Perovskite/Organic Tandem Solar Cells with High Fill Factor. *Adv. Mater.* **36**, 2410692 (2024). https://doi.org:https://doi.org/10.1002/adma.202410692

9 Dong, P. *et al.* Retarding Phase Segregation via Lattice Reinforcement for Efficient and Stable Perovskite/Organic Tandems. *Angew. Chem. Int. Ed.*, e202502391 (2025). https://doi.org:https://doi.org/10.1002/anie.202502391

10 Han, Y. *et al.* Inorganic perovskite/organic tandem solar cells with 25.1% certified efficiency via bottom contact modulation. *Nat. Energy* (2025). https://doi.org:10.1038/s41560-025-01742-8

11 Chen, X. *et al.* Efficient Perovskite/Organic Tandem Photovoltaic Devices and Large-area Modules Featuring Thick-Film Organic Solar Cells. *Adv. Mater.*, 2500190 (2025). https://doi.org:https://doi.org/10.1002/adma.202500190

12 An, Y. *et al.* Balancing carrier transport in interconnection layer for efficient perovskite/organic tandem solar cells. *Nat. Commun.* **16**, 2759 (2025). https://doi.org:10.1038/s41467-025-58047-3

13 Jia, Z. *et al.* Efficient near-infrared harvesting in perovskite–organic tandem solar cells. *Nature* **643**, 104-110 (2025). https://doi.org:10.1038/s41586-025-09181-x
