# Supplementary material for: Aggregation-induced emission luminogen in ternary organic bulk-heterojunction for efficient perovskite-organic tandem solar cells
Source: Nat Commun. 2026 May 30;17:7019. doi: 10.1038/s41467-026-73743-4 (PMC13392458; doi:10.1038/s41467-026-73743-4)
Supplement: Supplementary file 2 — Reporting Summary [file 41467_2026_73743_MOESM2_ESM.pdf]

Corresponding author(s): Zhibin Yang, Erjun Zhou

Solar Cells Reporting Summary

Nature Research wishes to improve the reproducibility of the work that we publish. This form is intended for publication with all accepted papers reporting the characterization of photovoltaic devices and provides structure for consistency and transparency in reporting. Some list items might not apply to an individual manuscript, but all fields must be completed for clarity.

For further information on Nature Research policies, including our data availability policy, see Authors & Referees.

Experimental design

Please check: are the following details reported in the manuscript?

1. Dimensions

Item	Yes/No	Details
Area of the tested solar cells	☒ Yes ☐ No	The active area was 0.08 cm2.
Method used to determine the device area	☒ Yes ☐ No	The device area is defined by a metal mask corrected using a microscope.

2. Current-voltage characterization

Item	Yes/No	Details
Current density-voltage (J-V) plots in both forward and backward direction	☒ Yes ☐ No	Supplementary Figures S28.
Voltage scan conditions<br>*For instance: scan direction, speed, dwell times*	☒ Yes ☐ No	Reverse /forward scan, 0.04 V/s, 10 ms.
Test environment<br>*For instance: characterization temperature, in air or in glove box*	☒ Yes ☐ No	J-V curves were measured in a glove box at room temperature. The photostability of the encapsulated devices were measured in air at room temperature.
Protocol for preconditioning of the device before its characterization	☐ Yes ☒ No	No preconditioning is operated before characterization.
Stability of the J-V characteristic<br>*Verified with time evolution of the maximum power point or with the photocurrent at maximum power point; see ref. 7 for details.*	☒ Yes ☐ No	For long-term photostability, we don't have the equipment to track MPP, but we tracked the PCE that changes over time (Figure 4e).

3. Hysteresis or any other unusual behaviour

Item	Yes/No	Details
Description of the unusual behaviour observed during the characterization	☒ Yes ☐ No	WBG PSCs and TSCs exhibit a little hysteresis within 5%.
Related experimental data	☒ Yes ☐ No	Supplementary Figures S28.

4. Efficiency

Item	Yes/No	Details
External quantum efficiency (EQE) or incident photons to current efficiency (IPCE)	☒ Yes ☐ No	Figures 2b and 4c.
A comparison between the integrated response under the standard reference spectrum and the response measure under the simulator	☒ Yes ☐ No	The comparison can be seen in Figure 4b and 4c.
For tandem solar cells, the bias illumination and bias voltage used for each subcell	☒ Yes ☐ No	The EQE of wide and narrow bandgap sub-cells in tandem cells were measured by respectively exposing the tandem cell under a 550 nm and 780 nm LED lamp for saturating the other junction during measurement without any bias voltage.

5. Calibration

Item	Yes/No	Details
Light source and reference cell or sensor used for the characterization	☒ Yes ☐ No	The J–V curves of all devices were tested under the illumination from a 3A solar simulator (CME-Sol 8040-3A, Microenerg Beijing Technology Co., Ltd). The light intensity of solar simulator was calibrated with a 2×2 cm2 reference mono silicon cell (Oriel PN 91150V, Newport, USA., calibrated by the National Renewable Energy Laboratory).

Confirmation that the reference cell was calibrated and certified	☒ Yes ☐ No	The light intensity of solar simulator was calibrated with a 2×2 cm2 reference mono silicon cell (Oriel PN 91150V, Newport, USA., calibrated by the National Renewable Energy Laboratory).
Calculation of spectral mismatch between the reference cell and the devices under test	☐ Yes ☒ No	A best AAA Solar Simulator was used for J-V measurement and the short-circuit current density from J-V curves and EQEs were compared.

6. Mask/aperture

Size of the mask/aperture used during testing	☒ Yes ☐ No	6.11 mm2
Variation of the measured short-circuit current density with the mask/aperture area	☐ Yes ☒ No	No obvious variation.

7. Performance certification

Identity of the independent certification laboratory that confirmed the photovoltaic performance	☒ Yes ☐ No	The tandem device were certified by National Center of Inspection on Solar Photovoltaic Products Quality (Wuxi Institute of Inspection, Testing and Certification).
A copy of any certificate(s)<br>*Provide in Supplementary Information*	☒ Yes ☐ No	Supplementary Figure S26.

8. Statistics

Number of solar cells tested	☒ Yes ☐ No	30 tandem cells were fabricated and measured to know the efficiency distribution.
Statistical analysis of the device performance	☒ Yes ☐ No	Figure 4d.

9. Long-term stability analysis

Type of analysis, bias conditions and environmental conditions<br>*For instance: illumination type, temperature, atmosphere humidity, encapsulation method, preconditioning temperature*	☒ Yes ☐ No	As shown in Figure 4e, the long-term photostability of the devices were measured under a full spectrum LED illumination with an intensity equivalent to 1 sun in ambient conditions with humidity of 30-50%. The devices were encapsulated with UV-curable adhesive and slide glass before measurement.
