# Supplementary material for: Aggregation-induced emission luminogen in ternary organic bulk-heterojunction for efficient perovskite-organic tandem solar cells
Source: Nat Commun. 2026 May 30;17:7019. doi: 10.1038/s41467-026-73743-4 (PMC13392458; doi:10.1038/s41467-026-73743-4)
Supplement: Supplementary file 3 — Transparent Peer Review file [file 41467_2026_73743_MOESM3_ESM.pdf]

nature portfolio

Peer Review File

Aggregation-Induced Emission Luminogen in Ternary Organic Bulk-heterojunction for Efficiency Perovskite-Organic Tandem Solar Cells

Corresponding Author: Professor Zhibin Yang

This manuscript has been previously reviewed at another journal. This document only contains information relating to versions considered at Nature Communications.

This file contains all reviewer reports in order by version, followed by all author rebuttals in order by version.

Version 0:

Reviewer comments:

Reviewer #3

(Remarks to the Author)
The authors introduce an AIE-active acceptor, TPE-BTA3, as a third component in the PM6:BTP-eC9 system, achieving an improvement in single-junction efficiency from 18.3% to 19.7% (with 10% TPE-BTA3 as the optimum). By integrating this optimized organic sub-cell with a 1.85 eV wide-bandgap perovskite, they further demonstrate a monolithic perovskite–organic tandem device with a PCE of 26.5% (certified 25.83%). The manuscript also provides relatively systematic analyses of energy loss, GIWAXS, charge transport, and device stability. While the work is interesting and potentially impactful, several key issues have to be addressed before publication.

1. Evidence for AIE behavior
The authors regulate aggregation using chloroform/n-hexane mixed solvents and report enhanced and blue-shifted PL intensity with increasing f_n, together with increased particle size and PLQY, to support the AIE effect. However, the solution concentration is not specified, and the dependence of PLQY on excitation intensity is not discussed. Without these controls, the conclusion that the PLQY enhancement is purely aggregation-induced remains incomplete. Quantitative normalization of concentration and excitation conditions is required.

2. Identification of alloy-like acceptors
The authors infer the formation of alloy-like acceptors based on energy-level “centering” after mixing and on surface tension / Flory–Huggins χ parameters (with a smaller χ between TPE-BTA3 and BTP-eC9). While suggestive, these arguments do not conclusively demonstrate alloy formation. The authors are encouraged to include differential scanning calorimetry (DSC) and more detailed GIWAXS analysis (e.g., peak evolution, coherence length changes) to strengthen this claim.

3. Origin of transport degradation in neat TPE-BTA3 films
When TPE-BTA3 is used as the sole acceptor, the authors observe abnormally high ΔE_2 and ΔE_3 values and attribute this to disordered packing and discontinuous charge transport. However, from a chemical design perspective, it remains unclear which structural motifs are responsible for strong AIE and high PLQY, and which structural features are detrimental to charge transport. This distinction is critical for establishing generalizable AIEgen design rules. The authors should therefore clearly articulate:
o why TPE-BTA3 was selected as an AIE acceptor,
o the molecular design principles enabling strong AIE behavior,
o and the selection criteria for AIE molecules in the context of organic solar cells.

4. Energy-transfer mechanism (Förster vs Dexter)
Based on PL spectral overlap and TRPL measurements, the authors report a drastic reduction in the TPE-BTA3 lifetime (from 2760 ps to 305/150 ps) and a prolonged lifetime in the PM6/BTP-eC9 host, and attribute this to Förster resonance energy transfer (FRET). However, the manuscript lacks direct evidence excluding Dexter energy transfer. The authors should provide justification (e.g., donor–acceptor separation, spectral overlap integral, spin considerations) and

quantitatively estimate the energy-transfer efficiency.

5. Statistical significance of energy-loss reduction
The reduction in non-radiative energy loss ΔE_3 (0.229 → 0.217 eV) and the small change in ΔE_2 (0.057 → 0.052 eV) are central to the manuscript's conclusions. However, error bars and statistical significance are not provided. The authors should report device-to-device statistics, including mean values and standard deviations, and clearly describe measurement calibration procedures, such as EQE spectral integration, light-source calibration, and injected current ranges.

6. Urbach energy analysis
The reported decrease in Urbach energy (0.024 → 0.022 eV) is rather small. Additional experimental or statistical evidence is needed to convincingly demonstrate a meaningful reduction in energetic disorder.

7. Electrical characterization of tandem devices
The tandem architecture (ITO/4PADCB/perovskite/C60/BCP/Au/MoO_3/2-PACz/organic/PNDIT-F3N/Ag) is supported mainly by cross-sectional SEM (Fig. S24). However, electrical validation of the tandem device is insufficient. The authors are encouraged to provide measurements of contact resistance, series resistance, or impedance spectroscopy, as well as a discussion of the energy-level alignment and interfacial chemical stability of the Au/MoO_3/2-PACz recombination layer.

8. Stability assessment and broader tandem perspective
The reported stability tests are relatively short. Longer-term stability measurements are recommended, with clear specification of temperature control, spectral mismatch correction, and active vs aperture area. In addition, the authors should discuss the advantages and limitations of:
o perovskite–organic tandems,
o perovskite–perovskite tandems,
o perovskite–silicon tandems,
as well as the corresponding performance-improvement strategies. The rationale for considering perovskite–organic tandem solar cells as a promising route should be clearly articulated.

9. Control experiments to isolate the AIE effect
Since the authors attribute the performance enhancement primarily to the AIE effect of TPE-BTA3, it is essential to rule out a generic additive effect. The authors should therefore fabricate single-junction and tandem devices using structurally similar third components that do not exhibit AIE behavior, and compare their performance accordingly.

Version 1:

Reviewer comments:

Reviewer #3

(Remarks to the Author)
The authors have revised the manuscript carefully according to all of the comments. Therefore, I would like to recommend the manuscript for publication without any further revision.

Open Access This Peer Review File is licensed under a Creative Commons Attribution 4.0 International License, which permits use, sharing, adaptation, distribution and reproduction in any medium or format, as long as you give appropriate credit to the original author(s) and the source, provide a link to the Creative Commons license, and indicate if changes were made.
In cases where reviewers are anonymous, credit should be given to 'Anonymous Referee' and the source.
The images or other third party material in this Peer Review File are included in the article's Creative Commons license, unless indicated otherwise in a credit line to the material. If material is not included in the article's Creative Commons license and your intended use is not permitted by statutory regulation or exceeds the permitted use, you will need to obtain permission directly from the copyright holder.
To view a copy of this license, visit https://creativecommons.org/licenses/by/4.0/

Response Letter

Reviewer #3 (Remarks to the Author):

The authors introduce an AIE-active acceptor, TPE-BTA3, as a third component in the PM6:BTP-eC9 system, achieving an improvement in single-junction efficiency from 18.3% to 19.7% (with 10% TPE-BTA3 as the optimum). By integrating this optimized organic sub-cell with a 1.85 eV wide-bandgap perovskite, they further demonstrate a monolithic perovskite–organic tandem device with a PCE of 26.5% (certified 25.83%). The manuscript also provides relatively systematic analyses of energy loss, GIWAXS, charge transport, and device stability. While the work is interesting and potentially impactful, several key issues have to be addressed before publication.

Response: We appreciate your positive evaluation of our work. The manuscript has been carefully revised, and all your concerns have been addressed accordingly. We hope these efforts further enhance the quality of this work and offer new insights into the application of aggregation-induced emission molecules in organic solar cells. For your convenience, the main revisions are highlighted in blue in the revised manuscript and supplementary information.

1. Evidence for AIE behavior

The authors regulate aggregation using chloroform/n-hexane mixed solvents and report enhanced and blue-shifted PL intensity with increasing f_n, together with increased particle size and PLQY, to support the AIE effect. However, the solution concentration is not specified, and the dependence of PLQY on excitation intensity is not discussed. Without these controls, the conclusion that the PLQY enhancement is purely aggregation-induced remains incomplete. Quantitative normalization of concentration and excitation conditions is required.

Response: We appreciate your insightful question. For the AIE property measurements of TPE-BTA3, the solution concentration was fixed at 0.001 mol/L. The degree of molecular aggregation was modulated by varying the volume ratio of good solvent to poor solvent. We have added this information on page 5 of the revised manuscript.

To further verify the AIE effect as suggested, we performed additional photoluminescence measurements under varying excitation light intensities. The PL spectra obtained at different solvent volume fractions in **Fig. 1b** were recorded using an OD 2.0 filter with a transmittance of 0.1%. As shown in **Fig. S5**, when the excitation light intensity was increased tenfold with the OD 1.0 filter, the fluorescence emission intensity of TPE-BTA3 still exhibited a clear enhancement upon increased aggregation. The PLQY value of TPE-BTA3 increased from 35.7% to 54.5% as the water fraction f_n rose from 0% to 70%. It should be noted that under high-intensity excitation, AIE-active molecules may undergo excited-state absorption, which can introduce extra non-radiative decay channels and thus negatively affect the PLQY. Despite this effect, the above results clearly confirm that TPE-BTA3 exhibits distinct AIE behavior, which is not significantly dependent on the excitation intensity.

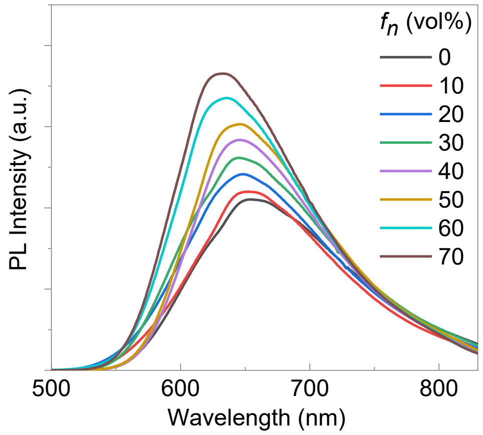

Fig. S5. PL spectra at varying solvent volume fractions measured with an OD 1.0 filter.

We have added the relevant discussion on page 6 of the revised manuscript. "To further verify the AIE effect, we performed additional photoluminescence measurements under enhanced excitation light intensity. As illustrated in **Fig. S5**, when the excitation light intensity was increased tenfold with the OD 1.0 filter, the fluorescence emission intensity of TPE-BTA3 still exhibited a clear enhancement upon increased aggregation. The PLQY value of TPE-BTA3 increased from 35.7% to 54.5% as the water fraction f_n rose from 0% to 70%. It should be noted that under high-intensity excitation, AIE-active molecules may undergo excited-state absorption, which can introduce extra non-radiative decay channels and thus negatively affect the PLQY. Despite this effect, the above results clearly confirm that TPE-BTA3 exhibits distinct AIE behavior, which is not dependent on the excitation intensity."

2. Identification of alloy-like acceptors

The authors infer the formation of alloy-like acceptors based on energy-level "centering" after mixing and on surface tension / Flory–Huggins χ parameters (with a smaller χ between TPE-BTA3 and BTP-eC9). While suggestive, these arguments do not conclusively demonstrate alloy formation. The authors are encouraged to include differential scanning calorimetry (DSC) and more detailed GIWAXS analysis (e.g., peak evolution, coherence length changes) to strengthen this claim.

Response: We appreciate your valuable suggestion. To further investigate the intermixing behavior of TPE-BTA3 and BTP-eC9, we first performed thermogravimetric analysis (TGA) on pure BTP-eC9, pure TPE-BTA3, and the BTP-eC9:TPE-BTA3 blend (10:1 weight ratio, consistent with the ternary device composition), as shown in **Fig. S8**. The thermal decomposition temperatures (T_d, corresponding to 5% weight loss) were measured to be 332 °C, 401 °C, and 337 °C for BTP-eC9, TPE-BTA3, and their blend, respectively. Notably, no additional decomposition peaks were observed in the thermogram of the blend, suggesting homogeneous molecular-scale mixing between the two components—an essential prerequisite for the formation of an alloy-like phase.

We subsequently attempted differential scanning calorimetry (DSC) measurements as recommended. However, no melting transition was observed for TPE-BTA3 even when heated to near its decomposition temperature. This phenomenon is

likely attributable to the extensive conjugated backbone and strong intermolecular π–π stacking interactions in TPE-BTA3, which are expected to significantly elevate its melting point. Given the limited thermal stability of the alkyl side chains, C–S bonds, and C=O bonds under ambient pressure, TPE-BTA3 decomposes prior to reaching its melting point upon heating. For safety considerations, the instrument administrator terminated the measurement before the decomposition temperature was reached to avoid potential hazards.

In addition, GIWAXS analysis (**Table S7**) reveals that both OOP and IP direction peaks of BTP-eC9:TPE-BTA3 films exhibited slight shifts toward lower $\boldsymbol{q}$ values compared to pure BTP-eC9 films, accompanied by a reduction in crystalline coherence lengths. The peak shape remained single-peaked throughout, further corroborating the formation of a homogeneous alloy phase rather than phase separation.

Collectively, the above experimental results and discussions provide more robust evidence to support the formation of alloy-like acceptors between TPE-BTA3 and BTP-eC9.

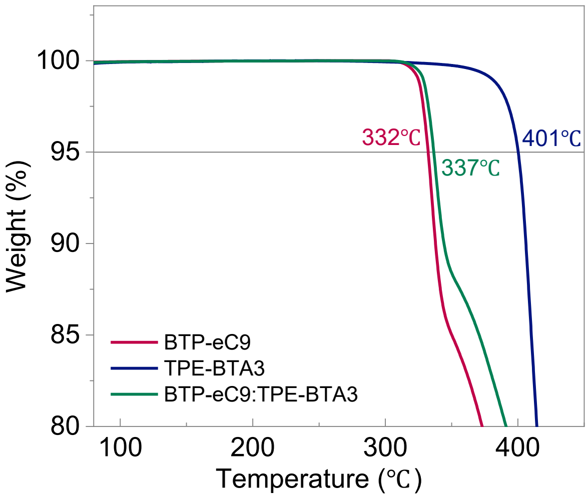

Fig. S8. Thermogravimetric analysis of pure BTP-eC9, pure TPE-BTA3, and BTP-eC9:TPE-BTA3 blend (10:1 weight ratio)

Table S7. Detailed GIWAXS data of Pure-BTP-eC9, BTP-eC9:TPE-BTA3, PM6:BTP-eC9:TPE-BTA3, and PM6:BTP-eC9:TPE-BTA3.

Film	OOP				IP			
	(010)				(100)			
	q (Å^{-1})	*d*-spacing (Å)	FWHM (Å^{-1})	CCL (Å)	*q* (Å^{-1})	*d*-spacing (Å)	FWHM (Å^{-1})	CCL (Å)
BTP-eC9	1.709	3.677	0.287	14.70	0.390	16.13	0.089	47.15
BTP-eC9:TPE-BTA3	1.708	3.680	0.294	14.32	0.389	16.15	0.095	44.28
PM6:BTP-eC9	1.706	3.683	0.269	15.66	0.306	20.51	0.081	52.04
PM6:BTP-eC9:TPE-BTA3	1.703	3.690	0.271	15.58	0.305	20.58	0.082	51.34

The relevant content has been added on pages 7 and 13 of the revised manuscript. "To investigate the intermixing behavior of TPE-BTA3 and BTP-eC9, we first performed thermogravimetric analysis (TGA) on pure BTP-eC9, pure TPE-BTA3, and the BTP-eC9:TPE-BTA3 blend (10:1 weight ratio, consistent with the ternary device composition). As shown in **Fig. S8**, the thermal decomposition temperatures (T_d, corresponding to 5% weight loss) were measured to be 332, 401, and 337 °C for BTP-eC9, TPE-BTA3, and their blend, respectively. Notably, no additional decomposition peaks were observed in the thermogram of the blend, suggesting homogeneous molecular-scale mixing between the two components, which is conducive to the formation of an alloy-acceptor." "Additionally, the BTP-eC9:TPE-BTA3 blend film maintained a single peak shape while exhibiting peak position shifts, further corroborating the formation of an alloy phase."

3. Origin of transport degradation in neat TPE-BTA3 films

When TPE-BTA3 is used as the sole acceptor, the authors observe abnormally high ΔE_2 and ΔE_3 values and attribute this to disordered packing and discontinuous charge transport. However, from a chemical design perspective, it remains unclear which structural motifs are responsible for strong AIE and high PLQY, and which structural features are detrimental to charge transport. This distinction is critical for establishing generalizable AIEgen design rules. The authors should therefore clearly articulate:

o why TPE-BTA3 was selected as an AIE acceptor,

o the molecular design principles enabling strong AIE behavior,

o and the selection criteria for AIE molecules in the context of organic solar cells.

Response: We appreciate your insightful suggestion. Our group (Erjun Zhou) has long been committed to developing non-fullerene small molecules based on benzotriazole (BTA) units. In our previous work, BTA3 has demonstrated a significant effect in reducing voltage loss (Adv. Mater. 2020, 32, 2002122; Adv. Mater. 2021, 33, 2101090). Building on this foundation, we designed TPE-BTA3 by integrating the classic BTA3 unit with tetraphenylethylene (TPE, a well-established AIE center core), in order to

achieve better photovoltaic performance.

According to the fundamental mechanism of AIE, the emergence of AIE activity typically relies on a special molecular structure, which features a central core that is connected with sterically hindered side chains via freely rotatable single bonds. This structure permits molecules to freely rotate in weakly aggregated states, but not in strongly aggregated states. The larger side chains and more twisted molecular conformations facilitate the attainment of stronger AIE effects.

However, our investigation reveals that the application of AIE molecules in OSCs is not straightforward and must be tailored to specific device configurations and functional requirements. When incorporating a small proportion of AIE molecules into ternary blend systems, the primary goal is to ensure strong luminescence efficiency, even if the molecule lacks ordered π-π stacking. In contrast, for binary systems where the AIE molecule serves as the only donor/acceptor, the structural constraints become significantly more stringent. In such cases, the AIE property cannot originate from the molecule's central core; instead, appropriate modifications to the side chains are necessary to preserve the intrinsic ordered π–π stacking of the parent molecule, thereby ensuring efficient charge transport (Advanced Materials (2026): e19588. https://doi.org/10.1002/adma.202519588).

We appreciate the opportunity to elaborate on this point and thank you for the valuable feedback. We have added a corresponding discussion on pages 4 and 13 of the manuscript.

"We have long been committed to developing non-fullerene small molecules based on benzotriazole (BTA) units. In our previous work, BTA3 has demonstrated a significant effect in reducing voltage loss. Building on this foundation, we designed TPE-BTA3 by integrating the classic BTA3 unit with TPE, in order to achieve better photovoltaic performance." "The application of AIE molecules in OSCs requires careful tailoring to device configurations and functional demands. In ternary blends with a small AIE component, the emphasis is on achieving high luminescence efficiency. However, in binary systems where the AIE molecule acts as the sole donor or acceptor, stricter structural control is essential, the AIE characteristic should not originate from the central core, the appropriate side-chain modifications are required to preserve the intrinsic ordered π–π stacking necessary for efficient charge transport."

4. Energy-transfer mechanism (Förster vs Dexter)

Based on PL spectral overlap and TRPL measurements, the authors report a drastic reduction in the TPE-BTA3 lifetime (from 2760 ps to 305/150 ps) and a prolonged lifetime in the PM6/BTP-eC9 host, and attribute this to Förster resonance energy transfer (FRET). However, the manuscript lacks direct evidence excluding Dexter energy transfer. The authors should provide justification (e.g., donor–acceptor separation, spectral overlap integral, spin considerations) and quantitatively estimate the energy-transfer efficiency.

Response: We thank the reviewer for this valuable suggestion. While our manuscript did not explicitly categorize energy transfer as Förster or Dexter type, we agree that a deeper investigation into the underlying mechanism is highly meaningful and have thus

conducted additional analyses as outlined below.

First, it is essential to clarify the fundamental characteristics and distinctions between the two energy transfer mechanisms. Förster resonance energy transfer is a non-contact, long-range process mediated by dipole–dipole interactions, typically effective over distances of 1–10 nm. Its efficiency is inversely proportional to the sixth power of the donor–acceptor distance ($\propto 1/r^6$) and requires substantial spectral overlap between the donor's emission and the acceptor's absorption, as well as a high PLQY of the donor. In contrast, Dexter energy transfer is a short-range, contact-dependent process involving electron exchange, with an effective distance of less than 1 nm and an efficiency that decays exponentially with distance ($\propto \exp(-\beta r)$). It necessitates direct orbital overlap between donor and acceptor and matching spin states.

In the PM6:BTP-eC9:TPE-BTA3 system studied here, TPE-BTA3 serves as the energy donor and exhibits a high PLQY. Its fluorescence emission spectrum shows significant overlap with the absorption spectra of both PM6 and BTP-eC9, as illustrated in **Fig. 2d**. Based on these characteristics, we propose that Förster resonance energy transfer should be the dominant mechanism in this system.

To experimentally validate this hypothesis, we designed experiments focusing on the spectral overlap integral. We constructed schematic diagrams (**Fig. R1**) showing the overlapping regions between the PL spectrum of TPE-BTA3 and the absorption spectra of PM6 and BTP-eC9 in both film and solution states. We then measured the energy transfer efficiency (reflected by the retained fluorescence intensity of TPE-BTA3), which was obtained after mixing TPE-BTA3 with PM6 or BTP-eC9 at a 1:1 ratio in both states. As shown in **Fig. R2**, when mixed with PM6, TPE-BTA3 retained 2.2% and 1.3% of its fluorescence intensity in film and solution, respectively (calculated at 600 nm to avoid interference from PM6's PL peak). When mixed with BTP-eC9, the retained intensities were 0.7% and 0.5%, respectively (calculated at 630 nm). Given that Dexter energy transfer depends solely on orbital overlap and is insensitive to changes in spectral overlap, the observed variations in transfer efficiency strongly support Förster resonance transfer as the primary pathway.

Furthermore, the explanation for the lower retained signal intensity observed in the solution state for both combinations is as follows: the spectral overlap integral is a wavelength-weighted integral, where shorter wavelengths (higher energy regions) carry greater weight. The spectra in the solution state exhibit a certain degree of blue shift, increasing the proportion of the spectral overlap region in the short-wavelength range. Consequently, the weighted spectral overlap increases significantly, leading to more efficient energy transfer in the solution state.

We also attempted to probe the distance dependence of energy transfer by inserting insulating layers (e.g., Al_2O_3 or LiF) of varying thicknesses (1–10 nm) between PM6 (or BTP-eC9) and TPE-BTA3 layers. However, this approach proved experimentally unfeasible due to solvent incompatibility; both the donor and acceptor layers were soluble in the same processing solvents, and the intermediate insulating layer could not adequately protect the underlying film during deposition. As a result, only bulk-heterojunction-like mixed films could be obtained, precluding a quantitative assessment of energy transfer efficiency as a function of distance.

Regarding the quantification of energy transfer efficiency, using the thin-film state shown in **Fig. R2** as the example, the fluorescence quenching efficiencies of TPE-BTA3 after blending with PM6 and BTP-eC9 are 97.8% and 99.3%, respectively. For BTP-eC9, as demonstrated in **Fig. S10**, no charge transfer occurs between BTP-eC9 and TPE-BTA3. Therefore, the 99.3% value can be attributed to the energy transfer efficiency from TPE-BTA3 to BTP-eC9. In contrast, the situation with PM6 is more complex. As shown in **Fig. S15a**, the PM6:TPE-BTA3 device achieves a short-circuit current density of approximately 5 mA/cm^2, indicating that a certain degree of charge transfer does occur between the two components. However, as illustrated in **Fig. R3**, the absorption and fluorescence spectra of PM6 exhibit substantial overlap with those of TPE-BTA3, making it difficult to spectroscopically deconvolute the contributions of energy transfer and charge transfer. Consequently, the actual energy transfer efficiency (TPE-BTA3 to BTP-eC9) is expected to be lower than 97.8%.

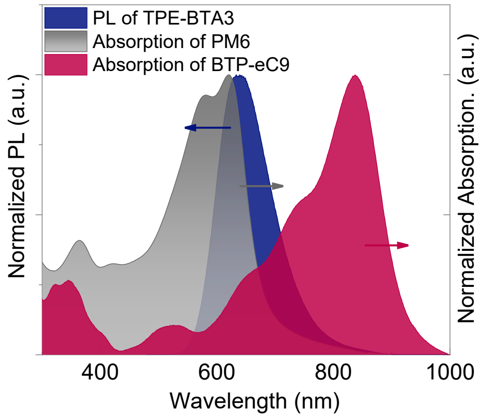

Fig. 2d. PL spectra of TPE-BTA3, absorption spectra of PM6, and BTP-eC9.

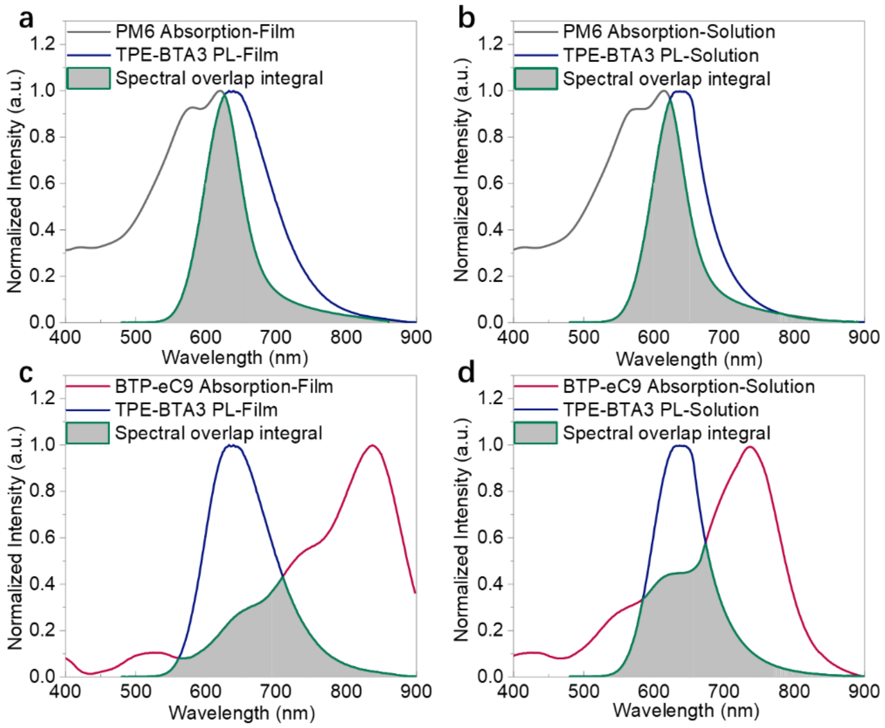

Fig. R1. The schematic diagrams of spectral overlap, PM6/BTP-eC9's absorption spectrum with TPE-BTA3's PL spectrum in film and solution states.

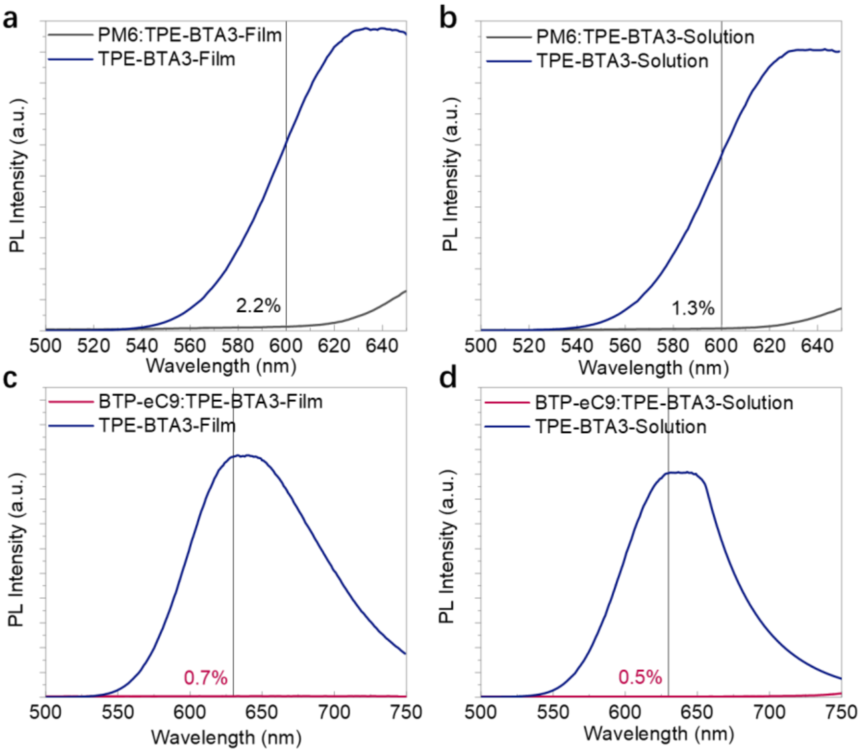

Fig. R2. PL spectrum of TPE-BTA3 and PM6/BTP-eC9:TPE-BTA3 in film and solution states.

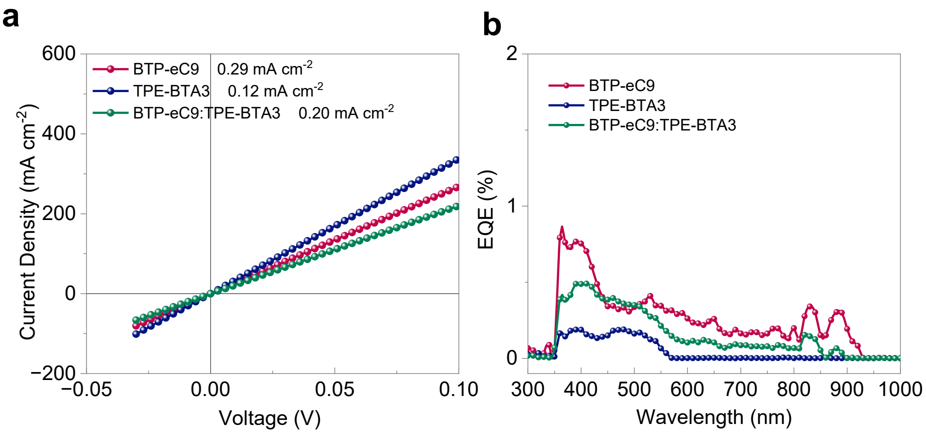

Fig. S10. a, J-V curves of devices with BTP-eC9, TPE-BTA3, and BTP-eC9:TPE-BTA3 blend films as the active layers. b, EQE curves of devices with BTP-eC9, TPE-BTA3, and BTP-eC9:TPE-BTA3 blend films as the active layers.

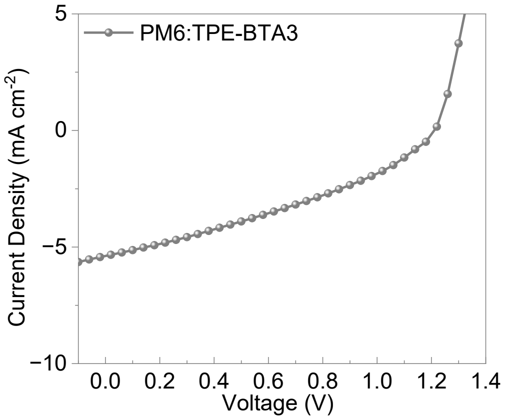

Fig. S15a. J-V curves of PM6:TPE-BTA3 based OSC.

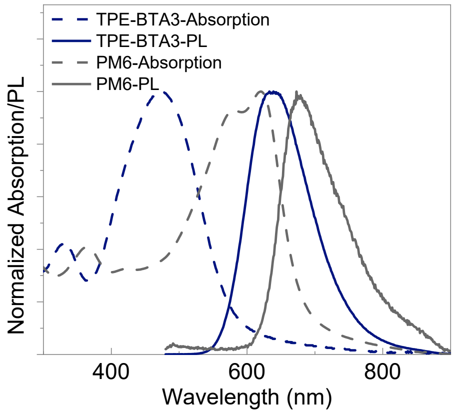

Fig. R3. Normalized absorption/PL spectra of TPE-BTA3 and PM6.

We thank the reviewer for prompting this more nuanced understanding. We have added a qualitative discussion on Förster resonance energy transfer and Dexter energy transfer on page 11 of the revised manuscript. "Energy transfer is categorized into Förster resonance energy transfer (FRET) and Dexter energy transfer (DET). FRET is

a non-contact, long-range process mediated by dipole–dipole interactions, typically effective over distances of 1–10 nm. It requires substantial spectral overlap between the donor's emission and the acceptor's absorption, as well as a high PLQY of the donor. In contrast, DET is a short-range, contact-dependent process involving electron exchange, with an effective distance of less than 1 nm. It necessitates direct orbital overlap between donor and acceptor and matching spin states. In the PM6:BTP-eC9:TPE-BTA3 system studied here, TPE-BTA3 serves as the energy donor and exhibits a high PLQY. Its fluorescence emission spectrum shows significant overlap with the absorption spectra of both PM6 and BTP-eC9, as illustrated in **Fig. 2d**. Based on these characteristics, we propose that FRET should be the dominant mechanism in this system."

5. Statistical significance of energy-loss reduction

The reduction in non-radiative energy loss ΔE_3 (0.229 → 0.217 eV) and the small change in ΔE_2 (0.057 → 0.052 eV) are central to the manuscript's conclusions. However, error bars and statistical significance are not provided. The authors should report device-to-device statistics, including mean values and standard deviations, and clearly describe measurement calibration procedures, such as EQE spectral integration, light-source calibration, and injected current ranges.

Response: We appreciate your suggestion. As suggested, we have supplemented the statistical data in **Table S5** and the detailed calibration procedure before the measurement.

To accurately assess the energy losses in the devices, we statistically analyzed the ΔE_3 and ΔE_2 based on five groups of devices, as summarized in **Table S5**. The average ΔE_3 values for the ternary and binary devices are 0.229 ± 0.001 and 0.218 ± 0.001 eV, respectively, while the corresponding ΔE_2 values are 0.057 ± 0.001 eV and 0.052 ± 0.001 eV.

The determination of ΔE_3 relies on the measurement of the EQE_{EL}, which requires precise calibration of the optical system before measurement. Specifically, the calibration procedure involves placing a standard halogen lamp with a known spectral irradiance at the same position within the integrating sphere as the device under test. The emission spectrum of the standard lamp is recorded by the spectrometer, enabling the derivation of the system's spectral responsivity function. Subsequently, the OSC is driven by a Keithley source measure unit at a constant injected current. The electroluminescence spectrum is collected using the integrating sphere and recorded as a raw signal. This raw signal is then converted to absolute spectral radiance based on the pre-determined responsivity function. The total emitted photon flux is obtained by numerically integrating the spectral radiance across the entire emission spectrum. Finally, EQE_{EL} is calculated as the ratio of the emitted photon flux to the injected electron flux.

Regarding the selection of the injection current range, all measurements were performed at low current densities (typically between 0.1 and several tens of mA/cm^2) to minimize artifacts such as Joule heating, series resistance effects, and device degradation. It is necessary to ensure that the EQE_{EL} values measured at different

currents remain constant (plateau region), indicating that measurements are performed within the stable region of the recombination mechanism.

We have incorporated the statistical data of energy-loss on page 12 of the revised manuscript and the details of the measurement on page 10 of the Supplementary Information.

Table S5. Statistical energy loss of PM6:BTP-eC9 and PM6:BTP-eC9:TPE-BTA3.

Compound	ΔE_3 (eV)	ΔE_2 (eV)
PM6:BTP-eC9	0.229±0.001	0.057±0.001
PM6:BTP-eC9: TPE-BTA3	0.218±0.001	0.052±0.001

6. Urbach energy analysis

The reported decrease in Urbach energy (0.024 → 0.022 eV) is rather small. Additional experimental or statistical evidence is needed to convincingly demonstrate a meaningful reduction in energetic disorder.

Response: We appreciate the reviewer's valuable comment. For ternary organic solar cells, certain performance parameters do not differ substantially from those of their binary counterparts. We further analyzed the Urbach energy based on conventional EQE spectra, and the differences in Urbach energy were found to be insufficient to demonstrate a meaningful reduction in energetic disorder. To ensure scientific rigor, we have therefore removed the discussion on the Urbach energy from the revised manuscript.

Urbach energy can be calculated not only via highly sensitive EQE (s-EQE) spectra but also from conventional EQE spectra. We performed new calculations using the conventional EQE spectrum shown in **Fig. 2b** of the revised manuscript. We converted the horizontal axis to photon energy and applied a natural logarithm to the EQE values, as shown in **Fig. R4**. The band-edge tail region follows the relation: Eu = (E1 - E2) / (lnA1 - lnA2), where E denotes photon energy, and A represents the corresponding EQE intensity. From linear fitting of the tail region, the slopes for the ternary and binary devices were determined to be 39.5 and 37.1, respectively, yielding Urbach energies of 0.025 eV and 0.027 eV.

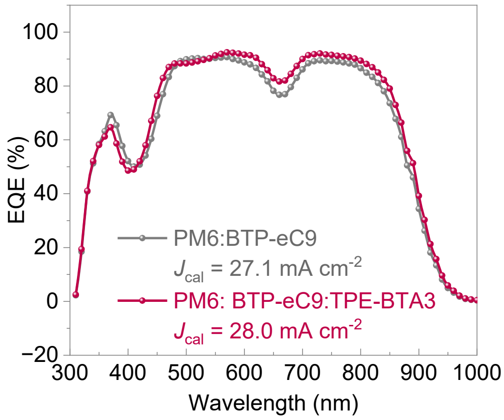

Fig. 2b. Conventional EQE curves of binary and ternary devices.

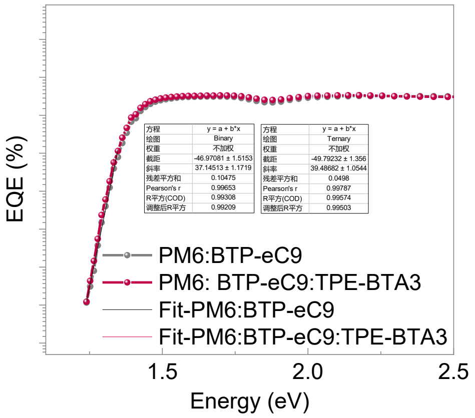

Fig. R4. Variations from Fig. 2b, which has converted the horizontal axis to photon energy and applied a natural logarithm to the EQE values.

7. Electrical characterization of tandem devices

The tandem architecture (ITO/4PADCB/perovskite/C60/BCP/Au/MoO_3/2-PACz/organic/PNDIT-F3N/Ag) is supported mainly by cross-sectional SEM (Fig. S24). However, electrical validation of the tandem device is insufficient. The authors are encouraged to provide measurements of contact resistance, series resistance, or impedance spectroscopy, as well as a discussion of the energy-level alignment and interfacial chemical stability of the Au/MoO_3/2-PACz recombination layer.

Response: We thank the reviewer for the valuable suggestion. We have performed a statistical analysis of the series resistance from ten individual single-junction PSCs, single-junction OSCs, and TSCs. As shown in **Fig. S29**, the average series resistance of OSCs, PSCs, and TSCs is 37.6±4.6, 51.8±4.8, and 91.4±4.6 Ω, respectively. Notably, the series resistance of the tandem device is approximately equal to the sum of those of the two sub-cells, indicating an excellent electrical conductivity of the interconnecting layers (Au/MoO_3/2-PACz) for carrier recombination. The resistance values were derived using the IVS-KA6000 software (Enlitech) based on the single-diode equivalent circuit model, which follows the equation:

$$I = I_L - I_0\left[\exp\left(\frac{q(V + IR_s)}{nkT}\right) - 1\right] - \frac{V + IR_s}{R_{sh}}$$

Where I, V, R_s, R_{sh}, I_0, n, q, k, and T are the output current, output voltage, series resistance, shunt resistance, reverse saturation current of the diode, ideal factor, elementary charge, Boltzmann constant, and absolute temperature.

Fig. S30 further demonstrates the energy level diagram of the tandem solar cell, which reveals a favorable energy level alignment for hole extraction toward the ITO electrode, electron extraction toward the Ag electrode, and effective electron–hole recombination at the Au-based recombination layers.

For the stability of the internal recombination layers (Au/MoO_3/2-PACz), the metal Au with a high work function of -5.1 eV always demonstrates a superior stability. On the other hand, the phosphate group in 2-PACz tends to anchor onto the MoO_3 surface,

forming a robust Mo–O–P coordinate bond that enhances structural stability. Density functional theory calculations also reveal a strong binding energy of 3.44 eV between the MoO_3 and 2-PACz, as shown in **Fig. S31**.

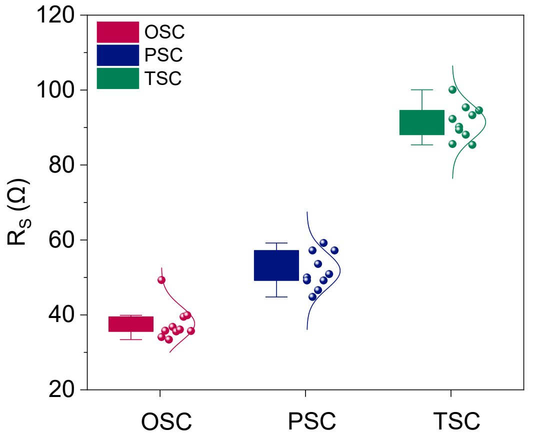

Fig. S29. The statistical series resistance values of OSCs, PSCs, and TSCs.

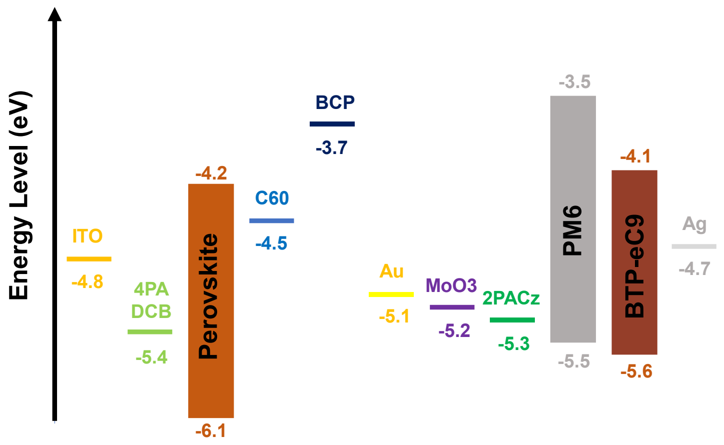

Fig. S30. The energy level diagram of the complete tandem device.

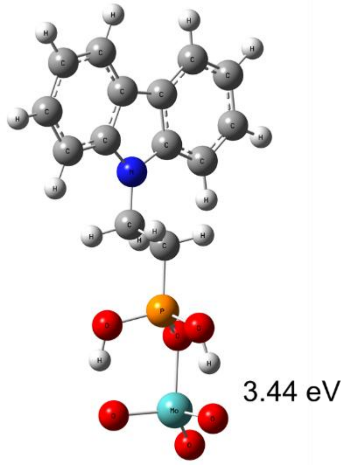

Fig. S31. The Mo–O–P bond connects 2PACz with MoO_3, as obtained through first-principles density functional theory.

We have incorporated related discussions on page 16 of the revised manuscript

and page 22 of the Supplementary information. "Benefiting from the excellent photovoltaic performance, we further investigated the properties of the interconnecting recombination layer Au/ MoO_3/2-PACz. As shown in **Fig. S29**, the average series resistances of OSCs, PSCs, and TSCs are 37.6±4.6, 51.8±4.8, and 91.4±4.6 Ω, respectively. Notably, the series resistance of the tandem device is approximately equal to the sum of those of the two sub-cells, demonstrating the excellent electrical conductivity of the interconnecting layer for efficient carrier recombination. The energy level diagram of the TSC (**Fig. S30**) further reveals a favorable energy level alignment for carrier extraction toward the external electrodes, as well as effective electron–hole recombination at the internal recombination layer. In addition, the phosphate group in 2-PACz tends to anchor onto the MoO_3 surface, forming a robust Mo–O–P coordinate bond that enhances structural stability. Density functional theory calculations also confirm a strong binding energy of 3.44 eV between MoO_3 and 2-PACz, as illustrated in **Fig. S31**."

8. Stability assessment and broader tandem perspective

The reported stability tests are relatively short. Longer-term stability measurements are recommended, with clear specification of temperature control, spectral mismatch correction, and active vs aperture area. In addition, the authors should discuss the advantages and limitations of:

o perovskite–organic tandems,

o perovskite–perovskite tandems,

o perovskite–silicon tandems,

as well as the corresponding performance-improvement strategies. The rationale for considering perovskite–organic tandem solar cells as a promising route should be clearly articulated.

Response: We appreciate your valuable suggestion. As suggested, we have extended the aging test to 1000 hours, as shown in the updated **Fig. 4e**. The TSC incorporating TPE-BTA3 exhibits a T80 lifetime exceeding 600 hours, representing an improvement compared with the TSC without TPE-BTA3.

Photostability of the encapsulated devices was tested using a full-spectrum white LED lamp with a wavelength range from 350 to 1050 nm (**Fig. R5**) with a device temperature of approximately 60 °C in ambient air. The light intensity of the LED lamp was calibrated to 100 mW/cm^2. We did not conduct spectral mismatch correction during the stability test.

Among various perovskite-based TSCs, perovskite-silicon TSCs offer ideal bandgap matching, high power conversion efficiency, and accessible rapid commercialization via the mature silicon photovoltaic industrial chain, but their inherent rigidity limits applications in flexible and lightweight scenarios. All-perovskite TSCs enable low-temperature solution processing and flexible device fabrication, yet the easy oxidation of tin-based narrow-bandgap perovskites severely impedes their commercial feasibility. By contrast, perovskite-organic TSCs inherit the core advantages of all-perovskite TSCs, including solution processability and excellent mechanical flexibility. Notably, the use of mutually orthogonal solvents in fabrication circumvents the need for complex techniques like atomic layer deposition, simplifying manufacturing. Admittedly, the current efficiency of such devices is limited by the performance of narrow-bandgap

organic materials, but with the design and synthesis of novel high-performance organic photovoltaic materials, perovskite-organic TSCs hold extremely promising development prospects.

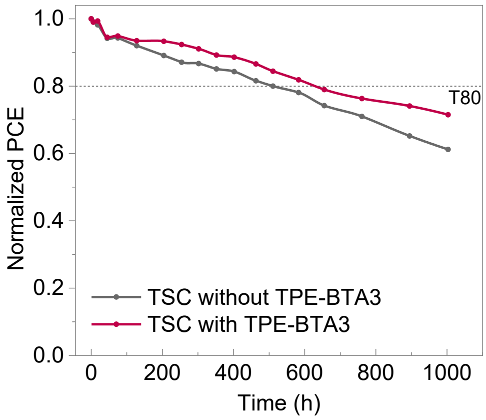

Fig. 4e. Long-term photostability of encapsulated tandem devices under continuous one-sun illumination in ambient.

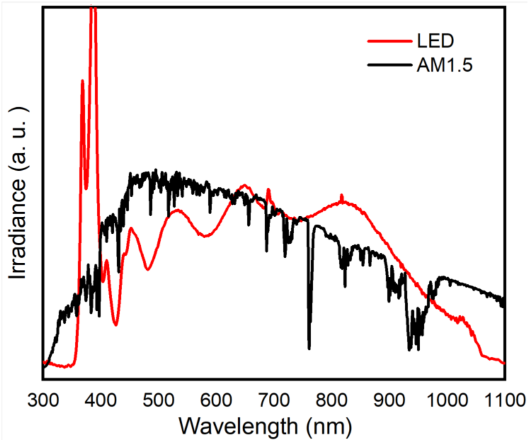

Fig. R5. The spectra of the white LED light source used in the long-term photostability test.

We have added related discussions on page 3 of the manuscript. "Y-series organic semiconductors with a narrow bandgap of 1.3-1.4 eV enable them to be particularly suitable for constructing perovskite-organic tandem solar cells (TSCs), which own unique advantage among various perovskite-based TSCs. The perovskite-silicon TSCs offer ideal bandgap matching, high power conversion efficiency, and accessible rapid commercialization via the mature silicon photovoltaic industrial chain, but their inherent rigidity limits applications in flexible and lightweight scenarios. All-perovskite TSCs enable low-temperature solution processing and flexible device fabrication, yet the easy oxidation of tin-based narrow-bandgap perovskites severely impedes their commercial feasibility. By contrast, perovskite-organic TSCs inherit the core advantages of all-perovskite TSCs, including solution processability and excellent mechanical flexibility. Notably, the use of mutually orthogonal solvents in fabrication circumvents the need for complex techniques like atomic layer deposition, simplifying

manufacturing. Admittedly, the current efficiency of such devices is limited by the performance of narrow-bandgap organic materials, but with the design and synthesis of novel high-performance organic photovoltaic materials, perovskite-organic TSCs hold extremely promising development prospects.”

9. Control experiments to isolate the AIE effect

Since the authors attribute the performance enhancement primarily to the AIE effect of TPE-BTA3, it is essential to rule out a generic additive effect. The authors should therefore fabricate single-junction and tandem devices using structurally similar third components that do not exhibit AIE behavior, and compare their performance accordingly.

Response: We appreciate your valuable suggestion. To further verify that the improvement in PCE originates from the AIE effect, we introduced BTA3, a structurally analogous molecule without AIE activity (**Fig. S34**), as a third component into the PM6:BTP-eC9 system for control experiments. As illustrated in **Fig. S35**, the addition of BTA3 did not enhance the photovoltaic performance of either single-junction or tandem devices; instead, it led to a clear decrease in device efficiency. These results strongly confirm that the AIE effect of TPE-BTA3 is responsible for the effectively improved photovoltaic performance. The corresponding photovoltaic parameters of single-junction and tandem devices are summarized in **Table S10**.

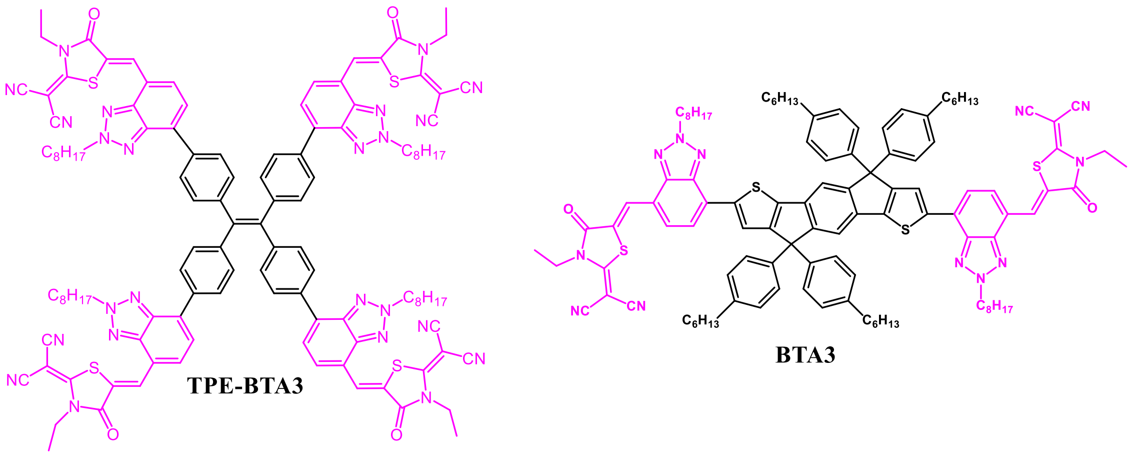

Fig. S34. The molecular structures of TPE-BTA3 and BTA3.

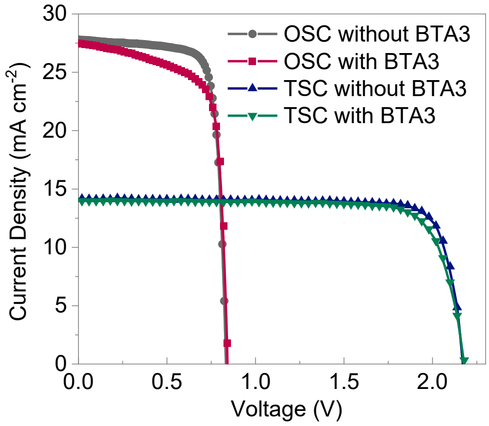

Fig. S35. *J-V* curves of single-junction OSCs and perovskite-organic TSCs with and without BTA3 measured under AM1.5G illumination.

Table S10. Photovoltaic parameters of OSCs and TSCs without/with BTA3.

Compound	V_{OC} (V)	J_{SC} (mA cm^{-2})	FF (%)	PCE (%)
PM6:BTP-eC9 1:1.2	0.834	27.8	78.7	18.3
PM6:BTP-eC9: BTA3 1:1.2:0.12	0.842	27.5	73.4	17.0
TSC Without BTA3	2.177	14.2	82.4	25.4
TSC With BTA3	2.182	14.0	79.4	24.3

We have added related discussions on page 17 of the manuscript. "To further verify that the improvement in PCE originates from the AIE effect, we introduced BTA3, a structurally analogous molecule without AIE activity (**Fig. S34**), as a third component into the PM6:BTP-eC9 system for control experiments. As illustrated in **Fig. S35**, the addition of BTA3 did not enhance the photovoltaic performance of either single-junction or tandem devices; instead, it led to a clear decrease in device efficiency. These results strongly confirm that the AIE effect of TPE-BTA3 is responsible for the effectively improved photovoltaic performance. The corresponding photovoltaic parameters of single-junction and tandem devices are summarized in **Table S10**."
